# The global burden of falls: global, regional and national estimates of morbidity and mortality from the Global Burden of Disease Study 2017

**DOI:** 10.1136/injuryprev-2019-043286

**Published:** 2020-01-15

**Authors:** Spencer L James, Lydia R Lucchesi, Catherine Bisignano, Chris D Castle, Zachary V Dingels, Jack T Fox, Erin B Hamilton, Nathaniel J Henry, Kris J Krohn, Zichen Liu, Darrah McCracken, Molly R Nixon, Nicholas L S Roberts, Dillon O Sylte, Jose C Adsuar, Amit Arora, Andrew M Briggs, Daniel Collado-Mateo, Cyrus Cooper, Lalit Dandona, Rakhi Dandona, Christian Lycke Ellingsen, Seyed-Mohammad Fereshtehnejad, Tiffany K Gill, Juanita A Haagsma, Delia Hendrie, Mikk Jürisson, G Anil Kumar, Alan D Lopez, Tomasz Miazgowski, Ted R Miller, GK Mini, Erkin M Mirrakhimov, Efat Mohamadi, Pedro R Olivares, Fakher Rahim, Lidia Sanchez Riera, Santos Villafaina, Yuichiro Yano, Simon I Hay, Stephen S Lim, Ali H Mokdad, Mohsen Naghavi, Christopher J L Murray

**Affiliations:** 1 Institute for Health Metrics and Evaluation, University of Washington, Seattle, Washington, USA; 2 Sport Science Department, University of Extremadura, Badajoz, Spain; 3 School of Science and Health, Western Sydney University, Sydney, NSW, Australia; 4 Oral Health Services, Sydney Local Health District, Sydney, NSW, Australia; 5 School of Physiotherapy and Exercise Science, Curtin University, Bentley, WA, Australia; 6 Ageing and Life Course, World Health Organization (WHO), Geneva, Switzerland; 7 Sport Science Department, University of Extremadura, Cáceres, Spain; 8 Faculty of Education, Autonomous University of Chile, Talca, Chile; 9 Medical Research Council Lifecourse Epidemiology Unit, University of Southampton, Southampton, United Kingdom; 10 Department of Rheumatology, University of Oxford, Oxford, United Kingdom; 11 Public Health Foundation of India, Gurugram, India; 12 Department of Pathology, Stavanger University Hospital, Stavanger, Norway; 13 Norwegian Institute of Public Health, Oslo, Norway; 14 Department of Neurobiology, Care Sciences and Society, Karolinska Institutet, Stockholm, Sweden; 15 Division of Neurology, University of Ottawa, Ottawa, ON, Canada; 16 Adelaide Medical School, University of Adelaide, Adelaide, SA, Australia; 17 Department of Public Health, Erasmus University Medical Center, Rotterdam, The Netherlands; 18 School of Public health, Curtin University, Perth, Western Australia, Australia; 19 Institute of Family Medicine and Public Health, University of Tartu, Tartu, Estonia; 20 University of Melbourne, Melbourne, QLD, Australia; 21 Department of Hypertension, Pomeranian Medical University, Szczecin, Poland; 22 Pacific Institute for Research and Evaluation, Calverton, Maryland, USA; 23 Achutha Menon Centre for Health Science Studies, Sree Chitra Tirunal Institute for Medical Sciences and Technology, Trivandrum, India; 24 Global Institute of Public Health (GIPH), Ananthapuri Hospitals and Research Centre, Trivandrum, India; 25 Faculty of General Medicine, Kyrgyz State Medical Academy, Bishkek, Kyrgyzstan; 26 Department of Atherosclerosis and Coronary Heart Disease, National Center of Cardiology and Internal Disease, Bishkek, Kyrgyzstan; 27 Health Equity Research Center, Tehran University of Medical Sciences, Tehran, Iran; 28 Institute of Physical Activity and Health, Autonomous University of Chile, Talca, Chile; 29 Thalassemia and Hemoglobinopathy Research Center, Ahvaz Jundishapur University of Medical Sciences, Ahvaz, Iran; 30 Endocrinology and Metabolism Molecular-Cellular Sciences Institute, Tehran University of Medical Sciences, Tehran, Iran; 31 Department of Rheumatology, University Hospitals Bristol NHS Foundation Trust, Bristol, UK; 32 Institute of Bone and Joint Research, University of Sydney, Syndey, NSW, Australia; 33 Department of Preventive Medicine, Northwestern University, Chicago, IL, United States; 34 Department of Health Metrics Sciences, School of Medicine, University of Washington, Seattle, WA, USA

**Keywords:** fall, epidemiology, burden of disease

## Abstract

**Background:**

Falls can lead to severe health loss including death. Past research has shown that falls are an important cause of death and disability worldwide. The Global Burden of Disease Study 2017 (GBD 2017) provides a comprehensive assessment of morbidity and mortality from falls.

**Methods:**

Estimates for mortality, years of life lost (YLLs), incidence, prevalence, years lived with disability (YLDs) and disability-adjusted life years (DALYs) were produced for 195 countries and territories from 1990 to 2017 for all ages using the GBD 2017 framework. Distributions of the bodily injury (eg, hip fracture) were estimated using hospital records.

**Results:**

Globally, the age-standardised incidence of falls was 2238 (1990–2532) per 100 000 in 2017, representing a decline of 3.7% (7.4 to 0.3) from 1990 to 2017. Age-standardised prevalence was 5186 (4622–5849) per 100 000 in 2017, representing a decline of 6.5% (7.6 to 5.4) from 1990 to 2017. Age-standardised mortality rate was 9.2 (8.5–9.8) per 100 000 which equated to 695 771 (644 927–741 720) deaths in 2017. Globally, falls resulted in 16 688 088 (15 101 897–17 636 830) YLLs, 19 252 699 (13 725 429–26 140 433) YLDs and 35 940 787 (30 185 695–42 903 289) DALYs across all ages. The most common injury sustained by fall victims is fracture of patella, tibia or fibula, or ankle. Globally, age-specific YLD rates increased with age.

**Conclusions:**

This study shows that the burden of falls is substantial. Investing in further research, fall prevention strategies and access to care is critical.

## Introduction

Falls are one of the most common mechanisms of injury and endure as a persistent risk to morbidity and mortality across all ages. The risk of an injurious fall in a population as well as the resulting disability may be governed by a wide array of factors ranging from drug and alcohol intoxication in younger populations to frailty and comorbidities in older adult populations. Falls pose sufficient risk in modern high-resource healthcare settings to necessitate the use of safety devices such as bed alarms and traction socks in inpatient wards and dedicated physical and occupational therapy services. Falls in young, otherwise healthy populations can produce lifelong disability in the form of traumatic brain injuries or spinal cord injuries and can also cause severe injuries that necessitate advanced surgical care, such as intra-abdominal organ injury or complicated skeletal fractures.^1^ In older populations, the morbidity experienced by falls may be further modulated by comorbid conditions such as osteoporosis, osteopenia, or usage of anticoagulant or antiplatelet medications.^2 3^ Given that many fall incidents are preventable, occur in any population and can lead to substantial morbidity and mortality, it is surprising that falls do not draw more attention as an important global issue.

In the Global Burden of Diseases, Injuries and Risk Factors Study 2017 (GBD 2017), global estimates of the burden of falls show that falls were ranked as the 18th leading cause of age-standardised rates of disability-adjusted life years in 2017, outranking conditions such as chronic kidney disease, Alzheimer’s disease and other dementias, and asthma.^4^ Additionally, falls were noted to be the second leading cause of death due to unintentional injuries in 2017, following road injuries and outranking causes such as interpersonal violence and drowning.^5^ Research outside of the GBD Study on the epidemiology of falls has largely focused on older populations as this is where the global burden of falls is thought to be most concentrated. The World Health Organization (WHO) reports that most deaths from falls happen in those aged 65 and older.^6^ For those 70 years or older, falls are the leading category in injury-related deaths.^7^ With a burden highly concentrated in older adults, many recent studies have discussed the effects of population ageing, recognising the potential for far more incident cases and deaths from falls as people live longer.^8–10^ In addition, several studies have focused on younger populations as they are an important high-risk group to consider as well. An injury surveillance system pilot study conducted in 4 low/middle-income countries found that falls accounted for the largest percentage (56%) of recorded injuries among children.^11^ A study conducted in India similarly found that the most common type of home injury in children aged 0–14 was falling.^12^

Given the known extent of this burden, it is important to measure and understand how the burden of falls is distributed in terms of morbidity and mortality, across all age groups and between both sexes, and in every geographical region of the world. In addition, since the disability that results from falls may vary by location, it is of interest to systematically measure how the distribution of injuries resulting from falls varies by region.

The GBD Study represents the efforts of a global research collaboration that produces comprehensive estimates of hundreds of diseases, injuries and risk factors in 195 countries and territories using data and methods that are updated on an annual basis, most recently in GBD 2017. The specific estimates produced by the GBD include annual estimates of all-cause mortality, causes of death, non-fatal health outcomes (ie, incidence, prevalence and years lived with disability (YLDs)) and risk factors. These measures are estimated for all countries and territories, age groups and sexes, across a range of years. The intent of providing this level of estimation detail is to allow focused and nuanced analyses of death and disability across demographics, locations and causes of injuries. Falls is a category of injury in the GBD cause hierarchy and was included in the GBD 2017 results, but to date there have been no known studies that examined the findings for this cause in detail. Additionally, the injuries resulting from falls have not previously been reported using GBD 2017 results.

In this study, we use the GBD 2017 framework to analyse the morbidity and mortality caused by falls as reported in GBD 2017 and explore the burden of injuries resulting from falls.

## Methods

### GBD Study 2017

Methods used in the GBD Study 2017 have been described in extensive detail elsewhere, including description of the analytical estimation framework used to measure mortality, incidence, prevalence, years of life lost (YLLs), years lived with disability (YLDs) and disability-adjusted life years (DALYs).^4 5 13–16^ Online supplementary appendix 1 provides a methodological overview of different components used in the GBD Study design and analytical framework. The methodological components specific to the estimation of falls within the GBD framework are summarised below.

10.1136/injuryprev-2019-043286.supp1 Supplementary data

### GBD injury classification

The GBD 2017 reported estimates in terms of *external cause* of injury (eg, falls) and measured disability based on *nature* of injury (eg, hip fracture). Causes of injury were defined in accordance with the International Classification of Diseases (ICD). For this study, falls were defined as ICD-9 codes E880–E886, E888 and ICD-10 codes W00–W19.9. In terms of the nature-of-injury codes, falls had 47 mutually exclusive and collectively exhaustive nature-of-injury categories which were specified with chapters S and T in ICD-10 and codes 800–999 in ICD-9 to quantify the various disabling outcomes that can occur with a fall.

### Mortality and YLLs due to falls

For deaths due to falls, we estimated both mortality and YLLs due to premature mortality. Our approach for estimating causes of death for every cause, including falls, is provided in the GBD 2017 cause of death literature.^17^

First, we identified and obtained all available cause-of-death data sources. These sources included complete vital registration systems shared by countries; verbal autopsy studies published in literature; and mortality surveillance, censuses, surveys, hospital records and mortuary data. The cause of death estimates from these sources were mapped to the GBD cause list such that the corresponding ICD codes listed above were mapped to our ‘falls’ cause, as were non-ICD-coded reporting systems where ‘falls’ were designated as a cause of death, for example, in verbal autopsy studies which are typically not ICD coded but include a textual cause list.

Second, we conducted estimation models using the GBD Cause of Death Ensemble model (CODEm) to estimate cause-specific mortality for falls by age, sex, country and year. CODEm is an ensemble modelling approach for producing a large variety of possible models to estimate trends in causes of death using an algorithm that selects a wide array of combinations of covariates and different modelling methods.^18^

Third, we calculated YLLs by multiplying deaths by the residual life expectancy using the global maximum life expectancy at the age of death as derived from the GBD standard model life table. For example, if an individual dies at age 60 from a fall and their residual life expectancy is 20 years, then there were 20 YLLs due to that fall.

### Injury incidence, prevalence and YLDs

The method for estimating non-fatal injury outcomes including falls in GBD 2017 is described in more detail in related publications.^19^ A methodological summary is as follows.

First, we used DisMod-MR 2.1 to measure incidence of falls that lead to any form of medical care (inpatient or outpatient). DisMod-MR 2.1 is a meta-regression tool for epidemiological modelling built on a Bayesian compartmental model framework that solves differential equations that modulate the relationships between a susceptible population becoming injured (incidence) and then either recovering (remission) or dying (excess mortality). For incidence data, we used emergency department records, hospital records, survey data and literature studies to estimate fall incidence by location, year, age and sex, and used the coefficient from outpatient care to split subsequent estimation processes into inpatient and outpatient incidence estimates so that inpatient and outpatient-specific data could be used where possible to preserve differences in incidence and severity. Since survey items for falls can include non-injurious falls, we included an indicator variable for falls that resulted in injury. Since excess mortality is calculated based on locations where there are overlapping incidence and cause-specific mortality data, its computation also allows for estimation of incidence in locations with cause-specific mortality data but no incidence data, requiring an assumption that case fatality rates among falls are affected by income.

Second, we estimated the distribution of nature-of-injury categories among the incidence of all falls. To do this, we created a hierarchy of nature-of-injury categories. We assumed that the disability experienced by an individual who has an injurious fall was determined by the most severe nature-of-injury sustained due to this fall. For example, a fall resulting in a spinal cord injury would determine disability due to the fall instead of a co-occurring wrist sprain. The nature-of-injury hierarchy represents a combination of the likelihood of long-term disability and the corresponding GBD disability weight. To estimate the hierarchy, we used data from pooled follow-up studies in which we translated each individual’s health status measure at 1 year after injury into a disability weight.^20–26^

Third, we used a Dirichlet regression method to estimate the proportion of falls that result in each nature-of-injury category being the most severe injury for each fall, since Dirichlet methods enforce coefficient estimates for proportions that must sum to 1.^27^ These matrices were derived from dual-coded hospital and emergency department data sets from multiple countries and data from the China injury surveillance system where both cause-of-injury and nature-of-injury diagnosis codes are present. The use of these data sources to inform this estimation process is described in more detail elsewhere.^1 28^ Separate cause-nature matrices were created for falls warranting hospital admission versus falls warranting other healthcare, high and low-income countries, male and female, and age category.

Fourth, we estimated short-term disability for falls by nature‐of‐injury category. For each nature-of-injury category and inpatient and outpatient injury, we used the Dutch Injury Surveillance System to derive average duration for treated cases, since for GBD 2017 this was the only available data source that could inform this parameter.^23 24^ These estimates were supplemented by expert-driven estimates of short-term duration for nature-of-injury categories that had insufficient numbers in the Dutch data set and for untreated injuries.

Fifth, we estimated the proportion of falls resulting in permanent disability for each nature-of-injury category by admission status and age. Disability due to falls was assumed to affect all injurious falls in the short term with a proportion having long-term (permanent) outcomes, defined as having persistent disability 1 year after the injury greater than the preinjury health status.

Sixth, we applied the ordinary differential equation solver used as the computational engine in DisMod-MR 2.1 to estimate the long-term prevalence for each fall-related nature-of-injury from incidence and the long-term mortality risk in cases with long-term disability based on meta-analyses of studies providing standardised mortality ratios. For example, since individuals with severe traumatic brain injuries die at a higher rate than the underlying population, we integrated the corresponding standardised mortality ratios to account for decreasing prevalence due to higher mortality risk in this injured population.

Finally, we calculated YLDs as prevalence of each health state multiplied by a disability weight for each nature-of-injury and corrected for comorbidity with other non-fatal diseases using microsimulation methods employed in GBD 2017.

### Socio-demographic index

Socio-demographic index (SDI) is a composite indicator of development that is calculated based on income per capita, average educational attainment over age 15 and total fertility rate under age 25.^15^ The SDI has a scale that ranges from 0 representing the lowest income *per capita*, lowest educational attainment and highest fertility observed across all GBD locations from 1980 to 2017, to 1, representing the point at which the higher income per capita, higher educational attainment and lower fertility under age 25 are no longer associated with improved health. We used SDI values for each country and territory to categorise our estimates in this study by SDI quintile to help illustrate how burden trends differ by development level.

#### GATHER compliance

This study complies with theGuidelines for Accurate and Transparent Health Estimates Reporting (GATHER)recommendations (online supplementary appendix 2). Analyses were completed using Python version 2.7, Stata version 13.1, or R version 3.3. Statistical code used for GBD estimationis publicly available online at healthdata.org.

10.1136/injuryprev-2019-043286.supp2 Supplementary data

## Results

Results tables are listed as web appendix tables. Results by age, sex, year, subnational location and nature of injury are also available online via the GBD Results Tool (http://ghdx.healthdata.org/gbd-results-tool) and GBD Compare (https://vizhub.healthdata.org/gbd-compare/).

### Incidence

**Figure 1:**
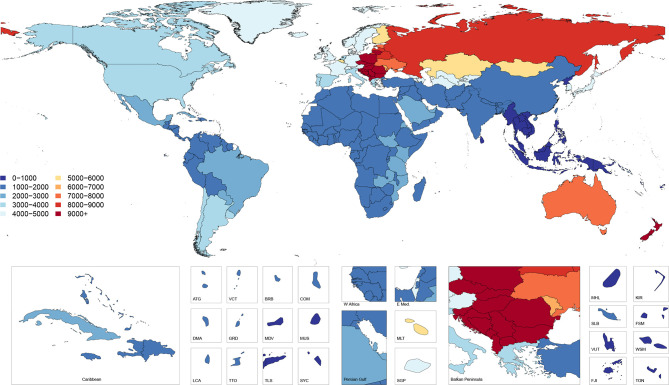
Age-standardised incidence rates per 100 000 of falls, 2017, both sexes.

Figure 1 shows age-standardised incidence of falls by country and territory in 2017. This map illustrates the higher incidence rates in Eastern and Central European countries as well as Australia and New Zealand. Online supplementary appendix table 1 shows the all-ages incidence counts and the age-standardised incidence rates for 2017 as well as the percentage change in age-standardised rates from 1990 to 2017. Globally, the age-standardised incidence rate was 2238 (95% uncertainty interval 1990 to 2532) per 100 000 in 2017, representing a decline of 3.7% (7.4 to 0.3) from 1990 to 2017, and equating to 171 691 220 (152 472 652–194 061 874) new injuries from falls in 2017. The age-standardised incidence rate decreased in the high-middle and high SDI quintiles and increased in the middle, low-middle and low SDI quintiles. The largest decline was in the high SDI quintile, which decreased by 8.8% (−12.3 to −5.3). The geographic regions with the highest age-standardised incidence rates were Central Europe with 11 434 (10 103–12 996) cases per 100 000, Australasia with 8187 (6978–9553) cases per 100 000 and Eastern Europe with 8029 (7010–9233) cases per 100 000. Among the 21 GBD regions, 12 experienced significant increases in age-standardised incidence rates (Australasia, High-income Asia Pacific, Andean Latin America, Caribbean, Central Latin America, Tropical Latin America, South Asia, East Asia, Oceania, Southeast Asia, Central Sub-Saharan Africa, Southern Sub-Saharan Africa), 2 experienced significant decreases (Central Europe, High-income North America) and the remaining 7 regions experienced no significant change in age-standardised incidence rates (Central Asia, Eastern Europe, Southern Latin America, Western Europe, North Africa and Middle East, Western Sub-Saharan Africa).

10.1136/injuryprev-2019-043286.supp3 Supplementary data

### Prevalence

Online supplementary appendix table 1 also shows the all-ages prevalence counts and the age-standardised prevalence rate for 2017 as well as the percentage change in age-standardised prevalence from 1990 to 2017. Globally, the age-standardised prevalence rate was 5186 (4622–5849) per 100 000 in 2017, representing a decline of 6.5% (7.6 to 5.4) from 1990 to 2017. There were 411 711 999 (366 390 987–465 354 952) prevalent cases in 2017. East Asia had the highest number of prevalent cases in 2017 with 62 282 056 (54 985 517–70 760 535) cases across all ages and both sexes. The age-standardised prevalence decreased in the high and high-middle SDI quintiles and increased in the low, low-middle and middle SDI quintiles. The regions with the highest age-standardised prevalence were Central Europe with 23 428 (20 453–26 911) cases per 100 000, Eastern Europe with 17 429 (15 114–20 228) cases per 100 000 and Australasia with 16 175 (13 641–19 647) cases per 100 000. Among the 21 GBD regions, 14 experienced significant increases in age-standardised prevalence rates (East Asia, Oceania, Tropical Latin America, South Asia, Caribbean, Andean Latin America, Australasia, Southeast Asia, High-income Asia Pacific, Southern Sub-Saharan Africa, Central Latin America, Central Sub-Saharan Africa, Eastern Europe, Eastern Sub-Saharan Africa), 6 experienced significant decreases in age-standardised prevalence rates (High-income North America, Southern Latin America, Central Asia, Western Sub-Saharan Africa, Central Europe, Western Europe) and the remaining region experienced no significant change in age-standardised prevalence (North Africa and Middle East).

### Cause-specific mortality

**Figure 2:**
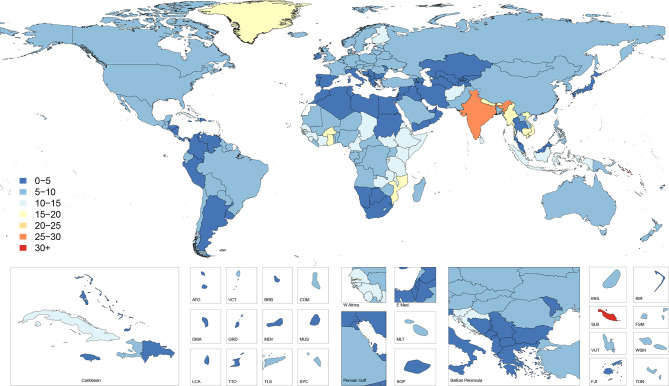
Age-standardised cause-specific mortality rate per 100 000 of falls, 2017, both sexes.

**Figure 3:**
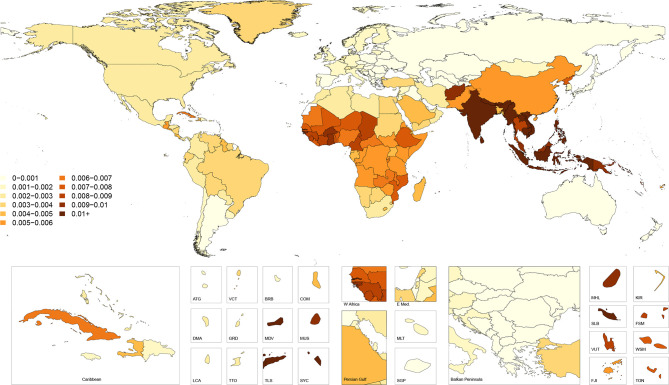
Ratio of age-standardised mortality to incidence rates, 2017, both sexes.

Figure 2 shows age-standardised cause-specific mortality rates for falls in 2017 by country. This map illustrates how the countries with the highest incidence do not necessarily have the highest cause-specific mortality, with countries such as India, Vietnam and Burkina Faso having markedly higher cause-specific mortality than the areas of Eastern and Central Europe that had the highest incidence. These patterns are further revealed in figure 3, which shows country-specific ratios of age-standardised mortality rates to age-standardised incidence rates in 2017, approximating the risk of death given a fall. This figure shows how mortality-to-incidence ratios (MIR) vary across the world. The ratio is highest in countries in Southeast Asia such as Indonesia, Cambodia, Myanmar and Vietnam, which have MIRs exceeding 0.03, meaning on average more than three deaths occur per 100 falls. MIRs also appear high throughout much of sub-Saharan Africa, in Afghanistan and across India.

Online supplementary appendix table 2 shows the all-ages deaths and the age-standardised mortality rates for 2017 as well as the percentage change in age-standardised rates from 1990 to 2017. Globally, the age-standardised mortality rate was 9.2 (8.5–9.8) per 100 000 which equated to 695 771 (644 927–741 720) deaths in 2017 and represented a non-significant decrease of 5.9% (−13.7 to 3.5) in age-standardised mortality from 1990 to 2017. Across SDI quintiles, only the high SDI quintile experienced a significant decrease in age-standardised mortality rate with a decline of 16.6% (18.8 to 14.4) from 1990 to 2017. All other quintiles experienced a non-significant decline in age-standardised mortality rates. The regions with the highest age-standardised mortality rates were South Asia with 22.0 (20.0–25.0) deaths per 100 000, Eastern Sub-Saharan Africa with 12.2 (11.2–13.5) deaths per 100 000 and Southeast Asia with 10.5 (9.8–11.3) deaths per 100 000. South Asia had the highest number of deaths, with 239 791 (220 244–270 634) deaths estimated in 2017.

10.1136/injuryprev-2019-043286.supp4 Supplementary data

### YLDs, YLLs and DALYs

Online supplementary appendix table 3 shows the counts, age-standardised rates and per cent change from 1990 to 2017 of YLDs, YLLs and DALYs. Globally, falls resulted in 16 688 088 (15 101 897–17 636 830) YLLs, 19 252 699 (13 725 429–26 140 433) YLDs and 35 940 787 (30 185 695–42 903 289) DALYs, reflecting age-standardised rates of 217 (196–229) per 100 000, 243 (173–330) per 100 000 and 459 (387–547) per 100 000, respectively. Age-standardised YLLs, YLDs and DALYs declined by 18.5% (31.7 to 6.2), 9.3% (10.7 to 7.9) and 13.9% (21.3 to 8.0), respectively, between 1990 and 2017. The percentage of age-standardised DALYs caused by YLDs varied by region, with a high of 89% in Australasia and a low of 16% in Southeast Asia. The region with the highest age-standardised DALY rate was Central Europe with 1174 (875–1559) DALYs per 100 000 which represented 159 (153–165) YLLs per 100 000 and 1015 (713–1405) YLDs per 100 000.

10.1136/injuryprev-2019-043286.supp5 Supplementary data

### Nature of injuries caused by falls

**Figure 4:**
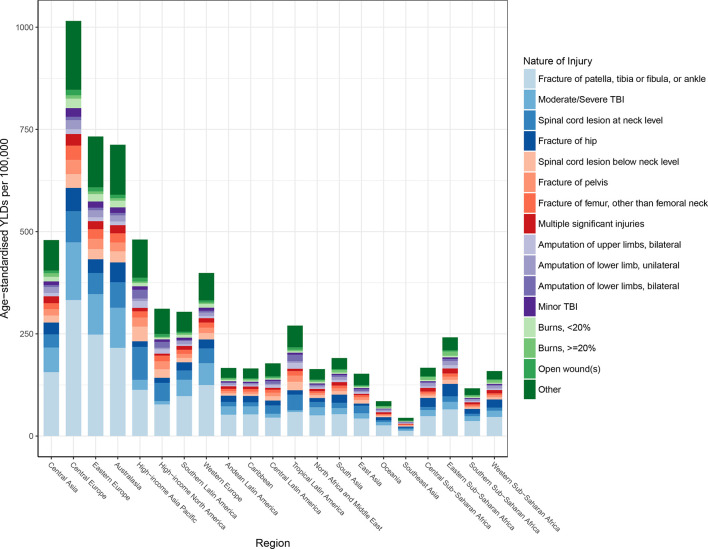
Age-standardised nature-of-injury composition of falls by region. TBI, traumatic brain injury; YLDs, years lived with disability.

**Figure 5:**
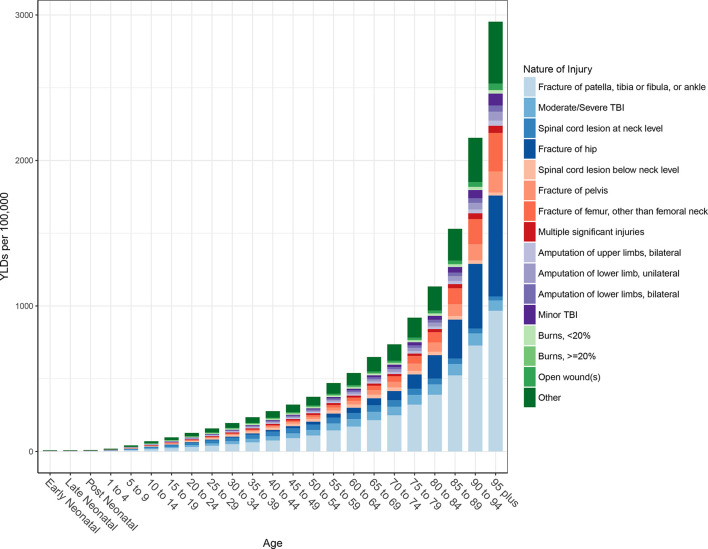
Age-specific nature-of-injury composition of falls globally. TBI, traumatic brain injury; YLDs, years lived with disability.

Globally, the average disability weight used in computing YLDs after comorbidity adjustment was 4%, meaning that the average person suffering from a fall lost 4% of their full health status. Figure 4 shows the distribution of nature-of-injury codes among all falls for age-standardised YLDs by region. This figure shows that for all 21 of the GBD regions, the leading cause of disability among fall victims is fracture of patella, tibia or fibula, or ankle. Fracture of hip and moderate/severe traumatic brain injury are the next leading causes of disability among fall victims across regions. Global age-specific distributions of nature-of-injury codes are shown in figure 5. This figure shows that fractures of patella, tibia or fibula, or ankle are the most common causes of disability after an injurious fall in all age groups, though fracture of hip and femur fracture increasingly contribute to disability in older age groups.

## Discussion

This study represents the first time that GBD estimates for falls have been reported in this level of detail through recent years, and illustrates the substantial amount of mortality and health loss in every country, age group and sex. Globally, total deaths and DALYs due to falls have increased steadily since 1990, with death counts nearly doubling by 2017. Conversely, age-standardised mortality rates and DALY rates have slightly decreased over the same period. At the country level, age-standardised mortality due to falls was highest in the Solomon Islands, India and Vietnam. The patterns of MIRs described in the results of our study emphasise how mortality risk per fall varies substantially by country and reveal that certain areas of the world likely have inadequate capabilities of responding to injurious falls. Since mortality from falls is associated with age and since global populations are generally ageing, it is important for all countries to ensure that their older adult populations as well as their ageing populations have adequate access to caretaking and treatment resources now and in the future.^10^ More focused research in the countries with the highest MIRs should investigate the specific causes of injury deaths from falls, the associated risk factors, and the circumstances and context of falls in order to target prevention efforts and appropriately allocate treatment resources. We additionally describe how falls have improved in terms of incidence and cause-specific mortality in the highest SDI countries, but that these improvements have not necessarily been experienced in lower SDI countries. This pattern emphasises how it is critical for lower SDI countries to more thoroughly investigate patterns of falls and to invest in prevention and treatment programmes.

Among clinicians, falls are known to be an important risk in certain populations, as they can be an origin of injury that leads to more complex care, such as the otherwise healthy older adult who slips, falls, sustains a femur fracture and then is admitted to the hospital for surgical repair and develops a condition like healthcare-acquired pneumonia. Such vignettes emphasise how a fall can precipitate significant health loss and potentially death. 29However, a young person who falls can also suffer disability the rest of his or her life, leading to income loss, dependence on caretakers and adequate accessibility options. Among the countries with highest incidence in 2017 were Slovenia, Czech Republic and Slovakia—countries with high percentages of rural populations.^30^ In Slovenia, nearly half of the population lives in a rural area, and there is evidence that falls are less fatal and more frequent in rural older people.^31 32^ Age-standardised DALY rates were particularly high in specific regions, including Central Europe, Eastern Europe and Australasia. Many of these regions are experiencing intensive ageing of the population.^33^ Poland, for example, is projected to increase the population aged 65 and over by 4.9 million in the years 2015–2050, requiring significant public healthcare expenditure on therapeutic rehabilitation.^34^

Research suggests that falls can cause physical harm and psychological and financial harm. A 3-year longitudinal study conducted by Tinetti and Williams explored the short and long-term effects of a fall on the well-being of those 65 and older. Among the participants, injurious falls resulted in a variety of conditions, including hip fractures, other fractures and soft tissue injuries; ultimately these injurious falls led to a decline in daily functional status.^35^ Other research has shown that falling often triggers a fear of falling again, likely impairing one’s sense of mobility and autonomy.^9^ This fear is a proven risk factor for future falls; thus, one fall can initiate a cascade of negative health outcomes.^9^ Ultimately, the initial morbidity of a fall can manifest into significant health loss over time, amounting to considerable treatment and care costs.^36^ Future GBD research may provide estimates on the probability of long-term disability for individuals who sustain injurious falls.

In general, research on the prevention of falls has shown that improving personal health as well as addressing unsafe external factors can be effective in preventing falls. For example, exercise programmes have been shown to reduce falls among community-dwelling individuals aged 65 and older.^8 37^ A person’s surrounding environment has also been identified as a leading cause of falls,^9 10^ meaning it is possible to prevent falls through the improvement of living conditions and public spaces, especially if older adults and universal design principles attending to safety are kept in mind when spaces are designed, altered and maintained.^38^ While some external hazards for falls are well known (eg, slippery surfaces or poor lighting), others are less visible or obvious. For example, in the inpatient setting, a study by Vassallo *et al* found that the hospital wards with more inpatient beds within the sightline of the nursing station had fewer falls than the ward with poor visibility between beds and the nursing station.^39^ Location-specific research in falls prevention has also shown that exercise, home modification, educational materials and vision correction are all important.^40 41^ It is also important to consider how morbidity or mortality resulting from falls might be mitigated. Clinical literature has supported frequent medication review with avoidance of polypharmacy,^42^ and dietary supplementation with cholecalciferol (vitamin D_3_) for select patients as methods to both prevent fall incidents and to help minimise fracture risk, though more recent assessments and recommendations by the US Preventive Services Task Force have revealed mixed results in terms of the benefits of vitamin D supplementation.^43–46^

Our study has several limitations. The first limitation is a function of our case definition in non-fatal models, where we estimate the incidence of falls that require medical care. While not every fall leads to injury, it is possible that care-seeking behavior with similar injuries could vary by location. Similarly, it is possible that in survey data or routine outpatient care visits, a patient may not report falls in the past year even if they led to minor injuries. Since our case definition includes only falls that lead to injury, our MIR estimates are likely lower than if we included all falls regardless of whether they led to injury requiring medical care. However, since the purpose of estimating those ratios is to illustrate patterns in severity and access to treatment, this limitation does not impact the key themes highlighted in our study. In addition, a general limitation in GBD analysis is that some areas of the world that may have high burden of various diseases and injuries do not have reliable incidence and cause-of-death data, and therefore our estimation process relies more heavily on covariates and regional trends in those areas. Similarly, the nature-of-injury distributions and injury duration parameters rely more heavily on data from higher income locations and Dutch injury data, and therefore may benefit in the future from adding more data sources from lower income locations so that that these parameters can be refined with greater location heterogeneity in future studies. Accordingly, an emphasis of GBD estimation going forward is to continue seeking additional data sources to be used in our modelling process.

## Conclusion

As reported in prior GBD literature, falls have persisted over the past three decades as a leading cause of morbidity and mortality globally. This study, which examines the burden of falls in more detail in terms of location and age-specific patterns, reveals that falls are concentrated in certain locations, but the burden of fall mortality reliably corresponds with burden of fall incidence. In other words, it appears that morbidity and mortality of falls are influenced by geographic factors that likely pertain to care access and fall severity. Further research should be conducted to better define and measure these relationships so that future policy and investment can be appropriately designed and implemented.

**What is already known on the subject**

- Prior research has shown that every region of the world experiences health loss from falls.
- Falls have consistently been a leading cause of fatal and non-fatal health loss in the Global Burden of Disease Study (GBD).

**What this study adds**

- While age-standardised incidence of injuries from falls decreased by 8.8% in the high socio-demographic index (SDI) quintile from 1990 to 2017, incidence increased in the middle, low-middle and low SDI quintiles during that time.
- Countries with the highest incidence of injuries from falls do not necessarily have the highest cause-specific mortality.
- For all 21 GBD regions, the most common nature of injury sustained by fall victims is fracture of patella, tibia or fibula, or ankle.
