# Supplementary material for: The global burden of falls: global, regional and national estimates of morbidity and mortality from the Global Burden of Disease Study 2017
Source: Inj Prev. 2020 Jan 15;26(Suppl 1):i3–i11. doi: 10.1136/injuryprev-2019-043286 (PMC7571347; doi:10.1136/injuryprev-2019-043286)
Supplement: Supplementary data [file injuryprev-2019-043286supp003.pdf]

**Table 1: Incidence and prevalence for 2017 and percentage change of age-standardised rates between 1990 and 2017 by location for falls**

| Location | Incidence (95% UI) | | | Prevalence (95% UI) | | |
|---|---|---|---|---|---|---|
| | 2017 counts | 2017 age-standardised rates per 100,000 | Percentage change in age-standardised rates between 1990 and 2017 | 2017 counts | 2017 age-standardised rates per 100,000 | Percentage change in age-standardised rates between 1990 and 2017 |
| **Global** | **171 691 220<br>(152 472 652 to 194 061 874)** | **2 238<br>(1 990 to 2 532)** | **-3.7<br>(-7.4 to -0.3)** | **411 711 999<br>(366 390 987 to 465 354 952)** | **5 186<br>(4 622 to 5 849)** | **-6.5<br>(-7.6 to -5.4)** |
| **Low SDI** | **17 499 653<br>(14 975 887 to 20 547 072)** | **1 597<br>(1 400 to 1 824)** | **16.0<br>(11.3 to 20.6)** | **31 022 660<br>(27 331 173 to 35 024 800)** | **3 393<br>(3 010 to 3 836)** | **15.3<br>(13.7 to 17.0)** |
| **Low-middle SDI** | **24 401 866<br>(21 203 737 to 28 302 838)** | **1 581<br>(1 390 to 1 805)** | **26.0<br>(21.2 to 30.6)** | **47 383 747<br>(41 829 874 to 53 437 565)** | **3 379<br>(3 010 to 3 807)** | **23.6<br>(21.9 to 25.6)** |
| **Middle SDI** | **28 246 597<br>(24 926 685 to 32 295 306)** | **1 353<br>(1 195 to 1 544)** | **41.1<br>(36.3 to 45.7)** | **65 634 605<br>(58 297 959 to 74 204 118)** | **2 956<br>(2 634 to 3 335)** | **38.8<br>(36.1 to 41.5)** |
| **High-middle SDI** | **43 941 932<br>(38 932 641 to 49 520 075)** | **3 133<br>(2 758 to 3 570)** | **-4.0<br>(-7.9 to -0.2)** | **116 716 021<br>(102 845 012 to 132 955 230)** | **7 029<br>(6 191 to 7 997)** | **-10.1<br>(-11.5 to -8.7)** |
| **High SDI** | **57 175 600<br>(51 319 808 to 63 910 608)** | **4 307<br>(3 770 to 4 929)** | **-8.8<br>(-12.3 to -5.3)** | **149 790 629<br>(133 521 836 to 170 195 274)** | **9 316<br>(8 135 to 10 647)** | **-7.6<br>(-9.0 to -6.1)** |
| **Central Europe, Eastern Europe, and Central Asia** | **36 244 565<br>(32 310 224 to 40 669 642)** | **8 240<br>(7 262 to 9 388)** | **-4.1<br>(-8.4 to 0.1)** | **93 942 180<br>(82 524 124 to 108 293 264)** | **17 959<br>(15 660 to 20 662)** | **-2.5<br>(-3.6 to -1.2)** |
| **Central Asia** | **4 253 163<br>(3 692 253 to 4 883 974)** | **4 761<br>(4 146 to 5 475)** | **-4.2<br>(-10.6 to 1.9)** | **9 249 220<br>(7 998 797 to 10 645 734)** | **10 653<br>(9 274 to 12 223)** | **-5.4<br>(-6.8 to -3.9)** |
| Armenia | 141 308<br>(122 550 to 162 828) | 4 670<br>(3 988 to 5 473) | -18.4<br>(-25.7 to -11.1) | 378 950<br>(329 119 to 438 804) | 10 471<br>(9 051 to 12 106) | -17.9<br>(-19.9 to -15.6) |
| Azerbaijan | 476 025<br>(407 964 to 551 195) | 4 705<br>(4 051 to 5 457) | 5.6<br>(-2.6 to 14.4) | 1 116 858<br>(961 605 to 1 296 298) | 10 544<br>(9 131 to 12 206) | 4.1<br>(1.8 to 6.4) |
| Georgia | 184 261<br>(164 254 to 208 007) | 4 668<br>(4 137 to 5 332) | -15.4<br>(-22.0 to -7.8) | 473 778<br>(419 041 to 541 441) | 9 921<br>(8 726 to 11 347) | -18.7<br>(-20.4 to -16.9) |
| Kazakhstan | 937 158<br>(826 507 to 1 067 295) | 5 306<br>(4 690 to 6 076) | 0.2<br>(-7.3 to 8.1) | 2 077 867<br>(1 807 985 to 2 394 836) | 11 489<br>(10 029 to 13 190) | -1.8<br>(-3.3 to -0.1) |
| Kyrgyzstan | 263 893<br>(222 094 to 311 221) | 4 188<br>(3 571 to 4 890) | -25.1<br>(-32.7 to -16.8) | 548 570<br>(470 213 to 635 527) | 9 708<br>(8 370 to 11 198) | -22.5<br>(-24.9 to -20.2) |
| Mongolia | 189 074<br>(166 033 to 216 155) | 5 915<br>(5 223 to 6 747) | 11.8<br>(5.8 to 17.9) | 395 668<br>(342 567 to 456 194) | 13 234<br>(11 568 to 15 192) | 10.7<br>(8.6 to 13.2) |
| Tajikistan | 422 650<br>(365 070 to 497 518) | 4 660<br>(4 082 to 5 425) | -11.6<br>(-17.4 to -4.9) | 811 897<br>(700 577 to 937 562) | 10 868<br>(9 476 to 12 448) | -10.9<br>(-12.7 to -9.2) |
| Turkmenistan | 223 612<br>(190 766 to 260 932) | 4 586<br>(3 934 to 5 352) | 4.6<br>(-3.9 to 12.2) | 485 983<br>(417 368 to 564 662) | 10 396<br>(8 996 to 12 001) | 3.1<br>(1.0 to 5.1) |
| Uzbekistan | 1 415 182<br>(1 200 497 to 1 651 819) | 4 451<br>(3 816 to 5 176) | 0.5<br>(-8.0 to 8.4) | 2 959 650<br>(2 547 463 to 3 438 521) | 10 088<br>(8 752 to 11 624) | -0.8<br>(-2.7 to 1.5) |
| **Central Europe** | **14 767 638<br>(13 282 613 to 16 427 055)** | **11 434<br>(10 103 to 12 996)** | **-5.9<br>(-11.2 to -0.8)** | **37 233 284<br>(33 073 677 to 42 407 489)** | **23 428<br>(20 453 to 26 911)** | **-3.9<br>(-5.4 to -2.2)** |
| Albania | 267 827<br>(234 155 to 305 388) | 9 528<br>(8 188 to 11 053) | 30.2<br>(23.9 to 36.7) | 714 463<br>(620 728 to 827 904) | 20 964<br>(18 128 to 24 261) | 22.2<br>(19.8 to 24.6) |
| Bosnia and Herzegovina | 325 989<br>(285 952 to 372 233) | 9 556<br>(8 216 to 11 168) | 29.3<br>(23.9 to 34.7) | 960 384<br>(835 505 to 1 112 321) | 21 321<br>(18 395 to 24 752) | 22.8<br>(20.6 to 25.3) |
| Bulgaria | 782 650<br>(701 965 to 878 893) | 10 252<br>(8 954 to 11 755) | -4.8<br>(-11.5 to 2.5) | 2 241 967<br>(1 975 487 to 2 580 312) | 21 980<br>(19 035 to 25 361) | -7.7<br>(-9.0 to -5.9) |
| Croatia | 532 044<br>(498 864 to 569 372) | 9 356<br>(8 814 to 10 029) | -3.0<br>(-11.7 to 6.7) | 1 140 021<br>(1 036 701 to 1 269 180) | 17 368<br>(15 655 to 19 446) | -7.3<br>(-10.6 to -4.3) |
| Czech Republic | 1 601 099<br>(1 419 768 to 1 809 994) | 13 389<br>(11 519 to 15 469) | -11.9<br>(-19.7 to -4.6) | 4 018 820<br>(3 557 410 to 4 581 118) | 27 302<br>(23 797 to 31 517) | 2.1<br>(-0.3 to 5.1) |
| Hungary | 1 377 645<br>(1 226 330 to 1 542 949) | 11 783<br>(10 312 to 13 538) | -23.0<br>(-29.7 to -16.2) | 3 260 122<br>(2 897 693 to 3 720 297) | 23 372<br>(20 420 to 26 906) | -9.8<br>(-12.9 to -6.4) |
| Macedonia | 217 944<br>(191 778 to 246 425) | 9 873<br>(8 563 to 11 447) | 29.3<br>(23.9 to 35.0) | 593 523<br>(516 649 to 687 735) | 21 535<br>(18 620 to 24 957) | 23.0<br>(21.1 to 25.0) |
| Montenegro | 63 348<br>(55 787 to 71 924) | 9 879<br>(8 484 to 11 465) | 17.8<br>(12.3 to 23.9) | 170 139<br>(147 924 to 197 611) | 21 556<br>(18 574 to 25 063) | 17.3<br>(15.4 to 19.0) |
| Poland | 5 206 349<br>(4 680 507 to 5 801 280) | 11 933<br>(10 506 to 13 597) | -0.6<br>(-7.5 to 6.8) | 12 756 978<br>(11 331 892 to 14 475 960) | 24 118<br>(21 141 to 27 664) | -0.3<br>(-2.1 to 1.3) |
| Romania | 2 335 397<br>(2 096 120 to 2 593 540) | 10 889<br>(9 551 to 12 429) | -16.8<br>(-23.1 to -9.6) | 6 231 936<br>(5 525 360 to 7 109 467) | 22 887<br>(19 963 to 26 243) | -20.2<br>(-22.1 to -18.2) |
| Serbia | 935 472<br>(828 128 to 1 057 295) | 10 013<br>(8 637 to 11 598) | 22.9<br>(16.7 to 28.8) | 2 524 792<br>(2 212 807 to 2 919 389) | 21 561<br>(18 658 to 24 978) | 20.4<br>(18.1 to 22.9) |
| Slovakia | 738 806<br>(661 530 to 826 576) | 12 239<br>(10 806 to 13 916) | -11.2<br>(-16.9 to -5.5) | 1 774 767<br>(1 578 271 to 2 018 410) | 24 425<br>(21 482 to 27 965) | -8.4<br>(-10.4 to -6.2) |
| Slovenia | 383 068<br>(340 080 to 432 286) | 14 790<br>(12 950 to 16 774) | -1.0<br>(-8.9 to 7.1) | 845 372<br>(763 593 to 942 614) | 28 254<br>(24 953 to 32 226) | 4.6<br>(2.6 to 6.6) |
| **Eastern Europe** | **17 223 763<br>(15 097 423 to 19 546 254)** | **8 029<br>(7 010 to 9 233)** | **3.6<br>(-1.1 to 8.6)** | **47 459 675<br>(41 508 361 to 55 020 220)** | **17 429<br>(15 114 to 20 228)** | **3.2<br>(1.7 to 4.7)** |
| Belarus | 836 348<br>(752 621 to 931 024) | 8 433<br>(7 448 to 9 491) | 2.8<br>(-3.9 to 9.9) | 2 259 393<br>(1 990 759 to 2 598 887) | 17 965<br>(15 690 to 20 674) | -0.1<br>(-1.7 to 1.7) |
| Estonia | 112 476<br>(100 762 to 126 160) | 7 995<br>(6 982 to 9 154) | -21.9<br>(-28.7 to -14.5) | 313 546<br>(276 093 to 361 654) | 17 231<br>(14 939 to 19 945) | -18.2<br>(-20.6 to -15.7) |
| Latvia | 181 592<br>(162 291 to 201 400) | 8 437<br>(7 417 to 9 528) | -22.7<br>(-29.1 to -15.5) | 492 351<br>(436 833 to 564 524) | 17 725<br>(15 518 to 20 317) | -19.8<br>(-22.5 to -17.3) |
| Lithuania | 304 680<br>(274 072 to 337 700) | 9 236<br>(8 175 to 10 379) | -6.8<br>(-13.8 to 0.9) | 785 538<br>(699 256 to 896 628) | 18 919<br>(16 631 to 21 733) | -8.0<br>(-10.7 to -5.4) |
| Moldova | 246 915<br>(218 517 to 279 575) | 6 749<br>(5 856 to 7 797) | -16.7<br>(-23.8 to -9.0) | 710 311<br>(620 107 to 822 168) | 15 209<br>(13 194 to 17 556) | -14.4<br>(-16.7 to -11.6) |
| Russian Federation | 12 075 603<br>(10 555 676 to 13 773 029) | 8 082<br>(7 057 to 9 303) | 9.3<br>(4.4 to 14.2) | 32 823 200<br>(28 687 609 to 38 056 625) | 17 542<br>(15 203 to 20 362) | 9.4<br>(7.8 to 11.0) |
| Ukraine | 3 466 149<br>(2 998 287 to 3 979 301) | 7 785<br>(6 725 to 9 006) | -6.4<br>(-11.7 to -0.9) | 10 075 335<br>(8 786 177 to 11 720 903) | 17 029<br>(14 737 to 19 813) | -7.2<br>(-8.7 to -5.5) |
| **High-income** | **49 103 178<br>(43 975 014 to 54 930 253)** | **3 900<br>(3 415 to 4 469)** | **-8.5<br>(-11.9 to -5.0)** | **128 475 940<br>(114 333 653 to 146 380 526)** | **8 516<br>(7 433 to 9 743)** | **-8.4<br>(-10.2 to -6.6)** |
| **Australasia** | **2 680 001<br>(2 368 150 to 3 030 484)** | **8 187<br>(6 978 to 9 553)** | **25.5<br>(18.8 to 32.7)** | **5 709 338<br>(4 924 777 to 6 795 061)** | **16 175<br>(13 641 to 19 647)** | **19.7<br>(16.9 to 22.3)** |
| Australia | 2 159 427<br>(1 892 073 to 2 460 772) | 7 888<br>(6 638 to 9 269) | 26.0<br>(18.6 to 34.0) | 4 702 711<br>(4 049 255 to 5 591 701) | 15 785<br>(13 277 to 19 200) | 21.0<br>(19.0 to 23.4) |
| New Zealand | 520 575<br>(462 937 to 584 568) | 9 799<br>(8 577 to 11 062) | 25.9<br>(16.6 to 35.6) | 1 006 627<br>(874 748 to 1 186 462) | 18 269<br>(15 521 to 22 017) | 15.1<br>(8.3 to 21.6) |
| **High-income Asia-Pacific** | **8 907 346<br>(7 810 673 to 10 058 576)** | **4 450<br>(3 810 to 5 197)** | **7.5<br>(2.2 to 12.6)** | **28 152 727<br>(24 660 093 to 32 450 922)** | **10 300<br>(8 816 to 12 086)** | **12.3<br>(10.3 to 14.8)** |
| Brunei | 20 115<br>(17 507 to 22 960) | 5 065<br>(4 413 to 5 814) | 7.5<br>(1.6 to 12.8) | 46 270<br>(39 697 to 54 145) | 11 009<br>(9 552 to 12 820) | 6.2<br>(4.4 to 8.1) |
| Japan | 6 424 809<br>(5 588 243 to 7 321 969) | 4 501<br>(3 833 to 5 277) | 19.8<br>(15.4 to 24.4) | 20 945 728<br>(18 408 357 to 24 193 741) | 10 612<br>(9 060 to 12 470) | 25.6<br>(22.9 to 29.0) |
| South Korea | 2 238 628<br>(1 985 828 to 2 508 127) | 4 423<br>(3 807 to 5 103) | -10.0<br>(-17.8 to -1.5) | 6 508 161<br>(5 674 209 to 7 516 082) | 9 628<br>(8 289 to 11 311) | -14.3<br>(-16.3 to -12.1) |
| Singapore | 223 793<br>(195 607 to 255 803) | 4 401<br>(3 771 to 5 155) | 5.8<br>(-0.1 to 11.8) | 652 569<br>(558 380 to 759 874) | 10 048<br>(8 595 to 11 773) | 5.8<br>(3.1 to 8.2) |
| **High-income North America** | **13 844 032<br>(12 097 284 to 15 788 708)** | **3 135<br>(2 751 to 3 565)** | **-25.1<br>(-31.3 to -19.1)** | **33 970 923<br>(29 874 085 to 39 104 635)** | **6 653<br>(5 843 to 7 600)** | **-29.8<br>(-35.2 to -24.4)** |
| Canada | 1 465 341<br>(1 307 300 to 1 647 918) | 3 277<br>(2 892 to 3 738) | 8.2<br>(2.5 to 13.7) | 3 462 624<br>(3 094 832 to 3 919 349) | 6 663<br>(5 909 to 7 547) | 6.7<br>(5.2 to 8.4) |
| Greenland | 2 551<br>(2 274 to 2 851) | 4 297<br>(3 841 to 4 806) | -24.6<br>(-29.1 to -20.2) | 5 505<br>(4 888 to 6 232) | 8 550<br>(7 623 to 9 649) | -28.9<br>(-30.7 to -27.2) |
| USA | 12 375 898<br>(10 788 202 to 14 145 067) | 3 122<br>(2 734 to 3 561) | -27.4<br>(-34.0 to -21.2) | 30 502 200<br>(26 747 366 to 35 199 706) | 6 658<br>(5 835 to 7 611) | -32.3<br>(-37.9 to -26.6) |
| **Southern Latin America** | **2 187 890<br>(1 989 360 to 2 427 548)** | **3 250<br>(2 925 to 3 653)** | **-3.8<br>(-8.0 to 0.3)** | **5 005 794<br>(4 420 487 to 5 653 636)** | **6 798<br>(5 978 to 7 711)** | **-7.5<br>(-8.8 to -6.3)** |
| Argentina | 1 457 216<br>(1 280 419 to 1 662 038) | 3 239<br>(2 816 to 3 743) | -1.9<br>(-7.7 to 3.5) | 3 379 374<br>(2 953 650 to 3 846 931) | 6 972<br>(6 067 to 7 960) | -3.7<br>(-5.8 to -1.8) |
| Chile | 601 770<br>(577 618 to 633 697) | 3 223<br>(3 092 to 3 398) | -10.5<br>(-18.5 to -1.7) | 1 325 549<br>(1 199 546 to 1 478 324) | 6 340<br>(5 705 to 7 107) | -18.5<br>(-21.0 to -15.7) |

| Location | Incidence (95% UI) | | | Prevalence (95% UI) | | |
|---|---|---|---|---|---|---|
| | 2017 counts | 2017 age-standardised rates per 100,000 | Percentage change in age-standardised rates between 1990 and 2017 | 2017 counts | 2017 age-standardised rates per 100,000 | Percentage change in age-standardised rates between 1990 and 2017 |
| Uruguay | 128 808 (114 320 to 145 427) | 3 369 (2 944 to 3 838) | 3.5 (-1.8 to 9.5) | 300 649 (267 360 to 340 315) | 7 004 (6 155 to 7 964) | 0.7 (-0.9 to 2.4) |
| **Western Europe** | **21 483 909 (19 383 395 to 24 007 380)** | **4 267 (3 704 to 4 935)** | **-2.3 (-6.1 to 1.5)** | **55 637 159 (49 150 798 to 63 388 923)** | **9 142 (7 914 to 10 580)** | **-1.0 (-1.7 to -0.3)** |
| Andorra | 3 866 (3 443 to 4 350) | 4 572 (3 965 to 5 256) | 1.0 (-4.2 to 6.3) | 10 590 (9 305 to 12 081) | 9 820 (8 507 to 11 333) | 0.7 (-0.8 to 2.3) |
| Austria | 461 202 (415 458 to 514 214) | 4 583 (3 996 to 5 298) | -17.1 (-22.9 to -11.2) | 1 202 772 (1 064 397 to 1 370 161) | 9 745 (8 420 to 11 288) | -14.2 (-16.0 to -12.5) |
| Belgium | 738 406 (660 137 to 830 740) | 5 378 (4 689 to 6 166) | 14.9 (8.5 to 22.1) | 1 784 786 (1 583 131 to 2 027 425) | 11 191 (9 723 to 12 923) | 12.7 (11.1 to 14.5) |
| Cyprus | 51 237 (45 147 to 58 060) | 4 176 (3 594 to 4 862) | -0.3 (-5.0 to 5.1) | 142 633 (123 796 to 163 843) | 9 203 (7 900 to 10 664) | 0.5 (-1.1 to 2.0) |
| Denmark | 275 778 (243 153 to 312 177) | 4 346 (3 729 to 5 072) | -8.0 (-14.3 to -1.4) | 714 858 (625 618 to 819 450) | 9 312 (7 996 to 10 829) | 0.8 (-1.2 to 2.7) |
| Finland | 369 102 (331 439 to 415 951) | 5 435 (4 769 to 6 269) | 5.4 (-0.8 to 12.7) | 903 704 (805 416 to 1 030 509) | 11 297 (9 869 to 13 082) | 7.9 (6.3 to 9.4) |
| France | 3 776 416 (3 382 461 to 4 212 943) | 4 619 (4 013 to 5 327) | -8.4 (-13.3 to -2.8) | 9 019 607 (8 009 442 to 10 268 965) | 9 718 (8 426 to 11 223) | -4.9 (-6.3 to -3.5) |
| Germany | 4 435 165 (3 996 472 to 4 949 163) | 4 447 (3 852 to 5 147) | -0.3 (-6.3 to 5.8) | 11 574 449 (10 236 811 to 13 190 387) | 9 468 (8 195 to 11 013) | 1.2 (-0.2 to 2.9) |
| Greece | 416 500 (372 994 to 465 633) | 3 861 (3 281 to 4 522) | -6.8 (-12.1 to -2.2) | 1 284 041 (1 126 871 to 1 470 521) | 8 752 (7 510 to 10 155) | -5.3 (-6.9 to -3.8) |
| Iceland | 15 625 (13 869 to 17 665) | 4 378 (3 786 to 5 075) | 3.3 (-1.7 to 8.9) | 39 418 (34 439 to 45 300) | 9 493 (8 185 to 11 006) | 3.5 (2.0 to 5.3) |
| Ireland | 207 341 (183 484 to 235 879) | 4 231 (3 656 to 4 928) | 2.8 (-2.0 to 7.5) | 539 897 (470 349 to 623 617) | 9 322 (8 053 to 10 887) | 4.1 (2.1 to 6.1) |
| Israel | 368 373 (316 292 to 430 370) | 4 015 (3 419 to 4 710) | 10.1 (4.2 to 15.8) | 865 519 (744 674 to 1 003 947) | 9 048 (7 736 to 10 554) | 10.8 (8.0 to 13.1) |
| Italy | 2 841 180 (2 553 045 to 3 170 734) | 3 802 (3 331 to 4 382) | -10.4 (-15.5 to -5.3) | 7 461 482 (6 645 747 to 8 466 579) | 8 111 (7 054 to 9 331) | -9.2 (-11.0 to -7.4) |
| Luxembourg | 30 368 (27 401 to 34 096) | 4 700 (4 101 to 5 407) | -8.9 (-14.6 to -2.7) | 75 168 (66 190 to 85 833) | 9 811 (8 518 to 11 307) | -9.2 (-10.7 to -7.6) |
| Malta | 23 160 (20 873 to 25 814) | 5 007 (4 347 to 5 720) | 12.1 (6.0 to 18.4) | 63 947 (56 382 to 73 093) | 10 794 (9 349 to 12 567) | 12.3 (9.9 to 14.3) |
| Netherlands | 721 872 (659 797 to 793 950) | 3 676 (3 323 to 4 125) | 4.1 (-4.2 to 13.2) | 1 765 054 (1 573 989 to 2 000 060) | 7 565 (6 616 to 8 739) | 1.5 (-1.3 to 4.1) |
| Norway | 299 873 (259 974 to 339 786) | 4 995 (4 301 to 5 752) | -1.6 (-5.3 to 1.7) | 749 990 (659 212 to 859 692) | 10 815 (9 321 to 12 589) | -0.9 (-2.2 to 0.1) |
| Portugal | 371 169 (330 725 to 415 610) | 3 112 (2 695 to 3 627) | -27.4 (-33.0 to -21.1) | 1 054 998 (934 591 to 1 201 872) | 6 810 (5 909 to 7 857) | -29.7 (-31.5 to -27.7) |
| Spain | 1 922 262 (1 716 547 to 2 154 504) | 3 826 (3 281 to 4 475) | -1.1 (-6.2 to 4.5) | 5 498 483 (4 826 112 to 6 271 470) | 8 497 (7 306 to 9 828) | -1.1 (-2.6 to 0.3) |
| Sweden | 539 643 (466 549 to 614 803) | 4 634 (3 976 to 5 367) | 11.7 (7.6 to 15.9) | 1 432 049 (1 256 323 to 1 647 133) | 10 367 (8 882 to 12 094) | 11.9 (10.1 to 13.9) |
| Switzerland | 497 728 (458 772 to 547 216) | 4 594 (4 167 to 5 142) | -26.6 (-31.8 to -20.1) | 1 154 454 (1 035 156 to 1 300 314) | 9 217 (8 117 to 10 476) | -27.3 (-29.2 to -25.2) |
| United Kingdom | 3 095 384 (2 704 973 to 3 500 866) | 4 290 (3 678 to 4 985) | 14.4 (10.8 to 17.9) | 8 241 612 (7 220 907 to 9 439 086) | 9 419 (8 109 to 10 926) | 15.2 (13.8 to 16.9) |
| **Latin America and Caribbean** | **11 776 802 (10 571 218 to 13 159 361)** | **2 059 (1 843 to 2 297)** | **29.6 (26.4 to 32.8)** | **25 073 723 (22 386 743 to 28 287 435)** | **4 286 (3 837 to 4 840)** | **29.5 (27.5 to 31.9)** |
| **Andean Latin America** | **975 728 (855 995 to 1 111 401)** | **1 642 (1 446 to 1 868)** | **25.7 (19.0 to 31.7)** | **1 984 980 (1 770 537 to 2 236 342)** | **3 444 (3 083 to 3 878)** | **23.2 (20.8 to 26.5)** |
| Bolivia | 167 244 (145 638 to 192 298) | 1 588 (1 402 to 1 799) | 16.6 (9.4 to 23.9) | 324 413 (288 265 to 364 319) | 3 304 (2 955 to 3 699) | 15.2 (12.6 to 18.3) |
| Ecuador | 302 692 (267 916 to 343 348) | 1 885 (1 674 to 2 130) | 14.1 (6.1 to 23.3) | 610 719 (548 029 to 688 230) | 3 885 (3 497 to 4 371) | 11.3 (8.9 to 14.1) |
| Peru | 505 793 (441 152 to 576 364) | 1 548 (1 351 to 1 763) | 36.3 (29.0 to 43.9) | 1 049 848 (934 637 to 1 188 225) | 3 277 (2 924 to 3 712) | 33.1 (29.7 to 37.8) |
| **Caribbean** | **823 052 (731 226 to 928 599)** | **1 716 (1 520 to 1 940)** | **30.0 (24.5 to 36.3)** | **1 707 204 (1 528 010 to 1 923 194)** | **3 441 (3 077 to 3 885)** | **30.0 (27.4 to 33.3)** |
| Antigua and Barbuda | 1 293 (1 114 to 1 493) | 1 430 (1 238 to 1 655) | 42.2 (36.2 to 48.7) | 2 985 (2 642 to 3 371) | 3 007 (2 658 to 3 400) | 39.7 (35.5 to 43.8) |
| The Bahamas | 5 595 (4 897 to 6 403) | 1 537 (1 352 to 1 757) | 30.7 (24.4 to 36.4) | 12 017 (10 665 to 13 559) | 3 099 (2 762 to 3 504) | 31.0 (27.2 to 35.2) |
| Barbados | 4 873 (4 249 to 5 634) | 1 457 (1 263 to 1 685) | 39.7 (33.8 to 46.6) | 11 917 (10 644 to 13 429) | 3 033 (2 683 to 3 417) | 38.5 (34.3 to 42.6) |
| Belize | 5 352 (4 593 to 6 193) | 1 508 (1 326 to 1 711) | 47.3 (41.2 to 53.4) | 10 442 (9 210 to 11 888) | 3 174 (2 828 to 3 572) | 46.9 (42.7 to 52.1) |
| Bermuda | 1 299 (1 140 to 1 494) | 1 582 (1 381 to 1 818) | 25.3 (19.3 to 31.4) | 3 135 (2 801 to 3 560) | 3 217 (2 868 to 3 645) | 26.4 (23.0 to 30.1) |
| Cuba | 315 328 (283 323 to 351 233) | 2 130 (1 907 to 2 402) | 27.8 (20.5 to 36.5) | 625 959 (562 627 to 704 614) | 3 886 (3 487 to 4 378) | 25.9 (22.5 to 29.3) |
| Dominica | 1 062 (935 to 1 215) | 1 435 (1 251 to 1 649) | 48.0 (42.2 to 54.7) | 2 506 (2 234 to 2 831) | 3 072 (2 729 to 3 472) | 47.1 (42.8 to 52.2) |
| Dominican Republic | 146 235 (125 939 to 168 242) | 1 453 (1 259 to 1 673) | 50.0 (43.2 to 57.1) | 311 474 (274 376 to 353 721) | 3 115 (2 754 to 3 534) | 44.4 (39.6 to 49.9) |
| Grenada | 1 881 (1 662 to 2 132) | 1 535 (1 342 to 1 749) | 37.3 (30.4 to 43.9) | 4 271 (3 822 to 4 821) | 3 219 (2 871 to 3 624) | 38.0 (33.5 to 42.3) |
| Guyana | 11 312 (9 939 to 12 961) | 1 642 (1 452 to 1 866) | 37.5 (30.1 to 44.8) | 22 743 (20 250 to 25 523) | 3 361 (3 008 to 3 766) | 36.8 (32.4 to 41.1) |
| Haiti | 128 573 (109 188 to 150 412) | 1 285 (1 119 to 1 474) | 30.2 (23.2 to 37.2) | 245 488 (215 380 to 277 676) | 2 707 (2 414 to 3 025) | 29.7 (26.2 to 33.3) |
| Jamaica | 40 860 (35 361 to 47 156) | 1 475 (1 271 to 1 700) | 46.0 (40.3 to 51.7) | 94 065 (83 351 to 106 483) | 3 219 (2 847 to 3 648) | 43.3 (38.9 to 48.6) |
| Puerto Rico | 91 170 (81 236 to 102 808) | 1 940 (1 700 to 2 212) | 35.9 (28.4 to 44.5) | 210 801 (188 473 to 238 359) | 3 842 (3 426 to 4 333) | 34.8 (31.4 to 38.7) |
| Saint Lucia | 2 562 (2 233 to 2 928) | 1 418 (1 235 to 1 625) | 38.1 (32.5 to 44.5) | 5 987 (5 332 to 6 742) | 3 009 (2 672 to 3 395) | 38.3 (34.3 to 43.0) |
| Saint Vincent and the Grenadines | 1 938 (1 718 to 2 183) | 1 610 (1 423 to 1 826) | 51.6 (44.6 to 58.1) | 4 350 (3 888 to 4 892) | 3 384 (3 016 to 3 802) | 51.1 (45.8 to 56.0) |
| Suriname | 8 965 (7 832 to 10 247) | 1 587 (1 390 to 1 806) | 42.2 (35.0 to 49.9) | 18 863 (16 795 to 21 246) | 3 231 (2 882 to 3 628) | 40.5 (36.5 to 45.4) |
| Trinidad and Tobago | 23 013 (20 156 to 26 218) | 1 569 (1 367 to 1 785) | 26.1 (19.2 to 33.6) | 54 029 (48 180 to 60 799) | 3 291 (2 933 to 3 699) | 27.6 (24.4 to 31.8) |
| Virgin Islands | 2 144 (1 889 to 2 437) | 1 690 (1 482 to 1 926) | 37.2 (31.3 to 43.6) | 4 781 (4 253 to 5 437) | 3 262 (2 908 to 3 696) | 32.6 (28.6 to 36.4) |
| **Central Latin America** | **4 365 274 (3 891 450 to 4 938 440)** | **1 810 (1 614 to 2 044)** | **10.9 (7.1 to 15.0)** | **8 704 856 (7 732 689 to 9 912 609)** | **3 576 (3 182 to 4 074)** | **9.9 (8.0 to 12.4)** |
| Colombia | 639 271 (563 674 to 733 681) | 1 261 (1 105 to 1 448) | -8.0 (-15.1 to -0.7) | 1 345 985 (1 194 928 to 1 530 954) | 2 544 (2 253 to 2 901) | -6.2 (-8.6 to -3.9) |
| Costa Rica | 73 314 (65 346 to 82 701) | 1 586 (1 406 to 1 802) | 8.6 (1.8 to 15.8) | 145 159 (129 533 to 163 783) | 2 958 (2 635 to 3 348) | 13.2 (10.7 to 16.4) |
| El Salvador | 91 859 (81 906 to 103 738) | 1 580 (1 406 to 1 789) | 24.9 (16.5 to 34.1) | 171 514 (152 981 to 194 992) | 2 902 (2 591 to 3 291) | 17.1 (14.1 to 20.1) |
| Guatemala | 208 164 (180 559 to 240 469) | 1 461 (1 292 to 1 657) | 8.2 (1.8 to 15.3) | 382 640 (335 380 to 439 929) | 2 862 (2 556 to 3 250) | 6.9 (4.5 to 10.0) |
| Honduras | 100 651 (86 007 to 118 499) | 1 193 (1 046 to 1 376) | 29.3 (22.2 to 36.4) | 190 567 (165 033 to 221 735) | 2 497 (2 195 to 2 843) | 23.2 (19.8 to 27.0) |
| Mexico | 2 697 567 (2 389 537 to 3 043 225) | 2 276 (2 016 to 2 566) | 20.2 (15.8 to 24.9) | 5 357 266 (4 779 128 to 6 112 974) | 4 482 (4 002 to 5 130) | 17.6 (15.1 to 21.1) |
| Nicaragua | 73 151 (63 266 to 85 697) | 1 300 (1 141 to 1 497) | 17.1 (10.7 to 24.2) | 137 756 (120 960 to 157 881) | 2 537 (2 251 to 2 872) | 16.6 (13.4 to 19.8) |

| Location | Incidence (95% UI) | | | Prevalence (95% UI) | | |
|---|---|---|---|---|---|---|
| | 2017 counts | 2017 age-standardised rates per 100,000 | Percentage change in age-standardised rates between 1990 and 2017 | 2017 counts | 2017 age-standardised rates per 100,000 | Percentage change in age-standardised rates between 1990 and 2017 |
| Panama | 53 215<br>(47 145 to 60 506) | 1 359<br>(1 201 to 1 549) | 13.6<br>(7.3 to 20.4) | 107 674<br>(95 861 to 121 838) | 2 706<br>(2 405 to 3 064) | 16.1<br>(13.0 to 19.4) |
| Venezuela | 428 082<br>(375 802 to 486 515) | 1 497<br>(1 318 to 1 707) | 0.5<br>(-6.1 to 7.8) | 866 296<br>(764 916 to 993 630) | 2 929<br>(2 596 to 3 336) | 2.3<br>(-0.5 to 5.3) |
| **Tropical Latin America** | **5 612 748<br>(5 035 668 to 6 288 061)** | **2 522<br>(2 266 to 2 814)** | **48.7<br>(44.6 to 52.8)** | **12 676 683<br>(11 247 927 to 14 408 324)** | **5 402<br>(4 803 to 6 117)** | **48.0<br>(44.5 to 51.8)** |
| Brazil | 5 480 139<br>(4 920 456 to 6 134 580) | 2 536<br>(2 280 to 2 826) | 49.2<br>(45.1 to 53.5) | 12 412 809<br>(11 011 375 to 14 113 681) | 5 430<br>(4 827 to 6 149) | 48.6<br>(45.0 to 52.4) |
| Paraguay | 132 608<br>(113 800 to 152 872) | 2 032<br>(1 776 to 2 311) | 28.0<br>(21.8 to 33.9) | 263 875<br>(232 752 to 298 429) | 4 287<br>(3 805 to 4 829) | 20.6<br>(17.6 to 23.8) |
| **North Africa and Middle East** | **9 084 601<br>(7 746 853 to 10 562 639)** | **1 562<br>(1 349 to 1 793)** | **3.2<br>(-1.5 to 7.5)** | **19 413 992<br>(16 874 777 to 22 281 107)** | **3 582<br>(3 144 to 4 081)** | **-0.1<br>(-1.5 to 1.6)** |
| **North Africa and Middle East** | **9 084 601<br>(7 746 853 to 10 562 639)** | **1 562<br>(1 349 to 1 793)** | **3.2<br>(-1.5 to 7.5)** | **19 413 992<br>(16 874 777 to 22 281 107)** | **3 582<br>(3 144 to 4 081)** | **-0.1<br>(-1.5 to 1.6)** |
| Afghanistan | 503 769<br>(423 506 to 596 609) | 1 528<br>(1 334 to 1 740) | -0.9<br>(-5.7 to 4.0) | 803 232<br>(687 686 to 939 871) | 3 634<br>(3 203 to 4 134) | -2.2<br>(-4.4 to 0.3) |
| Algeria | 591 550<br>(508 284 to 679 527) | 1 504<br>(1 301 to 1 743) | -2.9<br>(-8.2 to 3.1) | 1 351 184<br>(1 176 579 to 1 553 355) | 3 486<br>(3 049 to 3 982) | -4.3<br>(-6.5 to -1.9) |
| Bahrain | 21 480<br>(18 176 to 25 283) | 1 558<br>(1 317 to 1 838) | 9.4<br>(3.7 to 15.5) | 58 143<br>(49 952 to 67 491) | 3 711<br>(3 217 to 4 280) | 9.4<br>(6.0 to 12.8) |
| Egypt | 1 323 510<br>(1 115 031 to 1 559 706) | 1 410<br>(1 215 to 1 627) | 8.8<br>(2.7 to 14.7) | 2 647 936<br>(2 288 775 to 3 056 336) | 3 249<br>(2 844 to 3 701) | 4.1<br>(1.3 to 6.9) |
| Iran | 1 344 586<br>(1 151 472 to 1 567 985) | 1 701<br>(1 462 to 1 976) | 7.9<br>(3.4 to 12.4) | 3 229 617<br>(2 806 087 to 3 697 598) | 3 899<br>(3 411 to 4 454) | 5.1<br>(3.6 to 6.9) |
| Iraq | 626 536<br>(512 072 to 759 959) | 1 378<br>(1 153 to 1 631) | 14.2<br>(5.7 to 22.8) | 1 218 473<br>(1 040 997 to 1 424 301) | 3 437<br>(2 979 to 3 958) | 19.4<br>(14.9 to 24.4) |
| Jordan | 147 491<br>(122 800 to 175 900) | 1 384<br>(1 183 to 1 618) | 1.7<br>(-5.1 to 8.0) | 293 031<br>(250 836 to 341 424) | 3 230<br>(2 805 to 3 700) | -0.8<br>(-3.5 to 2.2) |
| Kuwait | 74 673<br>(64 300 to 86 138) | 1 815<br>(1 570 to 2 110) | -4.9<br>(-10.3 to 0.5) | 180 593<br>(156 081 to 207 999) | 4 251<br>(3 720 to 4 860) | -4.9<br>(-7.3 to -2.3) |
| Lebanon | 134 232<br>(113 643 to 158 436) | 1 631<br>(1 407 to 1 906) | 8.9<br>(2.7 to 15.0) | 273 078<br>(234 315 to 317 558) | 3 572<br>(3 109 to 4 104) | 9.1<br>(5.8 to 13.0) |
| Libya | 107 284<br>(91 898 to 123 652) | 1 615<br>(1 400 to 1 844) | -6.0<br>(-11.6 to -0.6) | 230 536<br>(200 236 to 265 410) | 3 622<br>(3 175 to 4 132) | -7.6<br>(-9.6 to -5.6) |
| Morocco | 487 872<br>(417 555 to 570 750) | 1 408<br>(1 210 to 1 636) | 8.7<br>(2.2 to 14.5) | 1 149 026<br>(999 534 to 1 314 358) | 3 275<br>(2 861 to 3 725) | 5.5<br>(3.3 to 7.8) |
| Palestine | 70 758<br>(58 259 to 85 605) | 1 431<br>(1 224 to 1 672) | 3.7<br>(-2.6 to 10.1) | 130 092<br>(112 403 to 150 085) | 3 397<br>(2 969 to 3 871) | 3.8<br>(1.8 to 5.9) |
| Oman | 66 261<br>(55 336 to 80 190) | 1 582<br>(1 362 to 1 849) | 5.3<br>(-0.5 to 11.3) | 148 726<br>(126 285 to 174 723) | 3 574<br>(3 109 to 4 112) | 5.4<br>(2.7 to 8.1) |
| Qatar | 54 445<br>(45 831 to 64 277) | 2 011<br>(1 744 to 2 313) | -3.2<br>(-9.8 to 4.0) | 125 405<br>(106 851 to 146 810) | 4 603<br>(4 029 to 5 271) | -3.5<br>(-6.1 to -0.1) |
| Saudi Arabia | 695 984<br>(606 109 to 802 551) | 2 191<br>(1 922 to 2 493) | -6.4<br>(-11.4 to -1.0) | 1 412 818<br>(1 232 967 to 1 613 742) | 4 688<br>(4 161 to 5 312) | -8.6<br>(-10.5 to -6.9) |
| Sudan | 527 474<br>(439 000 to 639 987) | 1 303<br>(1 121 to 1 509) | 8.9<br>(3.2 to 14.5) | 945 064<br>(817 590 to 1 105 440) | 3 198<br>(2 802 to 3 670) | 7.7<br>(5.0 to 10.7) |
| Syria | 259 893<br>(213 922 to 316 693) | 1 404<br>(1 177 to 1 651) | 23.4<br>(16.6 to 30.4) | 543 105<br>(464 701 to 630 683) | 3 328<br>(2 880 to 3 833) | 20.8<br>(16.7 to 25.1) |
| Tunisia | 163 409<br>(140 390 to 189 712) | 1 474<br>(1 256 to 1 722) | 8.3<br>(2.8 to 15.0) | 413 032<br>(359 236 to 473 833) | 3 377<br>(2 932 to 3 881) | 4.6<br>(1.8 to 7.4) |
| Turkey | 1 276 654<br>(1 112 675 to 1 452 784) | 1 610<br>(1 393 to 1 859) | -11.6<br>(-17.3 to -5.0) | 3 045 977<br>(2 669 478 to 3 499 517) | 3 515<br>(3 072 to 4 038) | -17.9<br>(-21.1 to -14.7) |
| United Arab Emirates | 191 722<br>(161 644 to 226 252) | 2 010<br>(1 743 to 2 313) | -15.0<br>(-19.5 to -9.5) | 495 721<br>(426 385 to 574 778) | 4 616<br>(4 041 to 5 262) | -16.4<br>(-18.3 to -14.2) |
| Yemen | 406 535<br>(337 920 to 490 550) | 1 339<br>(1 157 to 1 546) | 2.6<br>(-2.4 to 8.4) | 701 073<br>(602 668 to 813 602) | 3 177<br>(2 783 to 3 610) | 1.3<br>(-1.1 to 4.3) |
| **South Asia** | **27 645 658<br>(23 929 866 to 31 790 195)** | **1 709<br>(1 495 to 1 959)** | **33.4<br>(27.9 to 38.9)** | **54 501 408<br>(48 091 376 to 61 867 360)** | **3 585<br>(3 184 to 4 064)** | **30.4<br>(28.2 to 32.9)** |
| **South Asia** | **27 645 658<br>(23 929 866 to 31 790 195)** | **1 709<br>(1 495 to 1 959)** | **33.4<br>(27.9 to 38.9)** | **54 501 408<br>(48 091 376 to 61 867 360)** | **3 585<br>(3 184 to 4 064)** | **30.4<br>(28.2 to 32.9)** |
| Bangladesh | 1 879 191<br>(1 600 626 to 2 201 983) | 1 237<br>(1 064 to 1 431) | 38.8<br>(30.2 to 47.4) | 3 975 599<br>(3 470 520 to 4 541 326) | 2 801<br>(2 465 to 3 176) | 42.5<br>(36.9 to 48.5) |
| Bhutan | 13 737<br>(12 000 to 15 729) | 1 627<br>(1 439 to 1 836) | 12.6<br>(6.1 to 19.7) | 26 689<br>(23 565 to 30 111) | 3 383<br>(3 020 to 3 804) | 9.9<br>(7.3 to 12.7) |
| India | 22 618 646<br>(19 574 098 to 26 132 069) | 1 793<br>(1 567 to 2 059) | 31.4<br>(25.8 to 37.1) | 44 741 891<br>(39 434 255 to 50 716 561) | 3 723<br>(3 313 to 4 225) | 28.0<br>(25.9 to 30.4) |
| Nepal | 349 043<br>(302 584 to 403 990) | 1 291<br>(1 136 to 1 468) | 15.8<br>(8.7 to 22.3) | 677 787<br>(600 780 to 764 528) | 2 726<br>(2 435 to 3 064) | 14.2<br>(11.3 to 17.0) |
| Pakistan | 2 785 040<br>(2 384 681 to 3 254 158) | 1 475<br>(1 288 to 1 681) | 44.9<br>(37.3 to 52.4) | 5 079 442<br>(4 444 064 to 5 736 010) | 3 244<br>(2 879 to 3 654) | 39.4<br>(35.6 to 43.1) |
| **Southeast Asia, East Asia, and Oceania** | **24 523 775<br>(21 530 658 to 27 957 854)** | **1 104<br>(971 to 1 262)** | **85.4<br>(77.8 to 93.1)** | **68 124 841<br>(60 201 836 to 77 289 748)** | **2 688<br>(2 375 to 3 036)** | **83.2<br>(75.8 to 91.0)** |
| **East Asia** | **21 903 706<br>(19 252 383 to 24 912 373)** | **1 462<br>(1 275 to 1 672)** | **111.4<br>(102.8 to 121.3)** | **62 282 056<br>(54 985 517 to 70 760 535)** | **3 375<br>(2 972 to 3 834)** | **99.1<br>(90.5 to 108.9)** |
| China | 21 032 439<br>(18 488 797 to 23 929 008) | 1 477<br>(1 288 to 1 691) | 113.2<br>(104.4 to 123.6) | 59 820 373<br>(52 811 012 to 67 990 803) | 3 411<br>(3 004 to 3 874) | 100.4<br>(91.6 to 110.3) |
| North Korea | 239 052<br>(206 380 to 274 852) | 935<br>(803 to 1 075) | 78.5<br>(66.4 to 90.6) | 661 540<br>(578 846 to 755 182) | 2 211<br>(1 930 to 2 515) | 80.9<br>(71.5 to 90.8) |
| Taiwan (Province of China) | 279 358<br>(248 092 to 314 981) | 1 126<br>(978 to 1 288) | 42.6<br>(30.2 to 55.8) | 796 814<br>(706 078 to 903 269) | 2 502<br>(2 203 to 2 827) | 39.3<br>(33.5 to 45.4) |
| **Oceania** | **85 737<br>(73 359 to 101 145)** | **708<br>(616 to 824)** | **89.2<br>(80.1 to 98.9)** | **164 350<br>(144 178 to 187 668)** | **1 690<br>(1 498 to 1 916)** | **91.9<br>(83.9 to 101.8)** |
| American Samoa | 431<br>(366 to 508) | 781<br>(672 to 910) | 65.8<br>(56.4 to 75.0) | 907<br>(795 to 1 029) | 1 797<br>(1 581 to 2 037) | 64.8<br>(58.6 to 72.1) |
| Federated States of Micronesia | 708<br>(599 to 839) | 692<br>(596 to 806) | 91.1<br>(79.5 to 104.4) | 1 477<br>(1 292 to 1 685) | 1 637<br>(1 441 to 1 849) | 92.3<br>(83.5 to 102.5) |
| Fiji | 6 054<br>(5 095 to 7 141) | 670<br>(568 to 787) | 100.9<br>(89.8 to 113.0) | 13 907<br>(12 174 to 15 879) | 1 612<br>(1 416 to 1 830) | 100.3<br>(89.3 to 112.9) |
| Guam | 1 534<br>(1 334 to 1 783) | 907<br>(791 to 1 052) | 89.5<br>(80.3 to 100.3) | 3 579<br>(3 165 to 4 029) | 2 031<br>(1 795 to 2 290) | 89.2<br>(80.2 to 98.3) |
| Kiribati | 685<br>(567 to 832) | 574<br>(481 to 688) | 118.0<br>(104.1 to 134.7) | 1 402<br>(1 223 to 1 598) | 1 450<br>(1 274 to 1 647) | 118.7<br>(105.5 to 133.5) |
| Marshall Islands | 385<br>(326 to 452) | 702<br>(608 to 813) | 99.7<br>(88.6 to 111.1) | 770<br>(675 to 880) | 1 653<br>(1 463 to 1 875) | 100.3<br>(90.5 to 110.2) |
| Northern Mariana Islands | 379<br>(323 to 442) | 833<br>(717 to 971) | 59.3<br>(50.7 to 67.9) | 972<br>(854 to 1 102) | 1 873<br>(1 651 to 2 131) | 58.5<br>(52.4 to 65.0) |
| Papua New Guinea | 58 834<br>(50 099 to 69 931) | 661<br>(573 to 772) | 102.3<br>(90.0 to 115.5) | 109 907<br>(96 082 to 125 691) | 1 583<br>(1 400 to 1 802) | 103.8<br>(93.9 to 116.7) |
| Samoa | 1 438<br>(1 211 to 1 710) | 739<br>(632 to 856) | 92.7<br>(82.3 to 104.1) | 2 872<br>(2 521 to 3 273) | 1 752<br>(1 548 to 1 989) | 93.1<br>(84.6 to 103.5) |
| Solomon Islands | 7 719<br>(6 839 to 8 769) | 1 344<br>(1 204 to 1 507) | 36.0<br>(28.8 to 44.1) | 13 900<br>(12 309 to 15 687) | 3 051<br>(2 726 to 3 441) | 37.6<br>(33.9 to 41.8) |
| Tonga | 719<br>(607 to 860) | 704<br>(600 to 827) | 89.1<br>(78.8 to 99.5) | 1 526<br>(1 339 to 1 731) | 1 678<br>(1 478 to 1 900) | 93.9<br>(84.2 to 104.3) |
| Vanuatu | 2 126<br>(1 813 to 2 511) | 776<br>(673 to 900) | 94.3<br>(84.1 to 105.9) | 4 075<br>(3 591 to 4 632) | 1 844<br>(1 644 to 2 077) | 95.1<br>(86.7 to 104.3) |
| **Southeast Asia** | **2 534 331<br>(2 181 508 to 2 958 504)** | **398<br>(344 to 463)** | **15.0<br>(10.5 to 20.2)** | **5 678 435<br>(5 027 741 to 6 452 084)** | **882<br>(786 to 1 002)** | **16.2<br>(13.9 to 18.6)** |
| Cambodia | 77 041<br>(66 794 to 89 252) | 523<br>(461 to 598) | 46.7<br>(37.1 to 56.8) | 153 486<br>(136 024 to 174 333) | 1 125<br>(1 004 to 1 277) | 46.5<br>(42.3 to 50.8) |
| Indonesia | 652 968<br>(543 971 to 789 394) | 273<br>(231 to 326) | -29.5<br>(-33.9 to -24.6) | 1 478 919<br>(1 300 607 to 1 728 999) | 613<br>(541 to 711) | -28.3<br>(-30.5 to -26.3) |

| Location | Incidence (95% UI) | | | Prevalence (95% UI) | | |
|---|---|---|---|---|---|---|
| | 2017 counts | 2017 age-standardised rates per 100,000 | Percentage change in age-standardised rates between 1990 and 2017 | 2017 counts | 2017 age-standardised rates per 100,000 | Percentage change in age-standardised rates between 1990 and 2017 |
| Laos | 27 395 (23 238 to 32 211) | 426 (368 to 493) | 63.0 (51.8 to 74.4) | 54 750 (48 334 to 62 213) | 966 (861 to 1 089) | 59.0 (53.7 to 64.3) |
| Malaysia | 123 322 (102 861 to 147 763) | 417 (351 to 496) | 70.1 (58.7 to 81.8) | 276 611 (243 961 to 317 962) | 938 (832 to 1 075) | 68.7 (60.2 to 76.9) |
| Maldives | 1 836 (1 528 to 2 213) | 424 (359 to 498) | 29.7 (20.1 to 42.1) | 4 071 (3 578 to 4 670) | 955 (845 to 1 086) | 27.4 (23.2 to 32.1) |
| Mauritius | 5 928 (5 154 to 6 933) | 463 (397 to 544) | 60.0 (50.0 to 70.4) | 16 068 (14 236 to 18 316) | 1 053 (929 to 1 199) | 57.5 (51.0 to 64.2) |
| Myanmar | 271 773 (238 553 to 312 106) | 536 (473 to 610) | 40.7 (33.3 to 49.9) | 581 330 (516 566 to 655 758) | 1 161 (1 038 to 1 306) | 38.1 (34.2 to 41.8) |
| Philippines | 364 928 (308 087 to 434 894) | 369 (315 to 433) | 87.7 (74.5 to 102.1) | 751 580 (658 588 to 863 634) | 837 (738 to 955) | 81.7 (73.7 to 89.5) |
| Sri Lanka | 115 106 (100 678 to 132 382) | 523 (456 to 601) | 50.3 (39.6 to 61.1) | 261 390 (232 626 to 297 059) | 1 104 (984 to 1 253) | 43.9 (39.5 to 49.1) |
| Seychelles | 511 (445 to 595) | 499 (432 to 582) | 56.8 (47.7 to 67.1) | 1 213 (1 071 to 1 375) | 1 099 (974 to 1 246) | 54.8 (49.2 to 61.2) |
| Thailand | 329 474 (283 509 to 380 917) | 447 (382 to 523) | 32.7 (23.6 to 42.8) | 879 742 (782 178 to 999 750) | 992 (880 to 1 125) | 34.7 (30.2 to 39.9) |
| Timor-Leste | 5 119 (4 262 to 6 119) | 428 (367 to 500) | 80.3 (67.9 to 92.5) | 9 540 (8 439 to 10 869) | 970 (864 to 1 098) | 72.9 (66.3 to 80.4) |
| Vietnam | 555 596 (487 358 to 634 919) | 592 (520 to 677) | 53.8 (45.3 to 63.5) | 1 202 264 (1 073 553 to 1 355 875) | 1 231 (1 102 to 1 387) | 53.7 (48.8 to 59.1) |
| **Sub-Saharan Africa** | **13 312 641 (11 202 194 to 15 911 838)** | **1 585 (1 387 to 1 817)** | **0.6 (-3.4 to 4.5)** | **22 179 916 (19 338 138 to 25 333 682)** | **3 365 (2 988 to 3 792)** | **1.3 (0.2 to 2.3)** |
| **Central sub-Saharan Africa** | **1 384 416 (1 159 424 to 1 647 365)** | **1 439 (1 254 to 1 643)** | **5.4 (0.8 to 10.0)** | **2 308 971 (2 016 706 to 2 627 336)** | **3 047 (2 712 to 3 430)** | **6.0 (4.3 to 7.5)** |
| Angola | 337 738 (284 330 to 401 232) | 1 645 (1 447 to 1 861) | 1.8 (-3.3 to 7.2) | 534 079 (469 414 to 607 797) | 3 357 (2 988 to 3 776) | -0.4 (-2.0 to 1.4) |
| Central African Republic | 49 775 (41 838 to 59 024) | 1 302 (1 137 to 1 493) | 7.5 (1.5 to 13.3) | 87 815 (76 841 to 100 310) | 2 816 (2 504 to 3 161) | 10.8 (8.2 to 13.4) |
| Congo (Brazzaville) | 60 930 (51 862 to 71 202) | 1 534 (1 332 to 1 753) | -1.8 (-7.1 to 3.5) | 109 468 (96 246 to 124 071) | 3 169 (2 820 to 3 549) | -0.4 (-2.2 to 1.3) |
| DR Congo | 894 868 (744 923 to 1 072 234) | 1 365 (1 181 to 1 568) | 6.9 (1.5 to 12.5) | 1 507 505 (1 315 899 to 1 719 802) | 2 941 (2 614 to 3 310) | 8.4 (6.2 to 10.5) |
| Equatorial Guinea | 16 784 (14 064 to 20 174) | 1 683 (1 477 to 1 916) | 30.0 (22.8 to 37.4) | 25 192 (22 071 to 28 725) | 3 291 (2 930 to 3 707) | 19.8 (17.0 to 22.7) |
| Gabon | 24 321 (20 955 to 28 161) | 1 696 (1 485 to 1 929) | -11.4 (-16.7 to -5.8) | 44 911 (39 614 to 50 755) | 3 471 (3 091 to 3 900) | -9.0 (-10.6 to -7.3) |
| **Eastern sub-Saharan Africa** | **6 257 478 (5 253 032 to 7 503 431)** | **2 026 (1 780 to 2 315)** | **1.5 (-2.5 to 5.1)** | **10 221 577 (8 919 131 to 11 657 411)** | **4 269 (3 786 to 4 813)** | **3.1 (1.6 to 4.4)** |
| Burundi | 159 367 (132 203 to 193 238) | 1 835 (1 595 to 2 096) | -9.0 (-14.6 to -3.7) | 261 510 (227 444 to 300 475) | 3 942 (3 496 to 4 447) | -4.5 (-6.5 to -2.2) |
| Comoros | 12 392 (10 567 to 14 644) | 1 952 (1 705 to 2 245) | -5.7 (-11.2 to -0.5) | 23 561 (20 684 to 26 718) | 4 172 (3 707 to 4 708) | -5.1 (-7.1 to -2.9) |
| Djibouti | 18 893 (16 201 to 22 165) | 2 079 (1 830 to 2 378) | -5.5 (-10.9 to 0.2) | 36 025 (31 538 to 41 136) | 4 390 (3 899 to 4 945) | -4.3 (-5.9 to -2.5) |
| Eritrea | 93 626 (79 068 to 110 944) | 2 028 (1 790 to 2 297) | 2.5 (-2.8 to 8.1) | 154 710 (134 738 to 177 045) | 4 167 (3 708 to 4 675) | 1.8 (0.2 to 3.6) |
| Ethiopia | 1 462 066 (1 219 718 to 1 768 436) | 1 861 (1 614 to 2 167) | -7.3 (-11.6 to -2.7) | 2 378 021 (2 071 706 to 2 715 632) | 3 908 (3 457 to 4 411) | -4.9 (-6.6 to -3.1) |
| Kenya | 953 132 (803 115 to 1 132 723) | 2 437 (2 121 to 2 803) | 11.2 (6.8 to 15.1) | 1 638 927 (1 417 177 to 1 896 755) | 5 268 (4 636 to 6 012) | 13.4 (11.2 to 15.6) |
| Madagascar | 406 930 (338 817 to 489 470) | 1 902 (1 661 to 2 177) | -2.5 (-8.6 to 3.0) | 679 831 (591 095 to 777 498) | 4 120 (3 638 to 4 638) | -3.1 (-5.3 to -0.6) |
| Malawi | 267 854 (226 087 to 319 449) | 1 932 (1 712 to 2 190) | 1.1 (-3.7 to 6.2) | 436 216 (382 584 to 498 067) | 3 998 (3 554 to 4 498) | 2.8 (0.7 to 4.7) |
| Mozambique | 489 770 (409 743 to 587 058) | 2 152 (1 891 to 2 444) | 9.9 (4.0 to 16.0) | 748 843 (654 444 to 851 070) | 4 364 (3 889 to 4 898) | 7.1 (5.2 to 9.4) |
| Rwanda | 198 008 (166 126 to 238 037) | 1 938 (1 699 to 2 225) | -8.4 (-13.9 to -2.9) | 338 370 (295 308 to 387 167) | 4 053 (3 591 to 4 559) | -6.6 (-8.7 to -4.8) |
| Somalia | 248 751 (206 588 to 303 438) | 1 842 (1 608 to 2 109) | 8.1 (2.9 to 12.8) | 404 757 (351 109 to 464 115) | 4 001 (3 534 to 4 505) | 9.7 (7.7 to 11.9) |
| South Sudan | 160 057 (134 678 to 194 096) | 2 035 (1 785 to 2 337) | 1.6 (-3.8 to 6.5) | 255 470 (222 993 to 291 637) | 4 347 (3 859 to 4 901) | 4.4 (2.3 to 6.5) |
| Tanzania | 906 582 (773 546 to 1 081 057) | 2 099 (1 857 to 2 376) | 2.6 (-2.9 to 8.0) | 1 496 145 (1 310 157 to 1 703 384) | 4 362 (3 885 to 4 911) | 3.4 (1.7 to 5.0) |
| Uganda | 590 422 (491 649 to 721 514) | 1 886 (1 651 to 2 170) | 13.0 (8.2 to 17.7) | 905 569 (785 266 to 1 042 560) | 3 998 (3 537 to 4 494) | 15.1 (12.8 to 17.6) |
| Zambia | 285 704 (243 159 to 342 294) | 2 193 (1 946 to 2 487) | -4.9 (-10.1 to 0.4) | 457 210 (398 984 to 525 604) | 4 458 (3 965 to 5 011) | -3.7 (-5.7 to -1.7) |
| **Southern sub-Saharan Africa** | **783 539 (660 901 to 927 509)** | **1 094 (939 to 1 277)** | **6.9 (3.4 to 10.3)** | **1 588 819 (1 386 207 to 1 814 625)** | **2 364 (2 084 to 2 685)** | **10.0 (8.2 to 12.1)** |
| Botswana | 22 497 (18 858 to 26 839) | 1 121 (961 to 1 304) | 3.9 (-1.4 to 9.4) | 44 067 (38 357 to 50 964) | 2 349 (2 071 to 2 672) | 3.6 (1.3 to 6.4) |
| Lesotho | 18 638 (15 651 to 22 158) | 1 081 (933 to 1 248) | 16.9 (12.1 to 21.9) | 35 456 (30 871 to 40 448) | 2 285 (2 018 to 2 583) | 15.0 (12.6 to 17.9) |
| Namibia | 23 192 (19 459 to 27 532) | 1 110 (959 to 1 289) | 1.3 (-4.3 to 6.6) | 42 942 (37 528 to 49 369) | 2 335 (2 072 to 2 645) | 2.1 (-0.3 to 4.7) |
| South Africa | 565 374 (477 501 to 669 633) | 1 086 (928 to 1 278) | 7.7 (3.6 to 11.5) | 1 199 313 (1 048 571 to 1 371 373) | 2 354 (2 076 to 2 674) | 10.6 (8.6 to 12.9) |
| Swaziland | 10 647 (8 842 to 12 717) | 1 102 (955 to 1 271) | 6.1 (1.2 to 11.6) | 18 886 (16 545 to 21 681) | 2 314 (2 054 to 2 623) | 6.7 (4.4 to 9.0) |
| Zimbabwe | 143 192 (119 532 to 172 513) | 1 168 (1 018 to 1 349) | 6.8 (1.1 to 11.7) | 248 155 (214 732 to 283 358) | 2 469 (2 181 to 2 789) | 9.9 (7.3 to 12.4) |
| **Western sub-Saharan Africa** | **4 887 207 (4 090 246 to 5 814 346)** | **1 377 (1 202 to 1 573)** | **-4.6 (-9.4 to 0.4)** | **8 060 548 (7 056 412 to 9 186 624)** | **2 964 (2 631 to 3 333)** | **-5.2 (-6.3 to -4.1)** |
| Benin | 124 717 (103 339 to 150 605) | 1 291 (1 115 to 1 487) | -5.6 (-11.7 to 0.0) | 205 588 (178 823 to 234 573) | 2 842 (2 513 to 3 193) | -4.7 (-6.3 to -2.9) |
| Burkina Faso | 261 546 (224 754 to 307 546) | 1 620 (1 436 to 1 830) | -4.0 (-8.9 to 1.1) | 421 164 (371 412 to 475 402) | 3 334 (2 980 to 3 739) | -4.1 (-5.7 to -2.5) |
| Cameroon | 316 255 (265 044 to 377 492) | 1 382 (1 211 to 1 587) | -11.5 (-17.0 to -6.1) | 524 826 (458 718 to 600 423) | 2 977 (2 642 to 3 355) | -10.2 (-11.9 to -8.4) |
| Cape Verde | 6 579 (5 539 to 7 758) | 1 222 (1 049 to 1 425) | 11.6 (6.7 to 16.3) | 13 633 (12 003 to 15 484) | 2 709 (2 396 to 3 067) | 9.8 (7.4 to 12.1) |
| Chad | 160 558 (132 985 to 194 187) | 1 325 (1 156 to 1 520) | 6.5 (1.0 to 11.8) | 250 268 (218 617 to 283 549) | 2 962 (2 625 to 3 329) | 8.6 (6.3 to 10.9) |
| Cote d'Ivoire | 280 607 (235 856 to 334 523) | 1 369 (1 193 to 1 563) | -8.3 (-13.7 to -2.6) | 486 432 (424 637 to 558 341) | 2 991 (2 652 to 3 366) | -7.3 (-8.9 to -5.3) |
| The Gambia | 24 003 (19 807 to 28 674) | 1 319 (1 139 to 1 514) | -2.8 (-8.4 to 2.0) | 40 273 (35 115 to 45 899) | 2 885 (2 554 to 3 246) | -2.8 (-4.6 to -0.8) |
| Ghana | 393 890 (337 465 to 462 131) | 1 584 (1 396 to 1 796) | 10.2 (4.6 to 16.1) | 689 807 (608 315 to 781 358) | 3 246 (2 888 to 3 645) | 6.3 (4.2 to 8.4) |
| Guinea | 129 686 (108 858 to 154 865) | 1 304 (1 135 to 1 497) | -5.1 (-10.2 to -0.2) | 219 808 (192 536 to 250 433) | 2 895 (2 573 to 3 263) | -4.5 (-6.3 to -2.4) |
| Guinea-Bissau | 18 835 (15 649 to 22 676) | 1 234 (1 077 to 1 422) | -11.3 (-16.9 to -5.9) | 31 297 (27 174 to 36 023) | 2 713 (2 397 to 3 059) | -10.4 (-12.2 to -8.7) |
| Liberia | 49 085 (40 484 to 59 226) | 1 204 (1 038 to 1 400) | -7.6 (-14.3 to -1.4) | 84 817 (73 614 to 97 168) | 2 702 (2 374 to 3 044) | -6.5 (-8.4 to -4.5) |
| Mali | 214 790 (176 951 to 259 269) | 1 276 (1 103 to 1 469) | -3.2 (-9.5 to 2.4) | 346 839 (302 244 to 397 309) | 2 841 (2 504 to 3 208) | -2.7 (-5.0 to -0.6) |
| Mauritania | 45 865 (38 411 to 54 923) | 1 352 (1 175 to 1 566) | -11.8 (-17.7 to -5.4) | 79 675 (69 860 to 90 178) | 2 965 (2 635 to 3 323) | -10.2 (-12.0 to -8.1) |

| Location | Incidence (95% UI) | | | Prevalence (95% UI) | | |
|---|---|---|---|---|---|---|
| | 2017 counts | 2017 age-standardised rates per 100,000 | Percentage change in age-standardised rates between 1990 and 2017 | 2017 counts | 2017 age-standardised rates per 100,000 | Percentage change in age-standardised rates between 1990 and 2017 |
| Niger | 216 174 (176 516 to 268 586) | 1 248 (1 081 to 1 452) | -5.5 (-11.6 to 1.0) | 331 276 (288 335 to 378 987) | 2 805 (2 485 to 3 159) | -5.1 (-7.0 to -3.1) |
| Nigeria | 2 309 653 (1 926 332 to 2 758 290) | 1 370 (1 183 to 1 576) | -5.9 (-12.0 to -0.1) | 3 751 042 (3 287 478 to 4 287 688) | 2 937 (2 604 to 3 310) | -7.2 (-8.9 to -5.4) |
| Sao Tome and Principe | 3 436 (3 000 to 3 979) | 1 975 (1 755 to 2 234) | -1.4 (-7.1 to 4.9) | 6 000 (5 300 to 6 800) | 4 192 (3 724 to 4 736) | -6.3 (-8.5 to -4.0) |
| Senegal | 166 558 (139 141 to 197 758) | 1 331 (1 155 to 1 532) | -6.9 (-12.4 to -0.5) | 290 933 (255 447 to 329 724) | 2 917 (2 597 to 3 276) | -7.1 (-8.6 to -5.5) |
| Sierra Leone | 83 184 (69 008 to 99 780) | 1 258 (1 086 to 1 447) | -5.6 (-11.5 to -0.2) | 142 636 (124 135 to 162 832) | 2 772 (2 449 to 3 129) | -5.8 (-7.6 to -4.1) |
| Togo | 81 738 (67 864 to 98 041) | 1 256 (1 088 to 1 452) | -5.8 (-11.7 to -0.1) | 144 156 (125 451 to 164 284) | 2 754 (2 438 to 3 094) | -5.0 (-6.8 to -3.0) |
