# Supplementary material for: The global burden of falls: global, regional and national estimates of morbidity and mortality from the Global Burden of Disease Study 2017
Source: Inj Prev. 2020 Jan 15;26(Suppl 1):i3–i11. doi: 10.1136/injuryprev-2019-043286 (PMC7571347; doi:10.1136/injuryprev-2019-043286)
Supplement: Supplementary data [file injuryprev-2019-043286supp004.pdf]

Table 2: Mortality for 2017 and percentage change of age-standardised rates between 1990 and 2017 by location for falls			
Location	Mortality (95% UI)		
	2017 counts	2017 age-standardised rates per 100,000	Percentage change in age-standardised rates between 1990 and 2017
**Global**	**695 771 (644 927 to 741 720)**	**9.2 (8.5 to 9.8)**	**-5.9 (-13.7 to 3.5)**
**Low SDI**	**93 850 (86 459 to 104 963)**	**15.1 (13.9 to 17.1)**	**-10.8 (-22.4 to 6.0)**
**Low-middle SDI**	**157 311 (144 698 to 175 240)**	**15.3 (14.1 to 17.2)**	**-2.7 (-19.3 to 18.3)**
**Middle SDI**	**192 591 (167 707 to 212 051)**	**10.1 (8.9 to 11.0)**	**-8.3 (-17.6 to 5.7)**
**High-middle SDI**	**105 593 (93 147 to 112 263)**	**6.7 (5.9 to 7.1)**	**-2.2 (-19.4 to 9.2)**
**High SDI**	**143 826 (140 311 to 147 243)**	**5.7 (5.6 to 5.9)**	**-16.6 (-18.8 to -14.4)**
**Central Europe, Eastern Europe, and Central Asia**	**35 374 (34 224 to 36 301)**	**6.3 (6.1 to 6.5)**	**-25.8 (-28.2 to -23.6)**
**Central Asia**	**2 868 (2 682 to 3 094)**	**3.6 (3.4 to 3.9)**	**-33.5 (-38.0 to -27.9)**
Armenia	88 (83 to 94)	2.4 (2.2 to 2.5)	-68.5 (-71.1 to -65.8)
Azerbaijan	258 (205 to 360)	2.9 (2.3 to 3.8)	-32.3 (-48.4 to -3.1)
Georgia	262 (243 to 280)	5.1 (4.7 to 5.4)	-17.4 (-24.8 to -9.6)
Kazakhstan	780 (706 to 851)	4.5 (4.1 to 4.8)	-18.0 (-25.3 to -11.0)
Kyrgyzstan	132 (120 to 144)	2.5 (2.3 to 2.7)	-65.3 (-68.5 to -61.8)
Mongolia	206 (149 to 253)	6.9 (5.1 to 8.4)	-9.1 (-27.4 to 14.5)
Tajikistan	311 (269 to 388)	4.0 (3.5 to 4.9)	-34.6 (-45.7 to -11.4)
Turkmenistan	106 (94 to 118)	2.6 (2.3 to 2.8)	-35.7 (-43.5 to -28.0)
Uzbekistan	725 (635 to 820)	2.9 (2.6 to 3.3)	-36.1 (-43.7 to -28.2)
**Central Europe**	**14 438 (13 990 to 14 908)**	**7.1 (6.9 to 7.4)**	**-47.0 (-48.9 to -45.0)**
Albania	80 (64 to 101)	2.2 (1.8 to 2.7)	-3.1 (-24.2 to 21.2)
Bosnia and Herzegovina	118 (103 to 136)	2.2 (2.0 to 2.5)	-21.3 (-41.6 to 15.0)
Bulgaria	501 (462 to 540)	4.2 (3.9 to 4.6)	-30.7 (-36.3 to -24.6)
Croatia	1 150 (1 072 to 1 237)	12.8 (11.9 to 13.7)	24.9 (14.4 to 35.9)
Czech Republic	1 461 (1 346 to 1 580)	7.2 (6.6 to 7.7)	-72.5 (-74.6 to -70.2)
Hungary	1 874 (1 767 to 1 984)	9.4 (8.8 to 9.9)	-69.1 (-71.1 to -67.1)
Macedonia	99 (80 to 112)	3.2 (2.6 to 3.7)	36.5 (-12.3 to 73.0)
Montenegro	26 (22 to 30)	2.9 (2.5 to 3.4)	-19.0 (-31.6 to -4.0)
Poland	5 567 (5 195 to 5 967)	8.2 (7.7 to 8.8)	-31.9 (-36.9 to -26.3)
Romania	1 677 (1 567 to 1 787)	5.4 (5.0 to 5.8)	-39.8 (-44.2 to -35.6)
Serbia	584 (485 to 649)	3.9 (3.3 to 4.3)	3.2 (-11.3 to 19.9)
Slovakia	758 (638 to 856)	9.1 (7.7 to 10.3)	-46.1 (-53.3 to -34.9)
Slovenia	543 (501 to 589)	11.6 (10.7 to 12.5)	-33.3 (-38.9 to -27.2)

Location	Mortality (95% UI)		
	2017 counts	2017 age-standardised rates per 100,000	Percentage change in age-standardised rates between 1990 and 2017
**Eastern Europe**	**18 067 (17 367 to 18 629)**	**6.3 (6.0 to 6.5)**	**-0.6 (-4.5 to 2.5)**
Belarus	982 (884 to 1 076)	7.3 (6.5 to 8.0)	7.7 (-3.6 to 18.5)
Estonia	119 (103 to 136)	5.4 (4.7 to 6.2)	-54.6 (-61.0 to -47.6)
Latvia	244 (216 to 274)	7.3 (6.4 to 8.2)	-49.5 (-55.9 to -42.8)
Lithuania	474 (441 to 506)	9.5 (8.8 to 10.2)	-17.3 (-23.9 to -10.2)
Moldova	249 (232 to 267)	4.8 (4.5 to 5.2)	-45.9 (-49.8 to -41.7)
Russian Federation	12 477 (12 058 to 12 822)	6.3 (6.1 to 6.5)	11.6 (4.9 to 15.3)
Ukraine	3 522 (3 290 to 3 790)	5.8 (5.4 to 6.3)	-16.7 (-22.8 to -9.3)
**High-income**	**131 213 (127 836 to 134 821)**	**5.4 (5.3 to 5.6)**	**-12.9 (-15.4 to -10.4)**
**Australasia**	**3 739 (3 431 to 4 043)**	**6.5 (5.9 to 7.0)**	**32.1 (20.8 to 44.4)**
Australia	3 172 (2 863 to 3 476)	6.5 (5.8 to 7.1)	41.8 (28.0 to 58.0)
New Zealand	567 (527 to 610)	6.6 (6.1 to 7.1)	0.6 (-7.2 to 8.7)
**High-income Asia-Pacific**	**16 160 (15 448 to 16 859)**	**3.4 (3.2 to 3.5)**	**-24.1 (-27.9 to -20.1)**
Brunei	19 (17 to 22)	5.9 (5.3 to 7.0)	-16.5 (-29.7 to 2.7)
Japan	12 142 (11 671 to 12 623)	2.9 (2.8 to 3.1)	-20.3 (-23.7 to -16.6)
South Korea	3 834 (3 477 to 4 196)	5.0 (4.6 to 5.5)	-22.1 (-29.6 to -14.5)
Singapore	165 (151 to 179)	2.6 (2.3 to 2.8)	-26.8 (-33.5 to -20.1)
**High-income North America**	**44 300 (43 240 to 45 357)**	**6.6 (6.4 to 6.7)**	**57.2 (52.9 to 61.3)**
Canada	5 922 (5 481 to 6 406)	7.6 (7.0 to 8.2)	22.0 (11.6 to 33.0)
Greenland	8 (8 to 10)	15.8 (14.3 to 18.0)	-22.5 (-33.1 to -7.7)
USA	38 368 (37 389 to 39 312)	6.4 (6.3 to 6.6)	61.0 (56.5 to 65.2)
**Southern Latin America**	**3 143 (2 919 to 3 379)**	**3.8 (3.5 to 4.1)**	**-22.9 (-29.0 to -16.5)**
Argentina	1 619 (1 464 to 1 798)	3.0 (2.7 to 3.3)	-37.6 (-44.0 to -30.4)
Chile	1 274 (1 147 to 1 411)	5.6 (5.0 to 6.2)	8.8 (-3.1 to 20.9)
Uruguay	250 (223 to 277)	4.2 (3.8 to 4.7)	-16.3 (-25.8 to -5.9)
**Western Europe**	**63 871 (61 445 to 66 265)**	**5.9 (5.7 to 6.1)**	**-28.2 (-31.3 to -25.1)**
Andorra	9 (7 to 11)	5.6 (4.6 to 6.9)	-16.0 (-31.2 to 3.6)
Austria	1 408 (1 302 to 1 522)	7.1 (6.6 to 7.6)	-43.8 (-48.3 to -39.1)
Belgium	2 156 (1 993 to 2 329)	8.1 (7.5 to 8.7)	9.5 (0.1 to 19.2)
Cyprus	87 (75 to 98)	4.7 (4.1 to 5.3)	-36.1 (-47.6 to -18.9)
Denmark	827 (765 to 898)	6.5 (6.0 to 7.0)	-58.7 (-62.1 to -54.6)
Finland	1 339 (1 252 to 1 433)	10.2 (9.5 to 10.9)	-15.1 (-21.4 to -8.0)

Location	Mortality (95% UI)		
	**2017 counts**	**2017 age-standardised rates per 100,000**	**Percentage change in age-standardised rates between 1990 and 2017**
France	13 812 (12 711 to 14 953)	7.8 (7.2 to 8.4)	-38.6 (-44.1 to -32.5)
Germany	14 328 (12 997 to 15 881)	6.5 (5.9 to 7.2)	-23.6 (-31.9 to -14.6)
Greece	672 (626 to 723)	2.8 (2.6 to 3.0)	-41.0 (-45.2 to -36.6)
Iceland	35 (33 to 38)	5.8 (5.4 to 6.2)	-7.1 (-14.7 to 1.5)
Ireland	323 (296 to 352)	4.4 (4.0 to 4.8)	-32.5 (-38.4 to -25.6)
Israel	369 (339 to 399)	2.9 (2.7 to 3.2)	-29.3 (-35.4 to -22.5)
Italy	7 665 (7 068 to 8 314)	4.2 (3.9 to 4.5)	-45.4 (-50.0 to -40.4)
Luxembourg	82 (73 to 92)	7.7 (6.9 to 8.6)	-17.0 (-26.8 to -6.6)
Malta	55 (51 to 60)	6.4 (5.9 to 6.9)	-17.8 (-25.2 to -10.0)
Netherlands	3 465 (3 203 to 3 740)	9.2 (8.6 to 10.0)	41.6 (29.3 to 55.3)
Norway	1 014 (973 to 1 055)	8.8 (8.4 to 9.2)	-21.4 (-25.1 to -17.9)
Portugal	784 (722 to 843)	3.2 (2.9 to 3.4)	-52.4 (-56.6 to -48.4)
Spain	3 996 (3 708 to 4 301)	3.6 (3.3 to 3.8)	-19.7 (-26.0 to -13.5)
Sweden	1 670 (1 560 to 1 786)	6.6 (6.2 to 7.0)	2.8 (-4.8 to 11.1)
Switzerland	1 929 (1 785 to 2 083)	9.3 (8.6 to 10.0)	-41.1 (-46.0 to -35.7)
United Kingdom	7 781 (7 617 to 7 958)	5.4 (5.3 to 5.6)	12.3 (9.5 to 15.1)
**Latin America and Caribbean**	**35 929 (34 977 to 36 769)**	**6.5 (6.3 to 6.6)**	**-22.5 (-25.0 to -20.3)**
**Andean Latin America**	**2 568 (2 263 to 2 824)**	**4.6 (4.1 to 5.1)**	**-14.8 (-27.1 to -3.1)**
Bolivia	518 (387 to 653)	6.2 (4.7 to 7.8)	-22.0 (-38.3 to -0.3)
Ecuador	966 (870 to 1 070)	6.5 (5.9 to 7.2)	-19.8 (-27.8 to -10.6)
Peru	1 084 (818 to 1 312)	3.4 (2.6 to 4.1)	-5.8 (-34.1 to 20.5)
**Caribbean**	**3 800 (3 485 to 4 103)**	**7.4 (6.8 to 8.0)**	**2.8 (-5.5 to 11.6)**
Antigua and Barbuda	2 (2 to 2)	2.2 (2.1 to 2.4)	7.9 (-3.0 to 20.0)
The Bahamas	13 (12 to 15)	4.1 (3.7 to 4.5)	7.9 (-3.5 to 20.7)
Barbados	13 (12 to 14)	2.7 (2.5 to 3.0)	9.9 (-1.2 to 21.5)
Belize	12 (11 to 12)	4.5 (4.1 to 4.8)	11.4 (-2.3 to 24.8)
Bermuda	4 (3 to 4)	3.0 (2.7 to 3.2)	-17.7 (-26.5 to -8.7)
Cuba	2 592 (2 341 to 2 852)	12.9 (11.6 to 14.2)	12.2 (0.4 to 25.2)
Dominica	3 (3 to 3)	3.3 (3.0 to 3.6)	37.0 (22.0 to 52.3)
Dominican Republic	188 (152 to 245)	2.0 (1.6 to 2.6)	-17.1 (-34.3 to 13.9)
Grenada	7 (7 to 8)	4.3 (4.0 to 4.6)	-4.6 (-13.6 to 5.1)
Guyana	40 (35 to 45)	7.3 (6.5 to 8.2)	-2.6 (-15.1 to 10.8)

Location	Mortality (95% UI)		
	2017 counts	2017 age-standardised rates per 100,000	Percentage change in age-standardised rates between 1990 and 2017
Haiti	315<br>(223 to 425)	5.6<br>(4.1 to 7.6)	-17.1<br>(-32.6 to 2.3)
Jamaica	69<br>(57 to 79)	2.2<br>(1.8 to 2.6)	35.0<br>(9.5 to 58.0)
Puerto Rico	304<br>(280 to 328)	4.3<br>(3.9 to 4.6)	0.9<br>(-7.6 to 10.0)
Saint Lucia	6<br>(6 to 7)	3.2<br>(2.9 to 3.4)	-4.6<br>(-13.6 to 5.5)
Saint Vincent and the Grenadines	7<br>(7 to 8)	5.3<br>(4.8 to 5.7)	24.8<br>(11.9 to 38.9)
Suriname	27<br>(24 to 30)	5.2<br>(4.7 to 5.8)	10.5<br>(-3.0 to 23.9)
Trinidad and Tobago	54<br>(44 to 64)	3.3<br>(2.8 to 4.0)	-26.5<br>(-39.5 to -12.4)
Virgin Islands	8<br>(7 to 9)	5.2<br>(4.4 to 5.8)	5.2<br>(-13.8 to 22.4)
**Central Latin America**	**12 069<br>(11 495 to 12 478)**	**5.3<br>(5.0 to 5.4)**	**-43.0<br>(-46.7 to -40.8)**
Colombia	1 808<br>(1 606 to 2 037)	3.4<br>(3.0 to 3.8)	-52.6<br>(-58.3 to -46.5)
Costa Rica	324<br>(289 to 350)	6.4<br>(5.7 to 7.0)	-32.7<br>(-40.5 to -25.7)
El Salvador	486<br>(398 to 582)	8.2<br>(6.7 to 9.8)	-8.1<br>(-25.8 to 22.2)
Guatemala	849<br>(754 to 946)	7.7<br>(6.9 to 8.5)	-27.5<br>(-36.1 to -18.8)
Honduras	185<br>(139 to 253)	3.0<br>(2.2 to 4.2)	-18.5<br>(-42.7 to 15.2)
Mexico	6 827<br>(6 405 to 7 016)	6.2<br>(5.8 to 6.4)	-43.3<br>(-47.9 to -41.5)
Nicaragua	220<br>(190 to 253)	4.7<br>(4.1 to 5.4)	-19.2<br>(-30.8 to -2.9)
Panama	154<br>(142 to 167)	3.8<br>(3.5 to 4.1)	-32.5<br>(-39.0 to -25.6)
Venezuela	1 216<br>(1 064 to 1 400)	4.6<br>(4.0 to 5.2)	-35.4<br>(-43.9 to -25.6)
**Tropical Latin America**	**17 493<br>(16 920 to 17 936)**	**7.9<br>(7.7 to 8.1)**	**-8.2<br>(-11.8 to -5.2)**
Brazil	17 200<br>(16 626 to 17 651)	8.0<br>(7.7 to 8.2)	-8.8<br>(-12.5 to -5.8)
Paraguay	293<br>(224 to 349)	5.8<br>(4.5 to 7.0)	34.2<br>(1.7 to 69.1)
**North Africa and Middle East**	**21 444<br>(17 796 to 23 324)**	**4.9<br>(4.0 to 5.4)**	**-19.0<br>(-28.7 to 5.0)**
**North Africa and Middle East**	**21 444<br>(17 796 to 23 324)**	**4.9<br>(4.0 to 5.4)**	**-19.0<br>(-28.7 to 5.0)**
Afghanistan	2 540<br>(2 091 to 3 098)	13.8<br>(12.0 to 16.0)	-20.1<br>(-40.7 to 38.9)
Algeria	1 274<br>(1 017 to 1 635)	3.7<br>(3.1 to 4.7)	-18.9<br>(-31.9 to 7.1)
Bahrain	18<br>(12 to 22)	1.6<br>(1.2 to 2.0)	-37.9<br>(-50.1 to -15.4)
Egypt	2 750<br>(1 971 to 3 597)	4.4<br>(3.1 to 5.8)	-13.5<br>(-31.5 to 10.3)
Iran	2 715<br>(2 547 to 3 135)	3.9<br>(3.7 to 4.5)	-26.3<br>(-36.1 to -5.2)
Iraq	352<br>(308 to 427)	1.3<br>(1.1 to 1.4)	-56.9<br>(-69.2 to -34.3)
Jordan	165<br>(144 to 192)	2.4<br>(2.1 to 2.8)	-34.2<br>(-46.8 to -14.1)
Kuwait	94<br>(84 to 105)	2.4<br>(2.1 to 2.6)	-23.9<br>(-32.0 to -14.8)
Lebanon	304<br>(186 to 496)	5.8<br>(3.6 to 8.8)	-25.0<br>(-52.7 to 12.3)

Location	Mortality (95% UI)		
	2017 counts	2017 age-standardised rates per 100,000	Percentage change in age-standardised rates between 1990 and 2017
Libya	239<br>(149 to 325)	4.6<br>(3.0 to 6.2)	-15.5<br>(-46.5 to 13.4)
Morocco	1 251<br>(975 to 1 571)	4.1<br>(3.2 to 5.1)	-11.3<br>(-28.9 to 16.7)
Palestine	115<br>(82 to 137)	3.5<br>(2.8 to 4.0)	-29.9<br>(-43.9 to -11.4)
Oman	75<br>(60 to 93)	3.2<br>(2.5 to 3.9)	-26.8<br>(-47.4 to 6.6)
Qatar	78<br>(41 to 108)	3.6<br>(2.6 to 4.6)	-25.3<br>(-46.1 to 3.6)
Saudi Arabia	1 785<br>(1 159 to 2 522)	8.8<br>(5.5 to 12.2)	-18.4<br>(-52.1 to 33.1)
Sudan	1 199<br>(886 to 1 609)	4.5<br>(3.3 to 6.1)	-21.8<br>(-41.9 to 10.4)
Syria	274<br>(218 to 337)	2.1<br>(1.7 to 2.7)	-24.1<br>(-41.8 to 3.1)
Tunisia	347<br>(265 to 434)	3.1<br>(2.4 to 3.9)	-17.6<br>(-37.2 to 10.0)
Turkey	4 536<br>(3 290 to 5 270)	5.4<br>(3.9 to 6.2)	-25.1<br>(-40.6 to 5.8)
United Arab Emirates	423<br>(228 to 610)	4.6<br>(3.1 to 6.0)	-23.1<br>(-51.1 to 18.4)
Yemen	889<br>(610 to 1 225)	4.9<br>(3.3 to 6.5)	-16.1<br>(-40.0 to 42.5)
**South Asia**	**239 791**<br>**(220 244 to 270 634)**	**22.0**<br>**(20.0 to 25.0)**	**-2.7**<br>**(-19.6 to 20.4)**
**South Asia**	**239 791**<br>**(220 244 to 270 634)**	**22.0**<br>**(20.0 to 25.0)**	**-2.7**<br>**(-19.6 to 20.4)**
Bangladesh	6 304<br>(5 349 to 7 374)	5.9<br>(5.1 to 6.8)	-28.9<br>(-48.0 to 0.1)
Bhutan	95<br>(70 to 124)	17.6<br>(13.1 to 22.3)	-22.9<br>(-40.3 to 4.6)
India	221 298<br>(201 395 to 251 201)	25.4<br>(23.0 to 29.2)	-7.4<br>(-23.6 to 15.4)
Nepal	2 911<br>(2 253 to 3 619)	16.4<br>(12.8 to 20.1)	-12.5<br>(-29.5 to 13.6)
Pakistan	9 182<br>(6 111 to 11 763)	8.8<br>(6.0 to 11.2)	-0.3<br>(-31.3 to 41.4)
**Southeast Asia, East Asia, and Oceania**	**193 933**<br>**(158 885 to 209 800)**	**8.9**<br>**(7.4 to 9.6)**	**-3.8**<br>**(-24.8 to 12.1)**
**East Asia**	**140 843**<br>**(107 347 to 155 273)**	**8.5**<br>**(6.6 to 9.3)**	**12.1**<br>**(-23.6 to 32.2)**
China	134 773<br>(102 016 to 148 952)	8.6<br>(6.7 to 9.5)	12.8<br>(-23.9 to 33.4)
North Korea	1 757<br>(1 355 to 2 219)	6.7<br>(5.2 to 8.3)	28.5<br>(-5.1 to 68.0)
Taiwan (Province of China)	2 045<br>(1 921 to 2 182)	5.7<br>(5.3 to 6.0)	-26.7<br>(-31.8 to -21.2)
**Oceania**	**506**<br>**(367 to 661)**	**7.5**<br>**(6.0 to 9.0)**	**12.7**<br>**(-7.0 to 33.9)**
American Samoa	2<br>(1 to 2)	4.8<br>(4.2 to 5.4)	5.7<br>(-13.7 to 29.8)
Federated States of Micronesia	4<br>(3 to 5)	6.1<br>(4.7 to 7.4)	11.9<br>(-16.6 to 45.6)
Fiji	21<br>(18 to 24)	3.4<br>(3.0 to 3.9)	7.4<br>(-13.9 to 42.0)
Guam	7<br>(6 to 8)	4.6<br>(4.0 to 5.2)	26.8<br>(-0.6 to 57.4)
Kiribati	2<br>(1 to 2)	2.4<br>(1.9 to 2.9)	-0.8<br>(-18.0 to 19.4)
Marshall Islands	2<br>(1 to 3)	7.0<br>(5.6 to 8.6)	16.9<br>(-9.0 to 47.1)
Northern Mariana Islands	2<br>(2 to 2)	5.2<br>(4.4 to 5.9)	4.2<br>(-22.9 to 32.0)

Location	Mortality (95% UI)		
	2017 counts	2017 age-standardised rates per 100,000	Percentage change in age-standardised rates between 1990 and 2017
Papua New Guinea	317<br>(193 to 460)	6.5<br>(4.4 to 8.5)	20.3<br>(-11.8 to 51.7)
Samoa	7<br>(5 to 8)	5.4<br>(4.2 to 6.7)	24.4<br>(-4.9 to 59.3)
Solomon Islands	101<br>(85 to 119)	31.2<br>(27.0 to 35.4)	-11.5<br>(-28.5 to 8.1)
Tonga	4<br>(3 to 4)	5.3<br>(4.3 to 6.3)	-5.8<br>(-29.3 to 23.0)
Vanuatu	10<br>(7 to 13)	6.2<br>(4.5 to 8.1)	22.6<br>(-11.6 to 60.4)
**Southeast Asia**	**52 585<br>(49 148 to 57 098)**	**10.5<br>(9.8 to 11.3)**	**-29.8<br>(-39.8 to -13.0)**
Cambodia	1 681<br>(1 452 to 1 944)	17.5<br>(15.3 to 20.1)	-6.4<br>(-23.7 to 17.9)
Indonesia	18 244<br>(16 924 to 20 366)	10.9<br>(10.1 to 12.0)	-44.8<br>(-54.6 to -28.5)
Laos	406<br>(311 to 508)	9.9<br>(7.7 to 12.4)	5.5<br>(-28.6 to 39.9)
Malaysia	831<br>(626 to 978)	3.8<br>(2.9 to 4.5)	-18.3<br>(-36.6 to 14.4)
Maldives	15<br>(13 to 17)	5.0<br>(4.4 to 5.7)	-44.2<br>(-58.7 to -8.6)
Mauritius	65<br>(59 to 72)	4.4<br>(3.9 to 4.8)	31.5<br>(17.8 to 45.4)
Myanmar	6 872<br>(5 953 to 7 929)	17.4<br>(15.3 to 19.9)	-15.7<br>(-32.6 to 7.9)
Philippines	3 921<br>(3 478 to 4 411)	6.2<br>(5.5 to 6.9)	57.5<br>(39.7 to 77.6)
Sri Lanka	2 147<br>(1 783 to 2 568)	10.4<br>(8.7 to 12.3)	-3.3<br>(-27.4 to 24.6)
Seychelles	6<br>(5 to 7)	6.2<br>(5.2 to 7.1)	-3.6<br>(-21.4 to 13.8)
Thailand	3 602<br>(3 161 to 4 143)	4.0<br>(3.5 to 4.6)	-50.6<br>(-60.1 to -32.0)
Timor-Leste	63<br>(47 to 77)	8.4<br>(6.7 to 10.2)	18.6<br>(-15.9 to 60.4)
Vietnam	14 662<br>(12 524 to 17 136)	18.0<br>(15.4 to 21.0)	-2.7<br>(-26.5 to 29.4)
**Sub-Saharan Africa**	**38 086<br>(34 089 to 44 273)**	**9.5<br>(8.6 to 11.1)**	**-14.1<br>(-24.6 to 0.0)**
**Central sub-Saharan Africa**	**3 387<br>(2 742 to 4 773)**	**7.5<br>(6.2 to 9.7)**	**-8.1<br>(-23.9 to 13.9)**
Angola	855<br>(677 to 1 174)	9.2<br>(7.3 to 12.3)	-10.9<br>(-32.5 to 20.3)
Central African Republic	146<br>(94 to 249)	7.9<br>(5.9 to 11.2)	-10.5<br>(-33.5 to 12.6)
Congo (Brazzaville)	168<br>(110 to 257)	8.1<br>(5.3 to 12.1)	-16.9<br>(-39.9 to 11.8)
DR Congo	2 111<br>(1 564 to 3 049)	7.0<br>(5.4 to 9.1)	-5.4<br>(-26.2 to 20.5)
Equatorial Guinea	33<br>(20 to 53)	7.5<br>(4.8 to 11.8)	-18.8<br>(-49.6 to 30.2)
Gabon	75<br>(54 to 109)	8.3<br>(6.0 to 11.7)	-20.0<br>(-44.2 to 7.5)
**Eastern sub-Saharan Africa**	**16 221<br>(14 760 to 17 956)**	**12.2<br>(11.2 to 13.5)**	**-16.9<br>(-28.4 to -2.3)**
Burundi	389<br>(305 to 487)	12.0<br>(9.6 to 14.7)	-26.2<br>(-40.8 to -1.1)
Comoros	35<br>(28 to 45)	9.6<br>(7.8 to 12.4)	-21.4<br>(-38.7 to -0.2)
Djibouti	41<br>(29 to 58)	10.0<br>(7.4 to 13.6)	-18.1<br>(-40.8 to 8.2)
Eritrea	207<br>(153 to 282)	11.6<br>(8.8 to 15.1)	-16.4<br>(-37.1 to 10.9)

Location	Mortality (95% UI)		
	**2017 counts**	**2017 age-standardised rates per 100,000**	**Percentage change in age-standardised rates between 1990 and 2017**
Ethiopia	4 461<br>(3 845 to 5 132)	13.1<br>(11.5 to 14.9)	-27.5<br>(-42.9 to -3.3)
Kenya	2 125<br>(1 898 to 2 485)	12.7<br>(11.3 to 14.5)	-8.2<br>(-33.4 to 15.7)
Madagascar	726<br>(564 to 939)	8.6<br>(6.8 to 10.9)	-22.8<br>(-35.3 to -8.1)
Malawi	923<br>(797 to 1 077)	13.2<br>(11.4 to 15.4)	-14.5<br>(-31.2 to 20.0)
Mozambique	1 471<br>(1 085 to 1 821)	15.5<br>(12.1 to 19.0)	-7.4<br>(-29.4 to 25.4)
Rwanda	499<br>(404 to 629)	11.1<br>(9.1 to 13.8)	-31.8<br>(-46.7 to -5.7)
Somalia	500<br>(363 to 694)	10.3<br>(7.8 to 13.6)	-10.8<br>(-31.4 to 22.0)
South Sudan	346<br>(261 to 466)	10.8<br>(7.9 to 14.4)	-10.7<br>(-32.0 to 21.5)
Tanzania	2 585<br>(2 240 to 3 029)	11.5<br>(9.8 to 13.8)	-12.0<br>(-26.8 to 9.2)
Uganda	1 094<br>(846 to 1 351)	9.3<br>(7.4 to 11.3)	-14.5<br>(-39.0 to 16.4)
Zambia	811<br>(701 to 933)	14.7<br>(12.7 to 17.0)	-18.9<br>(-36.3 to 0.2)
**Southern sub-Saharan Africa**	**1 569<br>(1 461 to 1 796)**	**3.1<br>(2.9 to 3.6)**	**-20.6<br>(-36.9 to -2.0)**
Botswana	44<br>(34 to 56)	3.7<br>(3.0 to 4.6)	-22.2<br>(-40.7 to 5.3)
Lesotho	54<br>(41 to 68)	5.1<br>(3.9 to 6.3)	-2.1<br>(-35.7 to 38.2)
Namibia	57<br>(49 to 68)	4.3<br>(3.7 to 5.1)	-23.5<br>(-38.7 to -2.9)
South Africa	1 036<br>(933 to 1 186)	2.6<br>(2.3 to 2.9)	-25.4<br>(-38.9 to -5.1)
Swaziland	22<br>(17 to 28)	4.4<br>(3.4 to 5.5)	-20.0<br>(-42.2 to 8.5)
Zimbabwe	356<br>(262 to 455)	6.7<br>(5.0 to 8.7)	8.0<br>(-22.0 to 34.7)
**Western sub-Saharan Africa**	**16 911<br>(14 329 to 20 729)**	**10.2<br>(8.6 to 12.4)**	**-14.4<br>(-26.5 to 3.3)**
Benin	411<br>(330 to 501)	9.7<br>(7.9 to 11.8)	-19.0<br>(-34.7 to 0.9)
Burkina Faso	1 249<br>(1 081 to 1 438)	15.7<br>(13.8 to 17.7)	-12.7<br>(-25.9 to 6.1)
Cameroon	1 164<br>(933 to 1 429)	11.8<br>(9.6 to 14.5)	-18.0<br>(-34.4 to 1.4)
Cape Verde	11<br>(9 to 13)	2.2<br>(1.9 to 2.7)	-35.1<br>(-48.2 to -17.3)
Chad	592<br>(452 to 792)	11.0<br>(8.1 to 15.2)	-1.9<br>(-20.0 to 23.4)
Cote d'Ivoire	984<br>(792 to 1 248)	11.3<br>(9.1 to 14.2)	-12.9<br>(-31.6 to 8.9)
The Gambia	129<br>(106 to 155)	15.4<br>(12.9 to 18.3)	-2.4<br>(-27.5 to 23.0)
Ghana	1 865<br>(1 580 to 2 141)	15.0<br>(12.9 to 17.2)	14.0<br>(-10.8 to 49.6)
Guinea	554<br>(461 to 661)	11.0<br>(9.1 to 13.1)	-9.3<br>(-32.1 to 17.3)
Guinea-Bissau	68<br>(55 to 89)	11.2<br>(9.4 to 13.8)	-24.5<br>(-39.5 to -5.3)
Liberia	152<br>(115 to 193)	8.9<br>(6.9 to 11.0)	-26.6<br>(-41.4 to -5.8)
Mali	709<br>(546 to 977)	9.1<br>(7.0 to 12.8)	-22.8<br>(-36.6 to -2.2)
Mauritania	156<br>(121 to 206)	8.8<br>(7.0 to 11.5)	-23.6<br>(-39.6 to -1.0)

Location	Mortality (95% UI)		
	**2017 counts**	**2017 age-standardised rates per 100,000**	**Percentage change in age-standardised rates between 1990 and 2017**
Niger	599 (430 to 884)	9.1 (6.4 to 13.7)	-22.5 (-36.3 to -3.1)
Nigeria	7 018 (5 103 to 9 772)	8.7 (6.3 to 12.1)	-21.2 (-41.4 to 7.2)
Sao Tome and Principe	14 (10 to 19)	13.4 (8.6 to 18.8)	9.9 (-23.5 to 49.1)
Senegal	662 (518 to 805)	10.3 (8.3 to 12.3)	-14.1 (-28.4 to 5.1)
Sierra Leone	323 (264 to 400)	10.5 (8.7 to 12.8)	-10.9 (-30.5 to 13.9)
Togo	252 (204 to 316)	9.3 (7.7 to 11.2)	-20.9 (-37.9 to -0.3)
