# Supplementary material for: The global burden of falls: global, regional and national estimates of morbidity and mortality from the Global Burden of Disease Study 2017
Source: Inj Prev. 2020 Jan 15;26(Suppl 1):i3–i11. doi: 10.1136/injuryprev-2019-043286 (PMC7571347; doi:10.1136/injuryprev-2019-043286)
Supplement: Supplementary data [file injuryprev-2019-043286supp005.pdf]

Table 3: YLLs, YLDs, and DALYs for 2017 and percentage change of age-standardised rates between 1990 and 2017 by location for falls

Location	YLLs (95% UI)			YLDs (95% UI)			DALYs (95% UI)		
	2017 counts	2017 age-standardised rates per 100,000	Percentage change in age-standardised rates between 1990 and 2017	2017 counts	2017 age-standardised rates per 100,000	Percentage change in age-standardised rates between 1990 and 2017	2017 counts	2017 age-standardised rates per 100,000	Percentage change in age-standardised rates between 1990 and 2017
**Global**	**16 688 088 (15 101 897 to 17 636 830)**	**217 (196 to 229)**	**-18.5 (-31.7 to -6.2)**	**19 252 699 (13 725 429 to 26 140 433)**	**243 (173 to 330)**	**-9.3 (-10.7 to -7.9)**	**35 940 787 (30 185 695 to 42 903 289)**	**459 (387 to 547)**	**-13.9 (-21.3 to -8.0)**
**Low SDI**	**3 035 204 (2 776 524 to 3 310 498)**	**319 (295 to 353)**	**-17.7 (-31.5 to 1.7)**	**1 708 842 (1 237 007 to 2 286 036)**	**185 (135 to 247)**	**9.9 (7.6 to 12.1)**	**4 744 046 (4 178 135 to 5 354 913)**	**504 (448 to 567)**	**-9.4 (-20.8 to 4.7)**
**Low-middle SDI**	**4 322 097 (3 909 061 to 4 707 961)**	**320 (293 to 352)**	**-16.1 (-26.6 to -0.4)**	**2 470 809 (1 785 368 to 3 315 556)**	**175 (127 to 234)**	**15.0 (12.0 to 18.2)**	**6 792 906 (6 035 771 to 7 657 744)**	**495 (444 to 556)**	**-7.2 (-16.3 to 4.7)**
**Middle SDI**	**4 783 212 (4 047 237 to 5 182 512)**	**228 (195 to 247)**	**-20.3 (-36.4 to -8.6)**	**3 184 082 (2 283 323 to 4 318 877)**	**143 (103 to 193)**	**21.4 (16.1 to 26.7)**	**7 967 294 (6 753 806 to 9 216 910)**	**372 (317 to 428)**	**-8.1 (-20.8 to 0.8)**
**High-middle SDI**	**2 548 533 (2 312 949 to 2 700 612)**	**165 (150 to 175)**	**-25.7 (-40.3 to -15.9)**	**5 051 300 (3 573 300 to 6 952 811)**	**305 (216 to 419)**	**-16.1 (-18.3 to -14.0)**	**7 599 833 (6 063 448 to 9 610 574)**	**470 (379 to 589)**	**-19.8 (-26.5 to -16.0)**
**High SDI**	**1 937 067 (1 884 198 to 1 989 251)**	**103 (100 to 106)**	**-29.4 (-31.5 to -27.3)**	**6 784 835 (4 820 996 to 9 259 655)**	**419 (296 to 576)**	**-8.2 (-9.6 to -6.9)**	**8 721 903 (6 780 201 to 11 177 346)**	**522 (400 to 678)**	**-13.3 (-15.3 to -11.7)**
**Central Europe, Eastern Europe, and Central Asia**	**966 579 (928 588 to 997 962)**	**197 (189 to 204)**	**-28.5 (-31.1 to -25.8)**	**4 022 519 (2 840 390 to 5 582 122)**	**771 (542 to 1 067)**	**-8.4 (-10.5 to -6.2)**	**4 989 097 (3 805 499 to 6 529 439)**	**969 (742 to 1 257)**	**-13.3 (-15.9 to -11.2)**
**Central Asia**	**122 416 (113 998 to 134 465)**	**136 (126 to 149)**	**-46.1 (-50.2 to -40.3)**	**418 376 (295 878 to 574 794)**	**479 (339 to 658)**	**-11.8 (-14.2 to -9.2)**	**540 792 (417 563 to 695 406)**	**615 (475 to 792)**	**-22.7 (-26.5 to -19.2)**
Armenia	2 276 (2 119 to 2 433)	69 (64 to 74)	-76.8 (-79.0 to -74.2)	16 372 (11 540 to 22 780)	454 (320 to 630)	-24.9 (-28.0 to -22.0)	18 649 (13 794 to 25 043)	523 (390 to 699)	-41.9 (-47.0 to -37.3)
Azerbaijan	10 720 (8 267 to 16 408)	108 (84 to 160)	-46.0 (-59.6 to -12.8)	49 940 (35 164 to 68 858)	470 (333 to 646)	-6.3 (-10.2 to -2.3)	60 660 (45 508 to 79 483)	578 (435 to 757)	-17.7 (-24.8 to -7.8)
Georgia	7 014 (6 504 to 7 513)	164 (151 to 177)	-40.2 (-46.2 to -33.2)	21 028 (14 874 to 28 703)	442 (312 to 604)	-20.7 (-22.6 to -18.7)	28 042 (21 907 to 35 656)	606 (475 to 765)	-27.1 (-30.5 to -24.1)
Kazakhstan	32 612 (29 134 to 36 265)	177 (158 to 196)	-30.1 (-37.9 to -22.0)	90 542 (63 918 to 124 290)	500 (353 to 689)	-9.1 (-12.4 to -6.1)	123 154 (95 768 to 157 645)	677 (526 to 866)	-15.7 (-19.7 to -12.1)
Kyrgyzstan	5 903 (5 361 to 6 546)	96 (87 to 106)	-72.3 (-75.2 to -69.1)	25 349 (17 964 to 34 850)	445 (316 to 609)	-26.0 (-28.3 to -23.6)	31 252 (24 081 to 40 499)	541 (414 to 704)	-42.9 (-47.4 to -38.7)
Mongolia	9 883 (7 327 to 12 349)	294 (220 to 365)	-2.5 (-29.1 to 25.4)	19 030 (13 653 to 25 831)	628 (450 to 853)	0.1 (-3.5 to 3.9)	28 914 (22 406 to 36 248)	923 (712 to 1 158)	-0.8 (-11.3 to 8.0)
Tajikistan	16 810 (14 316 to 21 166)	176 (152 to 221)	-39.7 (-50.9 to -15.5)	39 965 (28 630 to 54 347)	527 (378 to 718)	-17.0 (-19.7 to -14.6)	56 775 (44 682 to 70 704)	704 (548 to 887)	-24.2 (-29.5 to -16.2)
Turkmenistan	4 608 (4 030 to 5 237)	94 (83 to 106)	-49.6 (-56.7 to -41.9)	22 089 (15 572 to 30 456)	470 (333 to 645)	-7.0 (-10.6 to -3.3)	26 697 (20 143 to 34 644)	564 (424 to 736)	-18.5 (-23.5 to -13.9)
Uzbekistan	32 589 (28 580 to 36 836)	105 (93 to 119)	-53.4 (-59.6 to -46.5)	134 060 (94 595 to 185 084)	453 (320 to 621)	-8.8 (-11.9 to -5.4)	166 649 (127 974 to 217 536)	558 (427 to 724)	-22.7 (-27.5 to -18.2)
**Central Europe**	**269 086 (259 919 to 278 513)**	**159 (153 to 165)**	**-47.7 (-49.9 to -45.4)**	**1 622 149 (1 145 319 to 2 244 576)**	**1 015 (713 to 1 405)**	**-10.6 (-13.0 to -8.3)**	**1 891 234 (1 413 934 to 2 508 615)**	**1 174 (875 to 1 559)**	**-18.4 (-21.8 to -15.4)**
Albania	2 160 (1 692 to 2 623)	69 (54 to 85)	-15.7 (-33.6 to 5.0)	30 503 (21 598 to 42 606)	895 (632 to 1 245)	8.1 (3.0 to 13.1)	32 663 (23 547 to 44 598)	965 (694 to 1 316)	5.9 (0.6 to 10.8)
Bosnia and Herzegovina	2 589 (2 268 to 2 903)	55 (48 to 61)	-23.9 (-40.8 to 1.8)	40 939 (28 870 to 56 486)	913 (640 to 1 267)	8.4 (3.4 to 13.2)	43 527 (31 409 to 59 203)	968 (695 to 1 319)	5.8 (0.8 to 10.7)
Bulgaria	12 973 (11 906 to 14 047)	143 (131 to 156)	-38.7 (-44.1 to -32.9)	94 477 (66 595 to 131 476)	931 (653 to 1 283)	-11.7 (-14.0 to -9.5)	107 450 (79 183 to 144 455)	1 074 (796 to 1 426)	-16.6 (-19.5 to -14.0)
Croatia	14 195 (13 163 to 15 147)	173 (161 to 185)	-2.3 (-9.8 to 5.4)	51 864 (36 803 to 71 016)	778 (550 to 1 069)	-5.2 (-8.3 to -2.2)	66 060 (50 919 to 85 088)	951 (723 to 1 236)	-4.7 (-7.7 to -1.7)
Czech Republic	21 769 (20 123 to 23 408)	126 (116 to 136)	-67.7 (-70.3 to -64.9)	177 369 (125 355 to 244 115)	1 192 (843 to 1 650)	-4.2 (-6.9 to -1.4)	199 139 (146 807 to 265 562)	1 318 (969 to 1 775)	-19.4 (-24.3 to -15.2)
Hungary	28 011 (26 292 to 29 821)	161 (150 to 171)	-68.1 (-70.3 to -65.8)	144 988 (102 398 to 200 260)	1 024 (724 to 1 422)	-19.2 (-22.3 to -16.3)	173 000 (130 137 to 227 092)	1 185 (884 to 1 578)	-33.1 (-37.6 to -28.8)
Macedonia	1 961 (1 674 to 2 193)	72 (60 to 80)	19.1 (-20.0 to 50.5)	25 157 (17 722 to 35 055)	915 (646 to 1 269)	12.6 (8.6 to 16.7)	27 119 (19 675 to 36 957)	987 (718 to 1 343)	13.1 (7.1 to 17.5)
Montenegro	567 (486 to 668)	69 (59 to 82)	-27.9 (-39.9 to -12.6)	7 194 (5 075 to 9 994)	914 (643 to 1 274)	13.6 (11.2 to 15.9)	7 761 (5 604 to 10 539)	983 (707 to 1 344)	9.2 (5.6 to 12.4)
Poland	105 048 (97 365 to 113 222)	188 (174 to 203)	-34.6 (-40.1 to -29.1)	560 199 (395 305 to 774 489)	1 051 (740 to 1 455)	-7.6 (-10.7 to -4.5)	665 246 (499 321 to 875 365)	1 239 (929 to 1 631)	-13.0 (-16.7 to -9.8)
Romania	46 331 (43 140 to 49 665)	180 (168 to 193)	-52.0 (-55.5 to -48.4)	265 040 (186 496 to 367 238)	975 (681 to 1 350)	-26.1 (-28.9 to -23.4)	311 371 (232 675 to 412 957)	1 155 (865 to 1 528)	-31.8 (-35.0 to -28.9)
Serbia	10 800 (8 756 to 12 191)	81 (66 to 92)	-16.5 (-34.9 to 1.7)	106 672 (75 506 to 147 982)	914 (642 to 1 266)	14.1 (10.8 to 17.4)	117 473 (85 851 to 159 741)	996 (724 to 1 354)	10.8 (6.6 to 14.9)
Slovakia	15 786 (12 875 to 18 267)	208 (171 to 241)	-46.5 (-55.0 to -31.5)	77 917 (55 159 to 108 045)	1 069 (753 to 1 480)	-12.2 (-14.4 to -10.2)	93 703 (70 102 to 124 080)	1 277 (952 to 1 695)	-20.5 (-25.1 to -16.0)
Slovenia	6 895 (6 331 to 7 444)	182 (167 to 196)	-45.1 (-50.0 to -40.1)	39 828 (28 331 to 54 481)	1 279 (908 to 1 768)	3.9 (1.8 to 6.0)	46 724 (35 178 to 61 455)	1 461 (1 088 to 1 948)	-6.5 (-10.1 to -3.4)
**Eastern Europe**	**575 077 (549 117 to 596 079)**	**231 (220 to 240)**	**-8.6 (-12.6 to -5.3)**	**1 981 994 (1 388 116 to 2 749 579)**	**733 (514 to 1 013)**	**-2.8 (-5.2 to -0.4)**	**2 557 071 (1 966 184 to 3 316 952)**	**964 (747 to 1 245)**	**-4.3 (-6.4 to -2.2)**
Belarus	31 047 (27 689 to 34 297)	267 (237 to 295)	-11.4 (-21.5 to -1.1)	94 968 (66 861 to 131 753)	759 (533 to 1 047)	-4.9 (-7.5 to -2.4)	126 015 (98 278 to 162 801)	1 025 (803 to 1 311)	-6.7 (-9.9 to -3.5)
Estonia	2 975 (2 537 to 3 449)	173 (147 to 201)	-59.3 (-65.6 to -52.0)	13 355 (9 379 to 18 546)	734 (516 to 1 016)	-20.9 (-23.1 to -18.5)	16 330 (12 325 to 21 395)	907 (685 to 1 187)	-32.9 (-37.4 to -29.4)
Latvia	6 029 (5 190 to 6 893)	228 (196 to 262)	-52.8 (-59.4 to -45.8)	20 854 (14 724 to 28 890)	752 (527 to 1 046)	-23.6 (-26.0 to -21.2)	26 883 (20 778 to 34 581)	981 (759 to 1 260)	-33.2 (-37.2 to -29.8)
Lithuania	11 582 (10 646 to 12 561)	288 (264 to 314)	-33.2 (-38.9 to -26.4)	33 622 (23 682 to 46 493)	809 (568 to 1 120)	-10.2 (-12.7 to -7.8)	45 204 (35 135 to 57 887)	1 098 (855 to 1 402)	-17.6 (-21.2 to -14.4)
Moldova	7 875 (7 291 to 8 492)	172 (159 to 185)	-52.3 (-56.4 to -47.8)	30 864 (21 916 to 42 377)	665 (473 to 912)	-19.7 (-22.3 to -17.0)	38 739 (29 724 to 50 146)	837 (640 to 1 081)	-29.6 (-33.3 to -26.4)
Russian Federation	393 675 (378 251 to 405 484)	229 (219 to 236)	5.6 (-1.6 to 9.3)	1 375 571 (966 580 to 1 906 872)	740 (519 to 1 023)	2.9 (0.2 to 5.5)	1 769 246 (1 360 426 to 2 297 059)	968 (747 to 1 252)	3.5 (1.1 to 5.7)
Ukraine	121 893 (112 493 to 132 443)	235 (217 to 256)	-24.2 (-30.8 to -16.9)	412 760 (289 234 to 575 017)	705 (493 to 979)	-13.0 (-15.6 to -10.5)	534 653 (409 488 to 698 091)	940 (730 to 1 216)	-16.1 (-19.2 to -13.2)
**High-income**	**1 705 180 (1 655 481 to 1 749 551)**	**95 (92 to 97)**	**-26.4 (-28.8 to -24.4)**	**5 840 033 (4 153 445 to 7 965 892)**	**385 (272 to 529)**	**-8.5 (-10.2 to -6.9)**	**7 545 214 (5 876 493 to 9 658 549)**	**479 (368 to 620)**	**-12.7 (-14.6 to -11.2)**
**Australasia**	**40 521 (37 201 to 44 017)**	**90 (82 to 97)**	**4.5 (-4.6 to 14.3)**	**261 552 (184 830 to 362 585)**	**713 (502 to 994)**	**23.1 (20.7 to 25.6)**	**302 072 (225 902 to 401 819)**	**802 (590 to 1 081)**	**20.7 (17.9 to 23.2)**
Australia	34 091 (30 739 to 37 609)	88 (79 to 97)	10.7 (-1.4 to 23.0)	214 068 (151 387 to 296 609)	693 (488 to 966)	24.3 (21.7 to 27.0)	248 159 (185 581 to 330 409)	781 (575 to 1 051)	22.6 (19.7 to 25.3)
New Zealand	6 429 (6 000 to 6 899)	97 (91 to 104)	-15.0 (-21.1 to -8.5)	47 484 (33 598 to 65 562)	820 (580 to 1 142)	19.4 (14.7 to 24.4)	53 913 (40 044 to 71 798)	917 (677 to 1 238)	14.4 (9.9 to 18.8)
**High-income Asia-Pacific**	**235 605 (222 969 to 248 489)**	**74 (69 to 78)**	**-42.8 (-46.3 to -38.7)**	**1 306 146 (931 659 to 1 779 010)**	**481 (341 to 654)**	**10.6 (8.5 to 12.8)**	**1 541 751 (1 172 479 to 2 016 435)**	**554 (416 to 729)**	**-1.6 (-5.6 to 1.8)**
Brunei	725 (613 to 824)	175 (152 to 196)	-16.3 (-29.4 to 2.0)	1 956 (1 370 to 2 707)	468 (328 to 653)	-0.6 (-3.7 to 2.3)	2 681 (2 076 to 3 463)	642 (498 to 819)	-5.4 (-11.6 to 0.7)
Japan	153 654 (146 734 to 161 071)	60 (57 to 63)	-30.4 (-33.8 to -26.7)	992 975 (711 271 to 1 343 552)	513 (366 to 694)	26.2 (23.6 to 29.6)	1 146 629 (863 275 to 1 500 191)	573 (427 to 754)	16.3 (12.5 to 19.7)
South Korea	76 952 (69 091 to 84 788)	108 (97 to 118)	-55.3 (-60.0 to -50.3)	283 072 (199 131 to 391 660)	418 (293 to 577)	-19.1 (-21.9 to -16.4)	360 024 (278 774 to 467 156)	526 (405 to 683)	-30.6 (-34.7 to -27.0)
Singapore	4 274 (3 901 to 4 700)	69 (63 to 76)	-39.8 (-45.3 to -33.8)	28 143 (19 805 to 38 911)	434 (305 to 600)	4.4 (1.5 to 6.9)	32 417 (24 114 to 43 077)	503 (376 to 670)	-5.1 (-9.6 to -1.9)
**High-income North America**	**599 913 (584 935 to 615 363)**	**109 (106 to 112)**	**22.3 (18.2 to 25.7)**	**1 577 676 (1 116 030 to 2 139 319)**	**311 (221 to 419)**	**-29.3 (-33.9 to -24.9)**	**2 177 589 (1 712 659 to 2 746 243)**	**421 (332 to 530)**	**-20.6 (-25.3 to -16.0)**
Canada	66 502 (61 915 to 71 689)	108 (101 to 116)	5.8 (-1.7 to 14.5)	157 562 (110 895 to 216 847)	298 (210 to 413)	8.7 (6.5 to 10.9)	224 064 (177 868 to 283 351)	406 (317 to 518)	7.9 (5.3 to 10.7)
Greenland	190 (170 to 214)	310 (277 to 347)	-34.4 (-45.8 to -15.5)	254 (180 to 349)	395 (280 to 540)	-31.1 (-33.2 to -29.2)	444 (368 to 542)	705 (586 to 854)	-32.6 (-38.2 to -25.0)
USA	533 210 (518 637 to 546 456)	109 (106 to 112)	23.9 (19.6 to 27.6)	1 419 832 (1 005 157 to 1 926 893)	313 (223 to 422)	-31.7 (-36.4 to -27.2)	1 953 042 (1 535 647 to 2 465 035)	422 (333 to 532)	-22.8 (-27.6 to -17.9)
**Southern Latin America**	**59 531 (54 838 to 64 697)**	**80 (73 to 87)**	**-31.2 (-37.2 to -24.3)**	**224 284 (159 020 to 308 056)**	**304 (215 to 417)**	**-15.3 (-18.0 to -12.5)**	**283 815 (217 150 to 365 468)**	**384 (293 to 495)**	**-19.2 (-22.3 to -16.1)**
Argentina	33 078 (29 399 to 37 307)	68 (60 to 76)	-39.3 (-46.5 to -31.4)	152 318 (107 701 to 208 266)	314 (223 to 430)	-11.8 (-15.0 to -8.6)	185 395 (140 141 to 240 554)	381 (289 to 495)	-18.4 (-22.3 to -14.9)
Chile	22 363 (19 843 to 25 050)	106 (94 to 119)	-17.0 (-26.9 to -6.7)	58 448 (41 185 to 80 108)	279 (196 to 381)	-25.6 (-28.6 to -22.5)	80 811 (62 877 to 101 891)	384 (299 to 486)	-23.4 (-27.0 to -19.7)
Uruguay	4 087 (3 614 to 4 575)	91 (80 to 102)	-23.1 (-32.5 to -12.1)	13 509 (9 541 to 18 532)	312 (219 to 428)	-6.8 (-9.8 to -3.7)	17 596 (13 571 to 22 589)	402 (310 to 517)	-11.0 (-14.9 to -7.5)
**Western Europe**	**769 612 (736 657 to 801 284)**	**97 (93 to 101)**	**-37.5 (-40.4 to -34.9)**	**2 470 375 (1 753 341 to 3 381 635)**	**399 (281 to 549)**	**0.0 (-0.9 to 1.0)**	**3 239 987 (2 529 527 to 4 139 650)**	**496 (379 to 646)**	**-10.5 (-13.6 to -8.2)**
Andorra	120 (96 to 151)	99 (79 to 126)	-26.4 (-40.4 to -8.3)	465 (328 to 644)	427 (301 to 594)	2.1 (0.3 to 4.2)	585 (439 to 762)	526 (392 to 687)	-4.8 (-10.2 to -0.1)
Austria	19 201 (17 743 to 20 672)	127 (117 to 136)	-49.5 (-53.6 to -45.2)	53 138 (37 624 to 73 371)	424 (298 to 584)	-13.4 (-15.3 to -11.5)	72 339 (56 024 to 92 127)	550 (420 to 707)	-25.6 (-29.5 to -22.6)
Belgium	28 046 (26 047 to 30 076)	141 (131 to 151)	-4.0 (-11.4 to 4.0)	79 498 (56 366 to 109 479)	488 (345 to 676)	15.0 (12.5 to 17.6)	107 544 (84 752 to 137 308)	629 (486 to 813)	10.1 (7.1 to 13.0)
Cyprus	1 515 (1 292 to 1 729)	92 (78 to 105)	-36.1 (-46.9 to -18.1)	6 140 (4 323 to 8 520)	396 (279 to 550)	-3.2 (-5.6 to -1.2)	7 655 (5 796 to 10 083)	488 (368 to 646)	-11.7 (-17.2 to -6.3)
Denmark	8 687 (8 011 to 9 402)	81 (75 to 87)	-61.7 (-64.7 to -58.2)	31 276 (22 226 to 43 040)	402 (285 to 555)	-0.5 (-3.2 to 2.0)	39 963 (31 025 to 51 860)	483 (367 to 632)	-21.5 (-27.0 to -16.9)
Finland	17 948 (16 700 to 19 322)	174 (161 to 188)	-26.4 (-32.2 to -19.5)	40 362 (28 696 to 55 595)	494 (350 to 682)	9.2 (7.1 to 11.4)	58 310 (46 496 to 73 446)	667 (524 to 848)	-3.0 (-6.8 to 0.3)
France	154 543 (142 298 to 165 969)	127 (117 to 137)	-41.4 (-46.5 to -36.8)	407 285 (287 888 to 559 300)	427 (301 to 590)	-4.4 (-6.3 to -2.5)	561 829 (441 226 to 714 224)	554 (427 to 715)	-16.5 (-20.6 to -13.5)
Germany	177 662 (160 111 to 197 657)	107 (96 to 120)	-35.9 (-42.6 to -28.5)	511 389 (361 675 to 703 307)	410 (289 to 567)	2.4 (0.3 to 4.5)	689 051 (538 845 to 879 284)	518 (394 to 671)	-8.9 (-13.0 to -5.6)
Greece	11 410 (10 650 to 12 258)	71 (66 to 76)	-44.1 (-48.2 to -39.7)	55 019 (38 710 to 76 317)	373 (262 to 515)	-4.9 (-6.9 to -2.7)	66 430 (49 871 to 87 527)	444 (332 to 588)	-14.5 (-18.1 to -11.7)
Iceland	478 (450 to 511)	101 (95 to 108)	-17.9 (-24.5 to -10.8)	1 723 (1 223 to 2 374)	411 (290 to 566)	5.1 (3.0 to 7.3)	2 201 (1 704 to 2 848)	512 (393 to 667)	-0.5 (-3.5 to 2.1)
Ireland	5 162 (4 686 to 5 640)	81 (73 to 88)	-32.7 (-39.6 to -25.4)	23 258 (16 423 to 31 877)	400 (281 to 553)	5.6 (3.1 to 8.3)	28 421 (21 574 to 36 933)	481 (363 to 627)	-3.6 (-7.5 to -0.5)
Israel	5 100 (4 708 to 5 535)	48 (44 to 53)	-33.4 (-39.0 to -27.1)	37 177 (26 202 to 51 190)	387 (272 to 533)	9.3 (6.4 to 12.1)	42 278 (31 241 to 56 196)	436 (321 to 582)	2.0 (-1.8 to 5.4)
Italy	84 348 (78 228 to 90 676)	68 (63 to 73)	-50.9 (-54.7 to -46.9)	333 836 (237 320 to 455 844)	354 (249 to 487)	-7.8 (-9.9 to -5.5)	418 184 (320 632 to 541 910)	422 (318 to 554)	-19.2 (-23.1 to -16.1)
Luxembourg	1 106 (982 to 1 231)	125 (110 to 138)	-36.8 (-44.5 to -28.3)	3 306 (2 344 to 4 539)	427 (302 to 584)	-7.2 (-9.4 to -5.2)	4 412 (3 449 to 5 636)	552 (428 to 711)	-16.1 (-19.7 to -12.8)
Malta	790 (733 to 847)	113 (105 to 121)	-18.5 (-25.5 to -11.6)	2 780 (1 971 to 3 824)	465 (330 to 643)	13.2 (10.7 to 15.9)	3 570 (2 762 to 4 614)	578 (441 to 756)	5.2 (1.6 to 8.3)
Netherlands	36 534 (34 188 to 39 190)	114 (107 to 122)	18.4 (10.1 to 27.5)	78 505 (55 950 to 108 066)	330 (233 to 458)	3.1 (0.5 to 5.9)	115 039 (91 885 to 144 419)	445 (347 to 571)	6.7 (3.7 to 10.2)
Norway	10 370 (9 892 to 10 842)	113 (108 to 119)	-33.7 (-37.3 to -30.1)	35 855 (25 723 to 48 106)	517 (371 to 701)	0.5 (-0.8 to 1.9)	46 225 (36 105 to 58 433)	631 (485 to 814)	-8.0 (-10.6 to -6.0)
Portugal	12 473 (11 347 to 13 485)	71 (64 to 78)	-63.4 (-67.4 to -59.6)	46 005 (32 566 to 63 250)	293 (207 to 408)	-30.8 (-33.0 to -28.8)	58 478 (44 724 to 75 488)	365 (278 to 474)	-41.1 (-44.4 to -38.5)
Spain	55 431 (51 266 to 59 309)	72 (67 to 78)	-42.9 (-47.6 to -37.8)	241 132 (170 629 to 334 961)	367 (259 to 508)	0.9 (-1.1 to 3.1)	296 563 (225 951 to 389 713)	440 (331 to 583)	-10.4 (-14.4 to -7.5)
Sweden	17 739 (16 673 to 18 842)	90 (85 to 96)	-14.5 (-20.1 to -8.7)	68 382 (48 855 to 91 800)	499 (357 to 679)	13.2 (10.7 to 15.8)	86 121 (66 754 to 109 915)	589 (448 to 768)	7.9 (4.9 to 10.4)
Switzerland	20 674 (19 017 to 22 338)	132 (121 to 143)	-51.5 (-55.9 to -47.1)	52 598 (37 413 to 71 512)	409 (290 to 560)	-25.7 (-27.6 to -23.6)	73 272 (58 036 to 91 959)	541 (422 to 689)	-34.2 (-37.0 to -31.8)

Location	YLLs (95% UI)			YLDs (95% UI)			DALYs (95% UI)		
	2017 counts	2017 age-standardised rates per 100,000	Percentage change in age-standardised rates between 1990 and 2017	2017 counts	2017 age-standardised rates per 100,000	Percentage change in age-standardised rates between 1990 and 2017	2017 counts	2017 age-standardised rates per 100,000	Percentage change in age-standardised rates between 1990 and 2017
United Kingdom	99 475 (97 198 to 101 657)	91 (89 to 93)	-6.6 (-9.2 to -4.4)	358 687 (254 601 to 495 597)	406 (286 to 561)	16.1 (14.6 to 17.4)	458 163 (354 175 to 593 849)	497 (378 to 650)	11.1 (9.2 to 12.6)
**Latin America and Caribbean**	**831 890 (805 313 to 853 332)**	**144 (140 to 148)**	**-31.6 (-33.9 to -29.5)**	**1 243 792 (893 099 to 1 658 404)**	**213 (153 to 283)**	**16.0 (11.8 to 20.3)**	**2 075 683 (1 728 233 to 2 486 729)**	**357 (298 to 427)**	**-9.4 (-14.2 to -5.0)**
**Andean Latin America**	**73 068 (64 005 to 82 028)**	**125 (110 to 140)**	**-22.4 (-36.4 to -9.3)**	**95 766 (68 412 to 130 236)**	**167 (119 to 227)**	**6.3 (1.3 to 11.6)**	**168 834 (138 604 to 202 617)**	**291 (239 to 350)**	**-8.3 (-17.5 to -0.5)**
Bolivia	15 971 (11 671 to 20 732)	157 (118 to 199)	-32.0 (-51.2 to -6.2)	16 734 (12 063 to 22 365)	171 (122 to 229)	3.9 (-0.5 to 8.0)	32 705 (25 922 to 40 004)	328 (260 to 398)	-17.1 (-30.9 to -1.3)
Ecuador	28 705 (25 462 to 32 280)	179 (159 to 201)	-24.9 (-33.3 to -15.3)	29 188 (20 843 to 39 978)	186 (133 to 256)	-4.5 (-9.5 to 0.8)	57 893 (48 905 to 68 161)	365 (308 to 431)	-15.7 (-21.2 to -9.9)
Peru	28 392 (21 118 to 35 155)	88 (66 to 109)	-16.6 (-44.8 to 11.6)	49 844 (35 701 to 67 959)	156 (112 to 212)	13.6 (7.5 to 20.3)	78 236 (61 448 to 97 774)	244 (192 to 305)	0.4 (-15.8 to 14.2)
**Caribbean**	**61 877 (55 871 to 67 608)**	**126 (113 to 138)**	**-7.3 (-14.4 to 0.3)**	**82 026 (58 184 to 111 446)**	**165 (117 to 225)**	**22.1 (19.0 to 25.3)**	**143 904 (118 695 to 173 510)**	**291 (239 to 351)**	**7.3 (1.8 to 12.2)**
Antigua and Barbuda	42 (39 to 46)	45 (41 to 49)	5.6 (-5.4 to 17.4)	135 (96 to 184)	136 (96 to 186)	28.9 (23.9 to 33.6)	177 (139 to 227)	181 (142 to 231)	22.2 (16.4 to 27.6)
The Bahamas	315 (285 to 349)	85 (77 to 93)	2.8 (-8.1 to 15.6)	554 (394 to 760)	144 (102 to 197)	22.5 (18.6 to 26.5)	870 (702 to 1 090)	229 (186 to 285)	14.3 (8.2 to 20.2)
Barbados	187 (171 to 205)	45 (41 to 50)	2.7 (-7.6 to 13.7)	543 (386 to 744)	137 (97 to 187)	28.7 (23.5 to 33.6)	730 (570 to 932)	182 (143 to 233)	21.1 (15.3 to 26.5)
Belize	323 (298 to 350)	99 (92 to 107)	21.8 (5.9 to 36.1)	511 (367 to 689)	157 (112 to 210)	37.4 (32.8 to 42.0)	835 (691 to 1 010)	256 (212 to 309)	30.9 (22.9 to 37.4)
Bermuda	54 (50 to 59)	52 (48 to 58)	-28.6 (-36.2 to -19.8)	143 (100 to 196)	144 (102 to 199)	18.7 (14.3 to 22.5)	197 (154 to 251)	197 (153 to 252)	0.9 (-5.3 to 6.3)
Cuba	32 784 (29 579 to 36 385)	187 (169 to 208)	3.6 (-7.4 to 16.0)	29 238 (20 550 to 40 057)	179 (126 to 246)	18.0 (14.1 to 21.9)	62 022 (52 440 to 73 158)	366 (308 to 434)	10.2 (3.5 to 17.0)
Dominica	63 (57 to 69)	80 (73 to 88)	45.8 (30.2 to 63.0)	118 (84 to 161)	145 (103 to 197)	37.5 (32.7 to 42.3)	181 (146 to 223)	224 (181 to 274)	40.4 (34.1 to 47.1)
Dominican Republic	4 805 (3 829 to 6 164)	48 (39 to 62)	-25.0 (-41.1 to 4.9)	14 658 (10 400 to 19 876)	147 (104 to 199)	22.6 (15.9 to 29.9)	19 463 (14 921 to 24 537)	195 (150 to 246)	5.9 (-3.2 to 16.5)
Grenada	125 (115 to 135)	90 (82 to 98)	-10.4 (-19.2 to -0.7)	208 (148 to 280)	156 (111 to 211)	29.6 (25.4 to 33.7)	332 (271 to 407)	245 (199 to 300)	11.4 (4.5 to 17.0)
Guyana	1 099 (950 to 1 264)	166 (144 to 188)	-2.6 (-17.4 to 12.3)	1 140 (817 to 1 531)	169 (122 to 226)	28.4 (23.7 to 32.8)	2 240 (1 869 to 2 639)	335 (281 to 395)	10.9 (1.5 to 19.7)
Haiti	10 499 (7 098 to 14 401)	118 (83 to 159)	-27.4 (-42.3 to -1.7)	13 646 (9 913 to 18 175)	150 (109 to 198)	22.9 (19.4 to 26.6)	24 145 (18 782 to 30 051)	268 (211 to 333)	-5.8 (-19.5 to 11.3)
Jamaica	1 549 (1 217 to 1 835)	54 (42 to 63)	46.4 (11.2 to 75.7)	4 407 (3 140 to 5 989)	151 (107 to 204)	31.4 (26.6 to 36.1)	5 956 (4 677 to 7 581)	204 (160 to 260)	35.0 (25.8 to 43.5)
Puerto Rico	5 273 (4 850 to 5 716)	96 (89 to 105)	0.2 (-8.9 to 10.3)	9 617 (6 743 to 13 265)	172 (121 to 239)	29.9 (26.1 to 33.6)	14 890 (12 073 to 18 527)	269 (218 to 334)	17.4 (11.8 to 22.5)
Saint Lucia	128 (116 to 139)	64 (59 to 70)	-5.1 (-14.7 to 5.8)	281 (200 to 380)	141 (100 to 190)	26.9 (22.3 to 31.7)	408 (323 to 507)	205 (163 to 255)	14.7 (8.8 to 20.4)
Saint Vincent and the Grenadines	166 (151 to 181)	130 (119 to 143)	32.3 (18.3 to 48.0)	211 (151 to 282)	164 (117 to 219)	43.0 (38.6 to 47.6)	376 (311 to 450)	294 (244 to 352)	38.1 (30.5 to 45.7)
Suriname	599 (532 to 669)	106 (95 to 119)	7.5 (-6.5 to 22.8)	940 (675 to 1 264)	162 (116 to 217)	30.0 (25.7 to 34.6)	1 539 (1 270 to 1 868)	268 (222 to 325)	20.0 (12.5 to 27.0)
Trinidad and Tobago	1 492 (1 201 to 1 824)	99 (80 to 120)	-15.7 (-31.8 to 1.7)	2 512 (1 783 to 3 411)	153 (109 to 208)	18.1 (14.0 to 22.3)	4 003 (3 174 to 4 964)	252 (202 to 310)	2.0 (-7.5 to 11.3)
Virgin Islands	149 (126 to 169)	97 (84 to 111)	3.3 (-14.2 to 21.3)	215 (151 to 295)	146 (103 to 202)	19.4 (14.3 to 24.6)	364 (293 to 451)	243 (197 to 301)	12.4 (2.6 to 21.0)
**Central Latin America**	**311 994 (295 931 to 324 289)**	**127 (121 to 132)**	**-45.5 (-49.1 to -43.1)**	**432 420 (309 703 to 576 521)**	**178 (127 to 237)**	**-2.4 (-6.2 to 1.5)**	**744 415 (622 308 to 885 841)**	**305 (254 to 363)**	**-26.6 (-31.1 to -22.4)**
Colombia	50 422 (43 923 to 57 797)	97 (84 to 111)	-51.7 (-58.1 to -44.3)	63 201 (44 728 to 85 716)	119 (84 to 161)	-16.8 (-20.6 to -12.9)	113 623 (93 753 to 136 539)	216 (179 to 259)	-37.2 (-42.5 to -31.8)
Costa Rica	5 817 (5 135 to 6 361)	118 (104 to 129)	-23.0 (-32.6 to -15.7)	6 682 (4 695 to 9 104)	136 (96 to 186)	2.5 (-1.6 to 7.1)	12 499 (10 283 to 14 874)	254 (209 to 302)	-11.2 (-17.9 to -6.2)
El Salvador	10 798 (8 568 to 13 833)	183 (145 to 232)	-20.3 (-36.2 to 8.8)	8 384 (5 924 to 11 289)	142 (100 to 191)	1.0 (-4.5 to 6.8)	19 182 (15 969 to 23 191)	325 (271 to 391)	-12.2 (-22.7 to 6.1)
Guatemala	25 285 (22 235 to 28 548)	187 (165 to 211)	-34.3 (-42.5 to -25.5)	19 652 (14 091 to 26 266)	149 (107 to 198)	-2.7 (-6.4 to 1.2)	44 937 (38 275 to 52 302)	336 (286 to 390)	-23.3 (-29.7 to -17.2)
Honduras	5 772 (4 482 to 7 612)	77 (58 to 103)	-32.0 (-50.4 to -7.7)	10 016 (7 190 to 13 388)	132 (95 to 176)	11.2 (6.5 to 15.6)	15 788 (12 603 to 19 539)	209 (166 to 257)	-9.8 (-21.2 to 2.8)
Mexico	173 248 (162 372 to 178 651)	143 (135 to 148)	-47.2 (-51.6 to -45.3)	272 504 (195 339 to 361 744)	228 (162 to 302)	3.8 (-0.8 to 8.2)	445 752 (369 909 to 534 652)	371 (308 to 446)	-24.4 (-29.8 to -19.8)
Nicaragua	5 028 (4 307 to 5 871)	95 (81 to 110)	-30.2 (-41.3 to -16.2)	6 742 (4 828 to 9 082)	126 (90 to 169)	4.1 (-0.6 to 9.1)	11 770 (9 661 to 14 209)	221 (181 to 266)	-14.0 (-21.8 to -5.4)
Panama	3 933 (3 502 to 4 355)	100 (89 to 111)	-21.6 (-30.5 to -12.8)	5 046 (3 560 to 6 885)	127 (89 to 173)	3.2 (-1.9 to 7.9)	8 979 (7 443 to 10 750)	227 (188 to 271)	-9.4 (-15.5 to -4.2)
Venezuela	31 692 (27 091 to 37 025)	107 (92 to 125)	-38.5 (-47.5 to -28.0)	40 194 (28 434 to 54 748)	137 (97 to 186)	-8.8 (-13.1 to -4.7)	71 885 (58 075 to 87 477)	244 (198 to 295)	-24.8 (-30.9 to -18.4)
**Tropical Latin America**	**384 951 (369 410 to 395 868)**	**170 (163 to 174)**	**-23.2 (-27.4 to -20.2)**	**633 580 (457 986 to 844 999)**	**270 (195 to 361)**	**33.2 (28.0 to 38.5)**	**1 018 530 (845 849 to 1 226 178)**	**440 (366 to 528)**	**3.8 (-2.0 to 9.4)**
Brazil	378 758 (363 124 to 389 681)	171 (164 to 177)	-23.6 (-27.9 to -20.5)	620 786 (448 970 to 827 987)	272 (196 to 363)	33.8 (28.4 to 39.1)	999 544 (829 821 to 1 202 599)	443 (369 to 532)	3.7 (-2.2 to 9.3)
Paraguay	6 193 (4 735 to 7 588)	107 (82 to 131)	19.8 (-17.5 to 56.9)	12 793 (9 015 to 17 253)	209 (148 to 282)	10.6 (6.1 to 14.9)	18 986 (14 987 to 23 650)	316 (250 to 391)	13.5 (0.2 to 25.8)
**North Africa and Middle East**	**845 178 (737 652 to 930 109)**	**151 (130 to 165)**	**-29.4 (-41.1 to -2.6)**	**891 722 (631 423 to 1 220 846)**	**164 (117 to 224)**	**-11.6 (-15.2 to -8.0)**	**1 736 900 (1 448 630 to 2 074 624)**	**315 (262 to 376)**	**-21.1 (-29.7 to -7.5)**
**North Africa and Middle East**	**845 178 (737 652 to 930 109)**	**151 (130 to 165)**	**-29.4 (-41.1 to -2.6)**	**891 722 (631 423 to 1 220 846)**	**164 (117 to 224)**	**-11.6 (-15.2 to -8.0)**	**1 736 900 (1 448 630 to 2 074 624)**	**315 (262 to 376)**	**-21.1 (-29.7 to -7.5)**
Afghanistan	153 728 (122 514 to 192 511)	457 (379 to 552)	-30.3 (-58.3 to 85.3)	45 605 (33 461 to 60 614)	197 (144 to 260)	-3.4 (-5.7 to -1.4)	199 333 (166 288 to 239 257)	654 (558 to 765)	-24.0 (-50.2 to 44.0)
Algeria	49 559 (37 875 to 67 803)	125 (96 to 169)	-26.3 (-41.7 to 2.5)	61 743 (43 718 to 85 355)	159 (113 to 220)	-15.5 (-19.5 to -11.6)	111 302 (88 342 to 140 831)	284 (226 to 358)	-20.6 (-29.0 to -8.1)
Bahrain	721 (473 to 930)	48 (35 to 59)	-36.6 (-50.4 to -10.3)	2 483 (1 750 to 3 402)	158 (112 to 217)	-4.1 (-8.7 to 0.8)	3 204 (2 424 to 4 226)	206 (158 to 268)	-14.3 (-21.4 to -5.8)
Egypt	124 914 (92 504 to 156 122)	142 (104 to 180)	-26.4 (-41.7 to 3.1)	122 849 (86 980 to 167 786)	149 (106 to 203)	-9.8 (-14.5 to -5.1)	247 763 (196 727 to 300 405)	291 (228 to 354)	-18.7 (-29.5 to -4.0)
Iran	96 728 (86 702 to 113 091)	123 (110 to 142)	-39.5 (-48.4 to -18.5)	139 116 (98 188 to 192 417)	168 (119 to 233)	-7.8 (-11.9 to -3.8)	235 844 (190 975 to 290 941)	292 (238 to 359)	-24.6 (-31.6 to -13.8)
Iraq	15 749 (13 236 to 20 527)	40 (35 to 50)	-62.7 (-74.7 to -34.3)	60 091 (43 196 to 80 640)	168 (121 to 225)	17.4 (14.0 to 20.8)	75 840 (58 724 to 95 554)	208 (161 to 264)	-16.9 (-31.9 to -0.4)
Jordan	7 870 (6 592 to 9 366)	79 (68 to 93)	-43.4 (-57.2 to -19.2)	12 727 (8 955 to 17 533)	140 (99 to 193)	-11.5 (-15.9 to -7.4)	20 597 (16 593 to 25 733)	219 (176 to 273)	-26.5 (-36.1 to -14.6)
Kuwait	4 319 (3 805 to 4 820)	91 (80 to 100)	-28.8 (-37.0 to -20.2)	7 644 (5 399 to 10 630)	180 (127 to 249)	-8.7 (-11.5 to -5.9)	11 962 (9 667 to 14 896)	271 (218 to 337)	-16.6 (-20.8 to -12.7)
Lebanon	6 856 (3 989 to 14 365)	97 (58 to 179)	-33.3 (-54.9 to -4.1)	11 541 (8 077 to 16 034)	151 (106 to 209)	-5.0 (-9.7 to -0.3)	18 397 (13 314 to 25 882)	248 (181 to 341)	-18.5 (-30.5 to -4.9)
Libya	9 723 (6 104 to 13 107)	148 (95 to 197)	-21.7 (-50.6 to 7.7)	9 877 (6 964 to 13 652)	155 (109 to 214)	-19.6 (-23.4 to -15.8)	19 600 (14 408 to 24 404)	303 (226 to 376)	-20.6 (-37.2 to -6.5)
Morocco	46 783 (35 573 to 60 920)	137 (104 to 176)	-22.0 (-39.3 to 7.4)	53 585 (38 169 to 73 167)	153 (108 to 208)	-6.1 (-10.2 to -2.1)	100 368 (79 713 to 123 893)	289 (231 to 355)	-14.4 (-25.3 to 0.1)
Palestine	5 606 (3 679 to 7 122)	123 (85 to 150)	-36.9 (-52.5 to -12.8)	6 143 (4 386 to 8 354)	159 (114 to 217)	-1.3 (-3.9 to 1.4)	11 749 (8 989 to 14 630)	282 (220 to 350)	-20.8 (-33.6 to -7.0)
Oman	3 164 (2 475 to 4 059)	82 (66 to 100)	-31.1 (-51.3 to 7.6)	6 211 (4 326 to 8 601)	150 (105 to 208)	-7.6 (-12.1 to -3.3)	9 374 (7 329 to 11 957)	231 (183 to 294)	-17.5 (-29.7 to -2.3)
Qatar	3 911 (1 933 to 5 482)	123 (81 to 161)	-29.2 (-54.2 to 0.2)	5 274 (3 681 to 7 341)	193 (136 to 267)	-12.2 (-15.7 to -8.7)	9 184 (6 461 to 12 013)	317 (243 to 408)	-19.7 (-33.3 to -7.1)
Saudi Arabia	71 193 (50 219 to 98 514)	237 (165 to 324)	-24.3 (-54.7 to 19.7)	60 333 (42 538 to 83 407)	201 (143 to 278)	-22.1 (-26.3 to -17.8)	131 525 (100 977 to 166 670)	438 (336 to 549)	-23.3 (-43.1 to -1.3)
Sudan	62 630 (45 545 to 85 170)	160 (118 to 216)	-31.7 (-55.9 to 15.9)	55 604 (40 799 to 73 076)	181 (134 to 235)	-1.5 (-5.6 to 2.4)	118 234 (92 316 to 148 717)	341 (267 to 422)	-18.5 (-38.5 to 6.4)
Syria	10 415 (8 353 to 12 594)	64 (51 to 78)	-30.2 (-46.3 to -5.9)	23 839 (16 805 to 32 947)	145 (103 to 201)	5.4 (0.3 to 10.8)	34 254 (26 743 to 43 333)	209 (163 to 265)	-8.8 (-18.8 to 1.5)
Tunisia	10 037 (7 350 to 13 740)	88 (64 to 121)	-32.1 (-49.1 to -8.3)	18 078 (12 753 to 24 997)	148 (104 to 204)	-9.1 (-13.7 to -4.0)	28 115 (21 535 to 36 202)	236 (180 to 302)	-19.3 (-29.3 to -8.9)
Turkey	94 737 (83 728 to 105 727)	117 (103 to 132)	-55.4 (-66.4 to -34.5)	130 952 (92 435 to 181 480)	151 (106 to 210)	-31.5 (-36.1 to -26.6)	225 689 (184 708 to 278 346)	268 (221 to 329)	-44.5 (-53.6 to -32.7)
United Arab Emirates	20 352 (10 756 to 29 408)	181 (116 to 243)	-25.5 (-52.0 to 14.3)	21 344 (15 034 to 29 320)	197 (139 to 270)	-26.7 (-30.3 to -23.1)	41 696 (29 158 to 54 903)	378 (281 to 478)	-26.1 (-41.0 to -9.7)
Yemen	45 393 (30 686 to 64 606)	163 (112 to 226)	-25.7 (-51.9 to 59.4)	35 852 (26 034 to 48 122)	159 (116 to 213)	-5.8 (-9.2 to -2.8)	81 245 (61 017 to 105 070)	323 (245 to 409)	-17.0 (-37.2 to 17.3)
**South Asia**	**6 234 779 (5 691 038 to 6 837 136)**	**436 (401 to 482)**	**-12.4 (-24.1 to 5.0)**	**2 920 997 (2 112 287 to 3 879 064)**	**191 (138 to 254)**	**21.3 (18.0 to 24.7)**	**9 155 776 (8 198 592 to 10 227 260)**	**627 (564 to 696)**	**-4.3 (-14.4 to 8.9)**
**South Asia**	**6 234 779 (5 691 038 to 6 837 136)**	**436 (401 to 482)**	**-12.4 (-24.1 to 5.0)**	**2 920 997 (2 112 287 to 3 879 064)**	**191 (138 to 254)**	**21.3 (18.0 to 24.7)**	**9 155 776 (8 198 592 to 10 227 260)**	**627 (564 to 696)**	**-4.3 (-14.4 to 8.9)**
Bangladesh	190 047 (140 862 to 246 333)	138 (108 to 173)	-30.4 (-56.8 to 12.8)	203 154 (145 158 to 273 854)	143 (102 to 192)	21.8 (15.2 to 28.3)	393 201 (312 564 to 487 421)	281 (228 to 343)	-11.0 (-33.3 to 16.9)
Bhutan	2 527 (1 686 to 3 473)	349 (246 to 469)	-30.7 (-49.2 to -1.5)	1 398 (1 011 to 1 874)	178 (129 to 237)	-2.5 (-6.7 to 1.7)	3 925 (3 021 to 5 003)	528 (414 to 663)	-23.2 (-37.4 to -1.4)
India	5 614 927 (5 153 557 to 6 180 884)	500 (460 to 555)	-13.6 (-25.3 to 3.4)	2 405 399 (1 739 948 to 3 195 447)	199 (144 to 264)	19.8 (16.7 to 23.0)	8 020 326 (7 216 843 to 8 914 148)	698 (628 to 775)	-6.1 (-16.4 to 7.3)
Nepal	72 434 (52 704 to 95 796)	311 (232 to 400)	-24.0 (-41.9 to 6.3)	36 708 (26 461 to 49 047)	147 (106 to 197)	6.6 (2.8 to 10.1)	109 142 (85 684 to 135 751)	458 (364 to 560)	-16.3 (-30.8 to 6.3)
Pakistan	354 844 (231 227 to 464 133)	207 (136 to 266)	-3.8 (-29.3 to 27.2)	274 338 (198 293 to 365 294)	173 (125 to 230)	31.7 (27.5 to 36.1)	629 182 (487 063 to 762 998)	380 (296 to 459)	9.6 (-9.4 to 29.2)
**Southeast Asia, East Asia, and Oceania**	**4 820 531 (3 868 525 to 5 229 697)**	**211 (172 to 229)**	**-21.5 (-45.0 to -6.3)**	**3 103 790 (2 197 891 to 4 255 206)**	**123 (87 to 168)**	**53.9 (44.6 to 63.6)**	**7 924 321 (6 522 685 to 9 277 614)**	**334 (278 to 387)**	**-4.3 (-25.9 to 8.7)**
**East Asia**	**3 397 124 (2 502 465 to 3 785 673)**	**208 (157 to 232)**	**-18.8 (-49.2 to -2.7)**	**2 807 526 (1 983 847 to 3 858 130)**	**152 (108 to 209)**	**67.0 (56.7 to 77.7)**	**6 204 650 (4 854 132 to 7 441 190)**	**361 (287 to 430)**	**3.7 (-25.0 to 18.2)**
China	3 238 257 (2 366 246 to 3 626 518)	209 (157 to 234)	-18.9 (-50.1 to -2.3)	2 696 044 (1 905 183 to 3 703 498)	154 (109 to 211)	67.6 (57.0 to 78.5)	5 934 301 (4 623 849 to 7 121 496)	363 (288 to 433)	3.8 (-25.6 to 18.6)
North Korea	56 949 (42 072 to 75 825)	218 (163 to 285)	23.0 (-12.8 to 71.0)	31 885 (22 859 to 43 323)	107 (76 to 144)	73.8 (66.6 to 81.1)	88 834 (71 086 to 111 167)	325 (261 to 405)	36.0 (6.9 to 71.6)
Taiwan (Province of China)	47 192 (43 935 to 50 654)	149 (140 to 160)	-42.4 (-46.6 to -38.1)	34 369 (24 192 to 47 746)	108 (76 to 149)	28.0 (22.2 to 33.6)	81 561 (71 014 to 94 354)	257 (224 to 297)	-25.2 (-31.0 to -19.7)
**Oceania**	**22 505 (15 467 to 30 784)**	**213 (154 to 279)**	**8.8 (-16.5 to 33.7)**	**8 572 (6 230 to 11 524)**	**85 (62 to 114)**	**84.2 (78.1 to 90.0)**	**31 077 (23 277 to 39 776)**	**298 (232 to 372)**	**23.3 (-1.1 to 44.5)**
American Samoa	57 (48 to 64)	118 (101 to 132)	-5.5 (-21.7 to 15.0)	42 (30 to 57)	82 (59 to 112)	51.7 (45.3 to 58.3)	98 (83 to 116)	200 (170 to 234)	11.8 (-2.0 to 28.1)
Federated States of Micronesia	127 (84 to 172)	147 (102 to 193)	2.2 (-30.5 to 39.2)	75 (54 to 101)	82 (59 to 110)	76.2 (68.3 to 84.1)	202 (156 to 257)	229 (180 to 287)	20.2 (-8.3 to 50.5)
Fiji	751 (629 to 882)	91 (77 to 106)	9.4 (-14.2 to 42.6)	680 (488 to 919)	78 (56 to 105)	89.8 (80.3 to 97.7)	1 431 (1 199 to 1 696)	169 (143 to 199)	35.9 (16.2 to 60.2)
Guam	214 (174 to 244)	126 (102 to 144)	33.8 (-6.3 to 71.4)	157 (111 to 217)	89 (63 to 122)	78.2 (70.6 to 85.8)	370 (309 to 439)	215 (179 to 253)	49.2 (17.9 to 74.4)
Kiribati	70 (47 to 90)	69 (49 to 87)	0.6 (-20.1 to 24.8)	78 (57 to 104)	78 (57 to 103)	109.0 (98.5 to 118.4)	147 (114 to 181)	147 (116 to 179)	38.7 (21.3 to 56.6)

Location	YLLs (95% UI)			YLDs (95% UI)			DALYs (95% UI)		
	2017 counts	2017 age-standardised rates per 100,000	Percentage change in age-standardised rates between 1990 and 2017	2017 counts	2017 age-standardised rates per 100,000	Percentage change in age-standardised rates between 1990 and 2017	2017 counts	2017 age-standardised rates per 100,000	Percentage change in age-standardised rates between 1990 and 2017
Marshall Islands	80 (59 to 106)	178 (133 to 229)	14.4 (-15.6 to 48.5)	39 (28 to 52)	81 (58 to 109)	87.1 (78.4 to 95.2)	119 (93 to 149)	259 (205 to 320)	30.3 (2.8 to 56.8)
Northern Mariana Islands	48 (39 to 56)	107 (87 to 123)	0.8 (-28.0 to 31.0)	42 (29 to 58)	80 (56 to 112)	45.6 (39.4 to 51.7)	90 (74 to 108)	187 (156 to 223)	16.1 (-7.6 to 36.3)
Papua New Guinea	15 202 (8 805 to 22 711)	195 (120 to 279)	12.5 (-21.2 to 48.9)	5 790 (4 203 to 7 786)	80 (58 to 107)	95.4 (87.6 to 103.7)	20 992 (14 158 to 28 642)	275 (192 to 362)	28.3 (-5.5 to 58.9)
Samoa	189 (136 to 258)	117 (85 to 157)	6.3 (-23.9 to 40.3)	145 (104 to 196)	88 (63 to 118)	80.4 (72.8 to 87.8)	334 (260 to 424)	204 (160 to 258)	29.1 (2.9 to 53.5)
Solomon Islands	4 077 (3 281 to 4 903)	823 (680 to 973)	-15.8 (-33.8 to 6.2)	759 (551 to 1 003)	162 (117 to 213)	34.4 (31.1 to 37.7)	4 836 (4 037 to 5 653)	986 (843 to 1 131)	-10.3 (-27.4 to 9.7)
Tonga	82 (70 to 96)	94 (80 to 110)	-3.7 (-22.7 to 21.2)	75 (54 to 102)	82 (59 to 111)	77.2 (68.7 to 85.3)	157 (132 to 187)	176 (149 to 209)	22.3 (6.3 to 40.6)
Vanuatu	368 (245 to 525)	158 (107 to 219)	17.5 (-21.7 to 65.7)	219 (159 to 294)	97 (70 to 129)	91.4 (83.9 to 98.2)	587 (440 to 760)	255 (195 to 323)	37.7 (2.7 to 74.0)
**Southeast Asia**	**1 400 902 (1 309 912 to 1 542 331)**	**232 (217 to 253)**	**-29.9 (-39.2 to -14.9)**	**287 692 (208 176 to 385 489)**	**45 (32 to 60)**	**1.8 (-2.4 to 6.2)**	**1 688 594 (1 565 442 to 1 846 980)**	**277 (257 to 301)**	**-26.2 (-35.0 to -12.6)**
Cambodia	50 273 (42 496 to 60 395)	383 (328 to 450)	-15.7 (-35.0 to 11.7)	8 257 (6 021 to 11 051)	60 (44 to 80)	36.0 (31.2 to 40.8)	58 529 (50 520 to 68 410)	443 (386 to 512)	-11.1 (-29.5 to 14.0)
Indonesia	509 835 (471 215 to 582 918)	233 (217 to 259)	-42.9 (-51.5 to -26.8)	86 103 (63 399 to 114 956)	35 (26 to 47)	-33.2 (-35.4 to -31.3)	595 938 (547 572 to 668 297)	268 (248 to 295)	-41.8 (-49.6 to -27.9)
Laos	15 097 (10 729 to 18 642)	259 (193 to 320)	-12.2 (-43.4 to 27.9)	2 992 (2 178 to 4 000)	52 (38 to 69)	50.9 (44.7 to 56.8)	18 089 (13 602 to 21 769)	311 (242 to 374)	-5.5 (-36.5 to 30.9)
Malaysia	21 467 (16 885 to 25 363)	79 (62 to 92)	-24.4 (-40.6 to 6.0)	12 333 (8 789 to 16 933)	42 (30 to 57)	43.8 (34.8 to 52.6)	33 800 (28 136 to 39 558)	121 (101 to 140)	-9.5 (-24.9 to 15.8)
Maldives	410 (349 to 520)	111 (97 to 129)	-51.9 (-67.5 to -16.0)	181 (128 to 249)	43 (30 to 59)	7.4 (0.1 to 14.9)	590 (505 to 706)	153 (134 to 176)	-43.2 (-59.5 to -10.8)
Mauritius	2 039 (1 825 to 2 256)	139 (125 to 154)	28.6 (13.0 to 44.4)	700 (496 to 965)	46 (32 to 63)	44.0 (37.1 to 50.6)	2 739 (2 450 to 3 060)	185 (166 to 206)	32.1 (19.6 to 44.7)
Myanmar	202 682 (170 674 to 243 608)	423 (362 to 499)	-24.2 (-44.4 to 2.1)	30 599 (22 257 to 40 838)	61 (44 to 81)	27.5 (22.3 to 32.4)	233 281 (201 357 to 274 562)	483 (418 to 562)	-20.1 (-39.7 to 4.7)
Philippines	138 211 (121 329 to 157 940)	155 (137 to 175)	44.4 (26.3 to 65.9)	36 921 (26 428 to 50 029)	41 (29 to 55)	67.4 (59.6 to 75.5)	175 131 (156 260 to 198 222)	196 (174 to 220)	48.7 (33.6 to 66.0)
Sri Lanka	52 358 (41 342 to 64 975)	231 (184 to 283)	-16.4 (-37.8 to 7.1)	11 711 (8 269 to 16 129)	49 (35 to 68)	27.1 (20.4 to 33.8)	64 069 (52 641 to 77 638)	280 (232 to 337)	-11.0 (-30.9 to 9.7)
Seychelles	191 (157 to 219)	174 (145 to 200)	-7.8 (-25.9 to 9.1)	55 (39 to 76)	50 (35 to 68)	36.9 (30.0 to 44.0)	245 (208 to 279)	224 (191 to 254)	-0.7 (-17.4 to 14.6)
Thailand	105 080 (85 240 to 121 275)	123 (100 to 142)	-42.2 (-54.3 to -23.2)	39 279 (27 713 to 54 266)	44 (31 to 61)	14.4 (7.4 to 21.0)	144 359 (120 699 to 166 601)	167 (141 to 193)	-33.5 (-45.3 to -17.0)
Timor-Leste	2 166 (1 402 to 2 699)	205 (140 to 257)	-4.1 (-37.2 to 37.5)	499 (363 to 671)	50 (37 to 67)	56.7 (49.2 to 64.3)	2 666 (1 918 to 3 206)	256 (190 to 307)	3.9 (-28.3 to 41.0)
Vietnam	299 251 (254 572 to 352 194)	327 (282 to 381)	-8.2 (-27.1 to 20.9)	57 685 (40 942 to 78 882)	59 (42 to 81)	32.1 (24.9 to 39.5)	356 935 (308 271 to 412 763)	387 (338 to 445)	-3.7 (-21.0 to 21.8)
**Sub-Saharan Africa**	**1 283 950 (1 143 167 to 1 528 381)**	**189 (169 to 221)**	**-19.0 (-29.2 to -1.4)**	**1 229 845 (893 400 to 1 641 170)**	**184 (134 to 245)**	**-2.6 (-4.3 to -1.1)**	**2 513 796 (2 134 544 to 2 937 994)**	**374 (316 to 436)**	**-11.7 (-18.4 to -2.2)**
**Central sub-Saharan Africa**	**132 699 (99 495 to 208 538)**	**159 (129 to 224)**	**-15.0 (-30.7 to 12.5)**	**128 392 (93 308 to 170 291)**	**167 (121 to 222)**	**3.5 (1.3 to 5.5)**	**261 092 (208 186 to 339 885)**	**326 (267 to 404)**	**-6.5 (-17.4 to 7.8)**
Angola	34 760 (26 892 to 50 985)	189 (150 to 261)	-23.9 (-46.4 to 19.0)	29 496 (21 418 to 39 602)	184 (134 to 245)	-3.8 (-6.1 to -1.5)	64 256 (51 804 to 81 692)	374 (303 to 456)	-15.1 (-31.2 to 6.4)
Central African Republic	6 146 (3 475 to 12 051)	186 (121 to 316)	-8.4 (-31.7 to 23.6)	5 023 (3 653 to 6 672)	156 (114 to 208)	11.5 (8.9 to 14.3)	11 169 (7 827 to 17 407)	343 (259 to 475)	-0.2 (-14.3 to 17.6)
Congo (Brazzaville)	5 854 (3 856 to 9 027)	171 (112 to 261)	-22.7 (-43.7 to 6.6)	5 942 (4 325 to 7 956)	170 (123 to 228)	-2.7 (-5.0 to -0.6)	11 796 (9 031 to 15 279)	341 (264 to 439)	-13.9 (-27.9 to 2.0)
DR Congo	82 545 (54 823 to 138 829)	147 (109 to 213)	-11.4 (-30.0 to 19.1)	84 273 (61 487 to 111 188)	162 (118 to 213)	6.4 (3.7 to 9.0)	166 818 (128 290 to 224 729)	309 (249 to 388)	-2.9 (-14.2 to 12.2)
Equatorial Guinea	1 196 (704 to 2 031)	150 (91 to 248)	-30.9 (-62.3 to 13.6)	1 264 (910 to 1 705)	166 (119 to 222)	6.6 (1.4 to 11.7)	2 460 (1 842 to 3 253)	316 (237 to 410)	-15.3 (-39.1 to 11.6)
Gabon	2 198 (1 550 to 3 440)	171 (123 to 257)	-22.1 (-43.4 to 0.9)	2 394 (1 729 to 3 201)	185 (135 to 247)	-13.2 (-15.6 to -10.9)	4 592 (3 607 to 5 872)	356 (283 to 446)	-17.7 (-29.9 to -6.2)
**Eastern sub-Saharan Africa**	**534 844 (473 936 to 600 996)**	**227 (206 to 251)**	**-20.6 (-33.7 to 0.6)**	**586 065 (426 189 to 777 264)**	**241 (176 to 319)**	**0.7 (-0.8 to 2.1)**	**1 120 909 (939 862 to 1 332 942)**	**468 (396 to 552)**	**-10.9 (-19.8 to 0.9)**
Burundi	13 690 (10 621 to 17 492)	225 (176 to 282)	-30.2 (-46.1 to -2.1)	15 009 (10 798 to 19 921)	222 (162 to 294)	-7.1 (-9.0 to -4.8)	28 699 (23 307 to 34 895)	447 (364 to 538)	-20.4 (-30.9 to -4.9)
Comoros	920 (718 to 1 225)	177 (142 to 234)	-27.3 (-41.9 to -4.2)	1 330 (966 to 1 760)	235 (170 to 311)	-8.7 (-11.2 to -6.5)	2 250 (1 817 to 2 744)	412 (336 to 495)	-17.8 (-26.0 to -6.8)
Djibouti	1 320 (912 to 1 941)	187 (135 to 265)	-21.4 (-43.9 to 9.4)	1 994 (1 447 to 2 657)	242 (176 to 322)	-10.1 (-12.6 to -7.6)	3 314 (2 616 to 4 204)	429 (343 to 531)	-15.4 (-27.7 to -2.0)
Eritrea	7 992 (5 855 to 11 123)	231 (171 to 311)	-18.4 (-40.3 to 16.6)	8 765 (6 359 to 11 596)	232 (169 to 307)	-0.1 (-2.5 to 2.2)	16 758 (13 325 to 20 375)	462 (370 to 560)	-10.2 (-24.9 to 7.8)
Ethiopia	144 032 (120 361 to 169 974)	238 (203 to 276)	-33.1 (-52.0 to -3.5)	139 331 (101 708 to 185 584)	228 (166 to 301)	-4.8 (-6.9 to -2.9)	283 363 (232 563 to 337 663)	466 (388 to 551)	-21.7 (-36.6 to -3.9)
Kenya	63 203 (55 217 to 76 054)	230 (205 to 270)	-7.2 (-31.4 to 15.3)	96 950 (70 610 to 128 483)	298 (219 to 392)	8.5 (6.3 to 10.8)	160 153 (132 620 to 193 453)	528 (440 to 630)	1.0 (-15.1 to 11.4)
Madagascar	25 844 (19 652 to 34 352)	166 (129 to 215)	-28.9 (-43.5 to -8.6)	38 628 (27 879 to 51 439)	230 (166 to 305)	-5.5 (-8.0 to -2.8)	64 472 (51 826 to 79 083)	396 (317 to 480)	-17.0 (-25.1 to -7.2)
Malawi	27 799 (23 145 to 33 643)	252 (215 to 298)	-12.8 (-34.3 to 47.8)	24 306 (17 634 to 32 346)	221 (161 to 294)	-0.8 (-3.6 to 1.8)	52 105 (44 049 to 62 316)	474 (402 to 556)	-7.6 (-21.9 to 20.7)
Mozambique	52 374 (37 530 to 64 915)	305 (222 to 380)	-8.0 (-26.6 to 17.4)	41 139 (29 718 to 54 899)	237 (171 to 314)	-2.7 (-6.1 to 0.7)	93 513 (74 328 to 113 460)	542 (433 to 653)	-5.8 (-17.5 to 8.4)
Rwanda	15 256 (11 881 to 20 085)	200 (160 to 255)	-36.8 (-53.5 to -7.7)	18 739 (13 624 to 25 032)	224 (163 to 298)	-9.9 (-12.1 to -7.8)	33 995 (27 221 to 41 451)	424 (345 to 510)	-24.9 (-36.1 to -8.5)
Somalia	18 610 (12 756 to 26 794)	193 (142 to 265)	-12.0 (-39.1 to 47.4)	24 328 (17 775 to 31 959)	234 (170 to 308)	9.2 (6.9 to 11.4)	42 939 (33 286 to 53 297)	427 (335 to 525)	-1.5 (-19.4 to 24.5)
South Sudan	12 594 (9 337 to 17 194)	207 (154 to 279)	-8.6 (-34.4 to 42.2)	14 496 (10 530 to 19 244)	241 (176 to 319)	3.4 (0.9 to 5.7)	27 090 (21 626 to 33 147)	448 (356 to 548)	-2.5 (-18.8 to 18.5)
Tanzania	86 274 (71 874 to 105 749)	227 (197 to 267)	-12.0 (-29.4 to 23.3)	83 589 (60 534 to 110 655)	243 (176 to 322)	0.2 (-2.3 to 2.6)	169 862 (141 505 to 203 779)	470 (395 to 555)	-6.0 (-16.6 to 10.9)
Uganda	36 951 (27 833 to 47 700)	169 (130 to 210)	-12.7 (-35.9 to 17.1)	51 097 (37 014 to 68 263)	224 (162 to 296)	17.5 (14.7 to 20.5)	88 048 (70 556 to 107 418)	393 (317 to 476)	2.3 (-14.1 to 17.5)
Zambia	27 650 (23 300 to 32 623)	274 (236 to 316)	-21.3 (-38.7 to 7.4)	25 996 (18 913 to 34 499)	252 (182 to 334)	-3.6 (-5.8 to -1.4)	53 645 (44 834 to 63 373)	525 (446 to 609)	-13.7 (-25.3 to 2.1)
**Southern sub-Saharan Africa**	**41 632 (37 637 to 46 623)**	**66 (61 to 75)**	**-17.3 (-33.3 to -3.3)**	**78 614 (56 207 to 106 673)**	**117 (84 to 157)**	**2.5 (0.1 to 4.9)**	**120 245 (97 464 to 148 485)**	**183 (150 to 224)**	**-5.7 (-14.3 to 0.6)**
Botswana	1 129 (812 to 1 536)	71 (53 to 93)	-26.8 (-46.8 to -1.7)	2 140 (1 529 to 2 914)	114 (82 to 155)	-4.6 (-8.2 to -1.2)	3 269 (2 517 to 4 124)	185 (146 to 228)	-14.5 (-25.1 to -3.6)
Lesotho	1 598 (1 157 to 2 072)	114 (84 to 146)	2.8 (-34.6 to 48.9)	1 831 (1 325 to 2 454)	117 (84 to 156)	7.6 (4.5 to 10.6)	3 429 (2 691 to 4 171)	230 (183 to 279)	5.2 (-17.2 to 26.9)
Namibia	1 444 (1 169 to 1 805)	87 (73 to 107)	-21.6 (-36.7 to -0.4)	2 182 (1 578 to 2 955)	118 (86 to 160)	-7.2 (-10.4 to -3.9)	3 626 (2 904 to 4 441)	206 (167 to 248)	-14.0 (-22.8 to -4.4)
South Africa	26 015 (22 281 to 29 949)	54 (47 to 62)	-24.6 (-35.8 to -11.6)	57 918 (41 363 to 78 786)	114 (82 to 154)	1.1 (-1.9 to 4.1)	83 934 (66 336 to 105 698)	168 (133 to 210)	-8.9 (-16.2 to -3.2)
Swaziland	723 (536 to 941)	99 (75 to 127)	-13.3 (-39.3 to 21.6)	951 (683 to 1 284)	115 (84 to 156)	-2.4 (-5.4 to 0.9)	1 674 (1 337 to 2 041)	214 (171 to 258)	-7.7 (-22.1 to 8.7)
Zimbabwe	10 723 (7 873 to 13 123)	125 (91 to 160)	13.3 (-23.9 to 47.6)	13 591 (9 853 to 18 102)	133 (96 to 177)	13.0 (10.1 to 16.2)	24 314 (19 437 to 29 220)	258 (207 to 306)	13.1 (-6.1 to 28.7)
**Western sub-Saharan Africa**	**574 775 (486 326 to 733 838)**	**199 (167 to 246)**	**-20.1 (-31.1 to -2.9)**	**436 774 (316 033 to 584 559)**	**159 (116 to 212)**	**-10.5 (-12.7 to -8.5)**	**1 011 550 (853 388 to 1 189 453)**	**358 (299 to 419)**	**-16.1 (-23.0 to -6.5)**
Benin	13 189 (10 177 to 16 743)	184 (146 to 227)	-25.8 (-40.9 to -5.7)	11 574 (8 429 to 15 402)	158 (115 to 209)	-8.4 (-10.9 to -5.9)	24 763 (20 300 to 29 555)	342 (281 to 405)	-18.7 (-28.7 to -7.0)
Burkina Faso	45 026 (36 747 to 55 273)	309 (268 to 357)	-15.1 (-28.9 to 4.1)	23 871 (17 280 to 31 719)	187 (136 to 248)	-7.1 (-9.9 to -4.6)	68 898 (58 442 to 82 268)	496 (429 to 577)	-12.3 (-21.7 to -0.3)
Cameroon	38 123 (29 507 to 48 142)	229 (183 to 281)	-20.9 (-36.8 to -2.4)	28 938 (20 874 to 38 671)	162 (118 to 216)	-14.0 (-16.2 to -11.6)	67 060 (54 546 to 79 559)	390 (322 to 462)	-18.1 (-28.1 to -7.0)
Cape Verde	246 (212 to 282)	49 (43 to 57)	-33.9 (-47.2 to -15.5)	669 (477 to 914)	133 (95 to 181)	-1.7 (-5.5 to 2.2)	914 (719 to 1 161)	182 (144 to 231)	-13.1 (-20.7 to -5.3)
Chad	22 111 (17 040 to 28 994)	223 (167 to 303)	-3.1 (-21.4 to 20.1)	14 362 (10 435 to 18 994)	166 (121 to 219)	4.7 (2.2 to 7.3)	36 473 (29 726 to 44 687)	388 (311 to 482)	0.0 (-11.4 to 13.5)
Cote d'Ivoire	35 188 (27 605 to 45 054)	224 (180 to 284)	-15.1 (-32.9 to 5.9)	27 690 (20 107 to 36 827)	167 (121 to 221)	-8.1 (-10.3 to -5.9)	62 877 (51 849 to 75 590)	391 (326 to 467)	-12.3 (-23.5 to -0.1)
The Gambia	3 665 (2 829 to 4 752)	284 (229 to 346)	-7.6 (-29.2 to 16.6)	2 176 (1 590 to 2 927)	154 (112 to 206)	-6.5 (-9.1 to -4.2)	5 842 (4 759 to 7 165)	438 (365 to 518)	-7.2 (-21.8 to 8.2)
Ghana	54 049 (44 739 to 64 175)	281 (236 to 323)	8.3 (-17.0 to 36.9)	36 843 (26 767 to 49 307)	173 (126 to 230)	-0.3 (-3.6 to 2.9)	90 892 (76 086 to 107 966)	453 (389 to 526)	4.9 (-11.3 to 20.9)
Guinea	16 674 (13 608 to 21 047)	212 (176 to 259)	-18.2 (-35.1 to 0.4)	12 515 (9 117 to 16 651)	162 (118 to 213)	-9.5 (-12.0 to -7.0)	29 189 (24 443 to 34 611)	374 (311 to 438)	-14.7 (-26.0 to -4.1)
Guinea-Bissau	2 508 (1 940 to 3 399)	225 (184 to 293)	-28.4 (-43.8 to -6.3)	1 797 (1 310 to 2 394)	151 (109 to 201)	-13.0 (-15.2 to -10.7)	4 305 (3 492 to 5 314)	376 (313 to 452)	-22.9 (-33.9 to -8.9)
Liberia	4 732 (3 415 to 6 332)	167 (125 to 214)	-36.4 (-50.6 to -13.9)	4 663 (3 386 to 6 218)	146 (106 to 193)	-11.8 (-14.4 to -9.2)	9 395 (7 415 to 11 636)	313 (251 to 381)	-26.9 (-37.3 to -13.1)
Mali	27 762 (20 300 to 37 237)	184 (141 to 256)	-28.4 (-43.3 to -3.8)	18 884 (13 629 to 25 255)	153 (110 to 204)	-9.0 (-11.9 to -6.1)	46 646 (36 887 to 57 982)	337 (272 to 419)	-20.7 (-30.9 to -6.1)
Mauritania	4 362 (3 161 to 6 029)	163 (123 to 220)	-28.4 (-44.0 to -2.2)	4 281 (3 090 to 5 745)	158 (115 to 211)	-15.7 (-18.2 to -13.0)	8 643 (6 860 to 10 663)	321 (261 to 392)	-22.6 (-31.4 to -8.3)
Niger	23 736 (16 631 to 34 106)	176 (126 to 264)	-34.2 (-51.0 to -10.7)	19 008 (13 780 to 25 463)	158 (114 to 210)	-9.5 (-12.1 to -6.8)	42 744 (33 584 to 54 166)	333 (261 to 430)	-24.5 (-36.3 to -9.9)
Nigeria	244 838 (177 615 to 355 248)	171 (122 to 241)	-26.4 (-45.3 to 0.5)	196 819 (141 458 to 264 282)	153 (111 to 205)	-13.9 (-16.9 to -11.1)	441 656 (353 720 to 550 981)	324 (257 to 402)	-21.0 (-32.2 to -6.3)
Sao Tome and Principe	493 (380 to 637)	324 (237 to 423)	-4.2 (-28.4 to 24.4)	323 (233 to 430)	224 (161 to 299)	-13.0 (-16.2 to -10.2)	816 (659 to 982)	548 (441 to 663)	-8.0 (-21.5 to 7.6)
Senegal	19 513 (14 212 to 24 872)	197 (151 to 243)	-19.1 (-35.3 to 1.8)	16 456 (12 007 to 21 864)	163 (119 to 217)	-9.3 (-11.8 to -7.0)	35 969 (28 485 to 43 582)	360 (293 to 431)	-15.0 (-25.0 to -3.4)
Sierra Leone	10 444 (8 192 to 13 601)	205 (168 to 256)	-21.6 (-38.9 to 3.7)	7 911 (5 802 to 10 546)	151 (110 to 201)	-9.9 (-12.3 to -7.6)	18 356 (15 052 to 22 106)	356 (294 to 423)	-17.1 (-29.1 to -1.9)
Togo	8 111 (6 357 to 10 549)	176 (142 to 221)	-26.0 (-41.0 to -7.6)	7 989 (5 777 to 10 703)	151 (109 to 202)	-9.7 (-12.4 to -7.0)	16 100 (13 149 to 19 364)	326 (270 to 388)	-19.3 (-28.9 to -8.7)
